# The Mental Health Impacts of Wildfire Exposure: A Scoping Review of the Quantitative Evidence

**DOI:** 10.1007/s40572-026-00547-5

**Published:** 2026-08-17

**Authors:** Katharine A. Teigen, David M. Coomes, Amruta Nori-Sarma, Joan A. Casey

**Affiliations:** 1 Department of Environmental and Occupational Health Sciences, University of Washington, Seattle, USA; 2 Department of Epidemiology, University of Washington, Seattle, USA; 3 Department of Environmental Health, Harvard T.H. Chan School of Public Health, Boston, USA

**Keywords:** Wildfires, Mental health, Depression, Stress disorders, Post-traumatic, Anxiety, Substance-related disorders

## Abstract

Wildfires are increasing in frequency and intensity worldwide, with growing evidence linking wildfire exposure to adverse mental health outcomes. This scoping review aimed to summarize and critically assess the existing quantitative literature on wildfire-related mental health impacts. We searched PubMed, Web of Science, and Google Scholar to identify studies published between 2000 and 2025, which we analyzed to understand how wildfires were investigated, evaluate how exposure was measured, assess mental health outcomes, and identify persistent gaps in the literature. Of 251 unique manuscripts identified, 85 met inclusion criteria. Studies were primarily conducted in Australia (34.1%), Canada (31.8%), and the United States (29.4%), with limited representation from Greece, Italy, Israel, South Korea, and Spain. Exposure assessment methods varied widely, where 21.2% of studies considered everyone uniformly exposed. Other exposure assessment methods used were evacuation status, questionnaires, researcher-ascribed loss severity, and spatio-temporal proximity. Depression was the most frequently studied outcome (evaluated in 58.5% of studies), followed by post-traumatic stress disorder (52.9%) and anxiety (48.2%). Other outcomes included insomnia, suicidality, substance use, and resilience. Most studies reported elevated adverse mental health following wildfires, though findings varied. Multiple aspects of wildfire exposure, including direct exposure, seeing flames or smoke, and being evacuated were consistently associated with adverse mental health outcomes. Limitations in the literature included non-standardized exposure assessments, limited geographic diversity, and a shortage of rigorous epidemiologic study designs. Addressing these gaps is essential to improving understanding of wildfire-related mental health impacts and informing public health responses.

## Introduction

### History

Wildfires are becoming one of the most devastating and expensive climate hazards worldwide, with property losses from the 2025 Los Angeles Fires estimated as upwards of $45 billion [1–3]. In places like the United States (US), Canada, and Australia, the increasing threat of wildfire is due to a century of wildfire suppression, climate change, and expanding residential development in the wildland-urban interface (WUI), the zone where homes and wildland vegetation meet or intermingle [4]. For centuries prior to colonization, Indigenous peoples across the US, Canada, and Australia used fire management practices, termed “cultural burning,” to maintain the health of their ecosystems while minimizing the threat of catastrophic wildfires [5, 6]. Upon European colonization, Indigenous land use practices decreased dramatically, with the aggressive implementation of fire suppression practices from the early 1900s to the 1980s [7]. Today, most land management practices use prescribed burns and even incorporate some partnerships with tribal organizations, and in 2022, California enacted a rule that removes liability risk for private citizens and Indigenous people who engage in controlled burning [8]. Nonetheless, ecosystems and affected communities across the globe are still suffering the consequences of almost a century of biomass accumulation.

### Wildfire Incidence and Population Exposure

Wildfires are increasing in frequency and intensity worldwide. Driven by anthropogenic climate change, shifts in seasonality, drought, fire weather patterns, and fuel moisture are extending global fire seasons across biogeographical regions [9, 10]. In Australia, there was an increasing linear trend in burned area from 1980 to 2020, as well as a marked increase in the frequency of megafires (10,000 km^2^+) since 2000 [11]. During the summer of 2023, wildfires in Canada burned at record-setting intensity and pace, ultimately burning 5% of the total forested area in the country [12]. Since 2000, wildfires in the US have burned an average of 7 million acres every year, which is more than double the annual average of acreage burned in the 1990s [13].

A positive feedback loop exists between wildfires and climate change. Climate change results in more frequent and longer droughts, creating conditions with less moisture and more dried vegetation to fuel wildfires. When wildfires occur, emissions and the destruction of trees that remove carbon dioxide from the atmosphere contribute to the greenhouse effect, worsening climate change [9]. In fact, climate change is beginning to impact wildfire incidence and severity in ways beyond the conventional drought mechanism. The devastation of the 2023 Maui wildfire was, in part, caused by high gale-force winds from a distant hurricane [14]. Due to these winds, when sparks caught flame in the town of Lahaina, they spread rapidly and uncontrollably. The Santa Ana winds also played a role in the 2025 Los Angeles Fires [15]. Additionally, climate change is altering the seasonality of wildfire events. Historically, wildfire season occurred during the summer and fall months. New, prolonged drought conditions driven by climate change are lengthening this “season,” with substantial events happening during winter and spring months. For example, the 2025 Los Angeles Fires burned throughout January, with estimates suggesting that the exceptionally hot and dry conditions preceding the fires were made 35% more likely by climate change [16].

The number of people impacted by wildfires is also increasing, with certain sub-groups at greater risk than others. The number of houses in the US WUI has increased by 350,000 every year, with a total of 50 million houses at heightened risk of wildfire damage in 2021 [17, 18]. While higher-income households have historically been overrepresented among the homes at higher wildfire risk [19], this pattern will likely shift as the geographic scope of wildfire damage increases and homes located in the WUI become less desirable and insurable [20]. Notably, housing values have decreased in areas directly affected by wildfires [21], as well as in neighboring areas [22], although these trends are not consistent across all geographic areas. Households with a perceived greater risk of experiencing a wildfire may now be devalued, while properties with greater resilience to such events are favored by investors and developers [23].

Who has the ability to recover from a wildfire and on what timescale are also important exposure considerations. For instance, federal aid for the 2018 Camp Fire in Paradise, California, may have contributed to displacing socially and economically disadvantaged residents who were unable to access disaster recovery benefits [24]. Even when wealthier households are more exposed, those with fewer resources may be less prepared or able to respond. One study of wildfires found that perceived cost, lack of time, and physical ability were key barriers to taking steps to reduce wildfire risk around one’s home [25]. In Washington state, poorer households at risk of wildfire were more likely to be in fire districts with less suppression capability than wealthier communities [26]. Finally, evidence from select Southern California fires suggests that lower-priced properties may sustain more fire damage than higher-priced properties [27]. Collectively, these findings highlight both the differential wildfire vulnerabilities of these increasingly exposed households and the need to further characterize such exposure-relevant metrics across wildfire contexts.

Individuals may be exposed to a wildfire event in numerous ways. During primary exposure, individuals experience direct contact with a wildfire. This could be via actual burns, inhalation of smoke, or having to evacuate [28]. Individuals may also experience a wildfire event through secondary exposure, in which they are indirectly affected by the fire. For example, secondary exposures could include losing one’s home and property to a wildfire or having a close friend or relative who was personally impacted by a wildfire. Finally, tertiary exposure does not involve direct or personal experiences; rather, the event is experienced through an intermediary, such as watching news coverage of a wildfire on television. In all types of exposure, the stress and trauma of a wildfire may negatively impact individuals’ mental health in the short and long term.

### Mental Health Outcomes

The American Psychological Association defines mental health as “a state of mind characterized by emotional well-being, good behavioral adjustment, relative freedom from anxiety and disabling symptoms, and a capacity to establish constructive relationships and cope with the ordinary demands and stresses of life” [29]. Globally, in 2019, 12% of all disability-adjusted life years, a measure of overall disease burden, were driven by mental health disorders [30], and these are partially driven by environmental factors. Specifically, wildfire events can negatively impact mental health through myriad pathways, including inducing neuroinflammation from wildfire smoke which can alter normal neurotransmitter levels [31], triggering the body’s “fight-or-flight” response which could lead to prolonged anxiety or stress [32], and disrupting sleep which may result in mood disturbances [33]. Further, many individual-level factors – economic stability, sociodemographic characteristics, and social connections [19] – can modify the relationship between wildfire exposure and adverse mental health outcomes. Timing of poor mental health onset following wildfire exposure is also uncertain, which can complicate evaluations of mental health status, especially in non-clinical settings. Thus, mental health is a critical outcome to investigate in the context of wildfire exposure, especially as the incidence of poor mental health, as exacerbated by wildfires, is expected to increase with climate change [34]. While studies have linked wildfire exposure to adverse mental health outcomes, including depression, anxiety, and substance use disorder, these studies require summary and critical analysis to identify key next steps in this line of inquiry [35].

### Purpose

The purpose of this scoping review is to summarize the existing peer-reviewed, quantitative evidence of our understanding about community-level mental health impacts of wildfire exposure. Additionally, this work builds upon a previous scoping review conducted on the impacts of wildfires on mental health [36] by including all mental health consequences identified in the literature (i.e., expanding beyond post-traumatic stress disorder (PTSD), depression, anxiety, and substance use alone) and critically appraising the quantitative methods used to investigate this association (i.e., exposure assessment, outcome ascertainment, and statistical analysis including confounder adjustment and exploration of effect modifiers). We aim to identify which wildfires have resulted in scientific investigation of mental health outcomes, characterize mental health-relevant exposure metrics, evaluate which mental health outcomes appear to increase after exposure and at what time intervals, and make recommendations about persistent gaps in the literature and future research priorities. In doing so, we hope to clarify why some fires’ mental health impacts are investigated extensively while others go unstudied or understudied. Consequently, our goal is to elucidate what geographic regions or subpopulations require further investigation to better understand the relationship between wildfire exposure and mental health. As climate change continues to exacerbate the frequency and magnitude of wildfires, there is an ever pressing need to fill this research gap.

## Methods

An initial literature search conducted by Teigen, K. was performed in PubMed and Web of Science on July 15, 2023. An updated search was performed on January 15, 2025. All searches were restricted to articles that had been published from 2000 to 2025. PubMed search terms included (((wildfire)) AND ((“mental health”) OR (stress) OR (anxiety) OR (mood) OR (“substance use”) OR (“behavioral disorder”) OR (schizophrenia))) AND (“mental health”). Web of Science search terms included ((TS=(wildfire)) AND TS=(((“mental health”) OR (stress) OR (anxiety) OR (mood) OR (“substance use”) OR (“behavioral disorder”) OR (schizophrenia)))) AND TS=((“mental health”)). Google Scholar and reference lists were also reviewed for additional relevant studies.

Prior to screening, duplicates and non-English records were removed. Only primary articles published between the years of 2000 and 2025 that assessed exposure to a wildfire event and a mental health outcome were included. We focused on which analytical methods have been employed to study this topic, and thus limited our review to articles that were hypothesis-based and conducted quantitative analyses. This criterion was developed at the outset of the study because we intended to conduct a meta-analysis; however, due to study heterogeneity and small sample sizes, we were unable to perform this analysis. Purely descriptive studies, such as qualitative, narrative, and review articles, were excluded. While these studies are a critical tool to evaluate mental health needs, especially when diagnostic methods are unavailable, analyzing qualitative studies was beyond the scope of this review. The primary reviewer then evaluated all remaining articles and extracted the relevant data, including the wildfire event, data collection methods, timing of data collection post-fire, mental health outcomes evaluated, exposure measurement methods, outcome assessment methods, statistical methods, confounding adjustment, prevalence rates and/or association type (i.e., positive, negative, or null), and effect modifiers or risk factors. Some studies included additional exposures beyond wildfire events. In our review, we only describe and summarize the mental health outcomes that were evaluated in association with wildfire exposure.

To best extract and display the relevant information from the literature, we categorized studies according to whether they used a comparison group to test the relationship between wildfire exposure-related variables and mental health outcomes (Table 1) (*n* = 67) or whether they considered their entire study population exposed, and were thus limited to reporting prevalences of the mental health outcomes among the wildfire-exposed study population (Table 2) (*n* = 18). In this second group of studies with all-exposed study populations, authors could not test for effect measure modification because there was no variation in wildfire exposure in the study population. Thus, while we were able to identify effect modifiers for the studies in Table 1, variables that were associated with the mental health outcomes of interest among study populations of wildfire-affected areas are described as risk factors in Table 2.

**Table 1:** Included studies with an explicitly defined exposed population

Author	Study type	Data collection methods	Mental health outcomes	Exposure measurement	Outcome measurement	Statistical methods	Confounding adjustment	Prevalence and/or association type: + positive × null - negative	Effect modifiers
Year	Size								
Title	Population	Time of collection post-fire							
**Los Angeles Wildfires (2025)**									
Lee et al. [37] 2025 Depression, Anxiety, and PTSD Among Southern California Residents After the January 2025 Los Angeles Wildfires	Cross-sectional 635 Current or former students, staff, and faculty from the University of Southern California who had participated in a study of COVID-19 and mental health in 2021–2022 and lived in California in 2025	Online survey 2–3 months	Anxiety, depression, PTSD	Binary evacuation status provided via self-report	Center of Epidemiologic Studies Depression Scale, 10-item version (CES-D-10), Generalized Anxiety Disorder 7-item (GAD-7), Primary Care PTSD Screen for the Diagnostic and Statistical Manual of Mental Disorders, Fifth Edition (DSM-5) (PC-PTSD-5)	Bivariate and multivariate logistic regression models	Demographic information and baseline CES-D-10 and GAD-7 scores (obtained from participation in previous study)	15.7% moderate to severe anxiety 29.6% moderate to severe depression 11.3% moderate to severe PTSD Evacuation status: + depression, PTSD × anxiety	Not Assessed
Casey et al. [38] 2025 The 2025 Los Angeles Wildfires and Outpatient Acute Health Care Utilization	Cohort 3.7 million electronic healthcare records (305,258 highly exposed and 1.4 million moderately exposed) Members of Kaiser Permanente Southern California	All secondary data sources N/A	Daily neuropsychiatric outpatient and virtual acute care visits on January 7, 2025 (i.e., the fires’ ignition date), compared to those in the subsequent week	Highly exposed members were defined as those who resided in census tracts within 20 km of burn zones. Moderately exposed members were defined as those who resided in tracts within Los Angeles County that were 20 km or more miles from the burn zones.	International Classification of Diseases, Tenth Revision (ICD-10) codes	Two-stage interrupted time-series analysis coupled with machine learning algorithms	Time-varying covariates: daily maximum and minimum temperature and humidity, wind velocity, and surface downward shortwave radiation; weekly wastewater surveillance data on influenza, respiratory syncytial virus, and SARS-CoV-2	Comparing visits on January 7, 2025 to those in the subsequent week: Among highly exposed members: **+** outpatient neuropsychiatric visits (31% higher than expected) Among moderately exposed members: **+** outpatient neuropsychiatric visits (28% higher than expected)	Not Assessed
**Canadian Wildfires (2023)**									
Obuobi-Donkor et al. [39] 2024 2023 Wildfires in Canada: Living in Wildfire Regions in Alberta and Nova Scotia Doubled the Odds for Residents to Experience Likely Generalized Anxiety Disorder Symptoms	Cross-sectional 298 Residents of Alberta and Nova Scotia during the wildfires who had subscribed to the Text4Hope mental health support services	Online survey During the wildfires, 2–3 months after their ignition in March 2023	Generalized Anxiety Disorder (GAD)	Participants self-reported if they lived in a region of Alberta or Nova Scotia that had recently been impacted by the 2023 wildfires.	GAD-7	Chi-squared/ Fisher’s exact tests, logistic regression	Age, employment status, housing status, prior mental health diagnosis, psychotropic medication use	Overall study population: 41.9% likely GAD Living in an area recently impacted by the wildfires: **+** GAD symptoms	Not Assessed
Adu et al. [40] 2024 Exploring the Prevalence and Predictors of Low Resilience and Likely PTSD in Residents of Two Provinces in Canada During the 2023 Wildfires	Cross-sectional 298 Residents of Alberta and Nova Scotia who had subscribed to the Text4Hope mental health support services	Online survey During the wildfires, 2–3 months after their ignition in March 2023	PTSD, resilience	Participants reported whether they lived in an area recently affected by wildfires, had an evacuation order issued in their area of residence, had to evacuate from home due to wildfires, lost property to the wildfires, received support related to the wildfires, and their frequency of watching television images of wildfire destruction.	Brief Resilience Scale (BRS), Post Traumatic Stress Disorder Checklist Civilian (PCL-C)	Chi-squared/ Fisher’s exact tests	Not Assessed	Overall study population: 52.0% low resilience 39.3% likely PTSD All exposure measures: × low resilience, PTSD	Not Assessed
Mao et al. [41] 2024 Devastating Wildfires and Mental Health: Major Depressive Disorder Prevalence and Associated Factors among Residents in Alberta and Nova Scotia, Canada	Cross-sectional 298 Residents of Alberta and Nova Scotia who had subscribed to the Text4Hope mental health support services	Online survey During the wildfires, 2–3 months after their ignition in March 2023	Depression	Participants reported whether they lived in an area recently affected by wildfires, had an evacuation order issued in their area of residence, had to evacuate from home due to wildfires, lost property to the wildfires, received support related to the wildfires, and their frequency of watching television images of wildfire destruction.	Patient Health Questionnaire 9-item (PHQ-9)	Chi-squared/ Fisher’s exact tests	Not Assessed	Moderate to severe depression: 50.4% in total sample population 56.1% in those living in areas affected by wildfires All exposure measures: × moderate to severe depression	Not Assessed
**Maui Wildfire (2023)**									
Juarez et al. [42] 2025 Health and Social Support in the Aftermath of the Maui Wildfires	Cohort 1174 Maui residents who lived or worked within 10 miles of Lahaina, Kula, or any other area directly affected by the August 2023 wildfires	In-person 5–13 months	Anxiety, depression, self-esteem, suicidal ideation	Geospatial data were used to map wildfire perimeters and overlaid with participants’ geocoded addresses. A subset of participants provided answers on smoke, ash, and debris exposure across the timeframes of acute wildfire period (August 8-11, 2023), immediate post-fire period (August 12 - September 30, 2023), and 5-month intervals from October 2023 - September 2024.	CES-D, GAD-7, Rosenberg Self-Esteem Scale, self-report for suicidal ideation	Multivariable linear and logistic regression models	Age, sex, race and ethnicity, body mass index (BMI), socioeconomic status, preexisting lung conditions	Overall study population: 27.2% clinical anxiety symptoms 49.9% clinical depressive symptoms 26.1% low self-esteem 4.6% suicidal ideation Acute wildfire exposure: × anxiety **+** depression Inside fire perimeter: × anxiety, depression Immediate post-wildfire exposure: **+** anxiety × depression	Social support
**California Wildfires (2020)**									
Jung et al. [43] 2024 Fine Particulate Matter From 2020 California Wildfires and Mental Health–Related Emergency Department Visits	Time-series 86,609 mental health-related emergency department visits California residents who presented to a California emergency department from July - December 2020 for mental health conditions without COVID-19	All secondary data sources N/A	All-cause mental health conditions, as well as those specifically for psychoactive substance use, non-mood psychotic disorders, anxiety, depression, and other mood-affective disorders	Daily wildfire fine particulate matter (PM_2.5_) exposure, with up to 7-day lags, based on participants’ residential ZIP codes. These exposure estimates are derived from a machine learning model using PM_2.5_ data from ground station, satellite, and meteorological reanalysis sources to distinguish between wildfire PM_2.5_ and background PM_2.5_ [44].	ICD-10 codes for all-cause mental health disorders, psychoactive substance use disorders, non-mood psychotic disorders, other mood-affective disorders, depression, and anxiety	Quasi-Poisson distributed lag nonlinear model	Daily average temperature, daily average humidity, COVID-19-related emergency department counts, Social Deprivation Index score, baseline mental health emergency department counts, season, holidays, day of the week, and proximity to the active fire zone on a given date in a given ZIP code	A 10 µg/m^3^ increase in daily wildfire PM_2.5_: **+** all-cause mental health conditions, anxiety, depression, other mood-affective disorders	Age, sex, race, ethnicity
Sugg et al. [45] 2022 Understanding the Concurrent Risk of Mental Health and Dangerous Wildfire Events in the COVID-19 Pandemic	Two quasi-experimental studies 48,415 texts during non-wildfire exposure time and 3,430 texts during wildfire exposure time Users of the Crisis Text Line (CTL) with area code locations that were directly impacted by the wildfires	Near real-time scrubbed and anonymized data were from the CTL. At the end of each phone conversation, CTL users could take an optional survey on age, demographics, and other relevant identifiers. Pre-intervention: 227 days before the first fire ignited (August 17, 2020). Post-intervention: 60 days after the first fire ignited.	Crisis texts	Wildfire exposure was defined as an area code location directly impacted by wildfire (direct location of either the LNU Lightning Complex Fire, SCU Lightning Complex Fire, or the Bobcat fire). CTL conversations were assigned to wildfires using these area-code boundaries.	The CTL employs a complex algorithm to label text conversations with a crisis response tag (e.g., suicidal thoughts, self-harm, depression), which were included as mental health proxies in this analysis.	Interrupted time-series design with autoregressive integrated moving average (ARIMA) models; difference-in-differences analysis with generalized estimating equations	Age, gender, race	Timing of wildfire events and living in an area code location exposed to wildfires: × crisis texts	Not Assessed
**Black Summer Bushfires (2019–2020)**									
Sanatkar et al. [46] 2025 Emergency Department Mental Health presentations in Bushfire-, Flood-, Storm-, Drought-, and COVID-19-affected Areas: Analysis of Growth Models Between 2017 and 2021	Ecological The total number of emergency department visits is not specified. Adults residing in New South Wales regions with high, medium, and lesser exposure to fires, floods, storms, droughts, COVID-19 infections and pandemic-related layoffs	All secondary data sources N/A	Weekly emergency department visits for suicidal ideation, self-harm or mental health conditions from 2017–2021	A cumulative disaster exposure index was used to define the degree of regional exposure to disasters in 2020, using data on bushfire burns, droughts, floods or storms, COVID-19 infections, and pandemic-related job losses. Emergency department visit rates were compared across time periods: *Pre-bushfires* (01/01/2017–09/07/2019), *bushfires* (07/14/2019–02/26/2020), *COVID-19* (04/03/2020–09/17/2021), and the *post-period* (09/24/2021–12/24/2021).	ICD-10 codes	Negative binomial model with random intercepts for region	Age, sex	Compared to pre-period: Bushfire (all phases) in low and medium exposed regions: × emergency department visits Post-bushfire and COVID-19 period in low and medium exposed regions: **-** emergency department visits Bushfire (mid-phase) in high disaster exposed regions: **-** emergency department visits Bushfire (early and late phases) in high disaster exposed regions: × emergency department visits Post-bushfire and COVID-19 period in high disaster exposed regions: **-** emergency department visits	Not Assessed
Cruwys et al. [47] 2023 Social Group Connections Support Mental Health Following Wildfire	Cross-sectional national survey 627 Individuals ≥ 18 years old who lived in Australia since August 2019 and were directly affected by the fires	Large national survey 12–18 months	PTSD	Exposure was defined as high, medium, or low.^a^	Post-traumatic stress disorder–8 items (PTSD-8)	Descriptive statistics	Not Assessed	Low exposure: 9.7% PTSD Medium exposure: 29.5% PTSD High exposure: 58.7% PTSD and + PTSD	Not Assessed
Lykins et al. [48] 2023 Australian Youth Mental Health and Climate Change Concern After the Black Summer Bushfires	Cross-sectional survey 746 Young Australians in New South Wales between the ages of 16 and 25	Online survey questionnaire 0–3 months	Adjustment disorder, anxiety, climate change concern/distress, depression, resilience, stress, substance use	Participants were considered directly exposed if they responded “yes” to the question, “Have you been directly affected by any of the bushfires over the past year?”	Adjustment Disorder New Module–8 items (ADNM8), BRS, Depression Anxiety Stress Scale–21 items (DASS-21), UNCOPE Alcohol and Substance Abuse Screener	Analysis of variance (ANOVA) and bootstrapped Pearson’s correlations	Not Assessed	Direct exposure: **+** adjustment disorder symptoms, anxiety, climate change-related distress and concern, depression, stress, and substance use **-** resilience	Not Assessed
Rodney et al. [49] 2021 Physical and Mental Health Effects of Bushfire and Smoke in the Australian Capital Territory 2019-20	Cross-sectional survey 2084 Participants were aged at least 18 years or older, had to understand an online questionnaire in English, and had a residential address defined by specific postcode areas constituting the Australian Capital Territory (ACT) and immediately surrounding postcodes.	Online survey questionnaire 6 weeks	Self-reported mental health outcomes (i.e., anxiety, depression, sleep)	Direct fire exposure in the current season was measured as any, scaled, and cumulative exposure.^b^ Previous fire exposure was also assessed.	Self-rated health status, previous mental health diagnoses, and attribution of mental health symptoms to smoke	Chi-square tests and ordinal logistic regression	Background, demographic, and self-reported health variables	As a result of smoke: 45.3% anxiety symptoms 21.4% feeling depressed 37.2% disrupted or poor sleep Direct fire exposure (any, scale, cumulative): **+** self-reported negative mental health outcomes Previous fire exposure (any, scale, cumulative): × self-reported negative mental health outcomes	Not Assessed
**Oregon Wildfires (2018)**									
Mirabelli et al. [50] 2022 Wildfire Smoke and Symptoms Affecting Mental Health Among Adults in the U.S. State of Oregon	Cross-sectional survey 5807 Adults living in Oregon in 2018	Telephone survey (Behavioral Risk Factor Surveillance System [BRFSS]) Rolling data collection from January 2018 - February 2019	Anxiety symptoms, depressive symptoms	Daily, county-level estimates of smoke plume density from 2017 to 2019 from the US National Oceanic and Atmospheric Administration (NOAA) Hazard Mapping System were used as a proxy for exposure to wildfire smoke. The number of weeks of medium and heavy plume density were calculated in each county for the preceding 365 days and linked with each BRFSS respondent’s data by interview date and county of residence.	BRFSS anxiety and depression module	Weighted population estimates and logistic regression	Age, sex, educational attainment, employment status, and residence (metropolitan versus non-metropolitan)	Medium or heavy smoke for ≥ 6 weeks, compared to ≤ 4 weeks: 30.0% higher prevalence in beingunable to stop or control worrying more than half the time during the past two weeks Medium or heavy smoke: **+** little interest or pleasure in doing things; cannot stop or control worrying × feeling down, depressed, or hopeless; nervous, anxious, or on edge Heavy smoke: **+** nervous, anxious, or on edge × cannot stop or control worrying; feeling down depressed or hopeless; little interest or pleasure in doing things	Not Assessed
**Camp Fire (2018)**									
Silveira et al. [51] 2021 Chronic Mental Health Sequelae of Climate Change Extremes: A Case Study of the Deadliest Californian Wildfire	Cross-sectional survey 725 Participants were either: (a) students in the department of psychology at the California State University (CSU) in Chico; (b) individuals enrolled in the CSU Basic Needs program that provided disaster relief and community-based support directly to Camp Fire victims; or (c) individuals from the University of California, San Diego	Online questionnaire survey 6 months	GAD, major depressive disorder (MDD), PTSD	The Life Events Checklist (LEC-5) from the DSM-5 was used to assess fire exposure.	BRS, Childhood Trauma Questionnaire (CTQ), GAD-7, Mindful Attention Awareness Scale (MAAS), Patient-Reported Outcomes Measurement Information System (PROMIS)–Sleep Disturbance scale, PTSD Checklist for DSM-5 (PCL-5), PHQ-9	Multiple regression models	Demographic variables, age, sex, ethnicity, and socioeconomic status	Direct fire exposure: **+** GAD, MDD, PTSD	Interaction terms between fire exposure and vulnerability or resilience factors (childhood trauma, mindfulness, self-reported resiliency, and sleep disturbances) were not significant.
**Fort McMurray Wildfire (2016)**									
Obuobi-Donkor et al. [52] 2022 Prevalence and Correlates of Cannabis Abuse Among Residents in the Community of Fort McMurray, a City in Northern Alberta Which Had Endured Multiple Natural Disasters	Cross-sectional survey 186 Residents of Fort McMurray in 2021	Online survey questionnaire 5 years; data collected over 39 days (April - June 2021)	Substance use (i.e., cannabis abuse)	Self-reported exposure via survey to COVID-19 alone and to COVID-19, flooding, and wildfire traumas	Cannabis use was assessed through a self-reported question (Have you abused cannabis in the past year? ). BRS, GAD-7, PCL-C, and PHQ-9 were also employed but were not assessed in relation to wildfire exposure.	Chi-square test	Not Assessed	Overall study population: 14.0% self-reported cannabis use Exposure to COVID-19, flooding, and wildfire trauma: × cannabis abuse	Not Assessed
Mao et al. [53] 2022 Post-Traumatic Stress Disorder, Major Depressive Disorder, and Wildfires: A Fifth-Year Postdisaster Evaluation among Residents of Fort McMurray	Cross-sectional survey 186 Residents of Fort McMurray in 2021	Online survey questionnaire 5 years; data collected over 39 days (April - June 2021)	MDD, PTSD	Wildfire exposure-related variables were: Resided^c^ Property loss^d^ Witnessed burning^e^ Fearful^f^ Watch frequency^g^ Read frequency^h^	PCL-C, PHQ-9	Chi-square test and logistic regression analysis	Both MDD and PTSD models adjusted for history of mental health conditions before the fire, history of psychotropic drug use before the fire, receipt of counseling/support in the past year, and desire to receive mental health counseling. The MDD model also adjusted for unemployment status.	Overall study population: 45.0% MDD 39.6% PTSD All wildfire exposure-related predictors: × MDD, PTSD	Not Assessed
Agyapong et al. [54] 2022 Cumulative Trauma From Multiple Natural Disasters Increases Mental Health Burden on Residents of Fort McMurray	Cross-sectional survey 186 Residents of Fort McMurray in 2021	Online survey questionnaire 5 years; data collected over 39 days (April - June 2021)	GAD, MDD, PTSD, resilience	Multi-trauma exposure: wildfire, flooding, and COVID-19. Wildfire trauma was defined using a question related to being fearful for one’s own life or the lives of family and friends during the wildfire evacuation.	BRS, GAD-7, PCL-C, PHQ-9	Chi-square analysis, logistic regression analyses, and one-way ANOVA	Background, demographic, and clinical variables	For those with multi-trauma exposure, compared to those with COVID-19 only trauma: + GAD × low resilience (trending towards significance), MDD, PTSD (trending towards significance)	Not Assessed
Adu et al. [55] 2022 Five Years After the Fort McMurray Wildfire: Prevalence and Correlates of Low Resilience	Cross-sectional study 186 Residents of Fort McMurray in 2021	Online survey questionnaire 5 years; data collected over 39 days (April - June 2021)	Resilience	Wildfire exposure-related variables were: Resided^c^ Property loss^d^ Witnessed burning^e^ Fearful^f^ Watch frequency^g^ Read frequency^h^ Volume of damaged properties^i^ Living situation^j^	BRS	Chi-square/Fisher’s exact tests and logistic regression analysis	Background, demographic, clinical, mental health, and social support variables	Overall study population: 37.4% low resilience All wildfire exposure-related predictors: × resilience	Not Assessed
Ritchie et al. [56] 2021 Long-Term Mental Health Effects of a Devastating Wildfire Are Amplified by Sociodemographic and Clinical Antecedents in College Students	Cross-sectional survey 329 Students enrolled in Keyano College in November 2017	Information leaflets and survey forms were distributed across campus and in lecture rooms. 18 months	GAD, MDD, PTSD	Wildfire exposure-related variables were: Area of residence relative to destroyed properties Witnessed burning^f^ Fearful^g^ Volume of damaged properties^i^ Home completely destroyed^k^	GAD-7, PHQ-9, PCL-5	Chi-square tests	Not Assessed	Overall study population: 23.4% MDD 18.7% GAD 11.0% PTSD Area of residence relative to destroyed properties: **+** PTSD × GAD, MDD Witnessed burning of homes, home completely destroyed by fire, and fearful for life or the lives of friends/family: × GAD, MDD, PTSD	Not Assessed
Verstraeten et al. [57] 2021 Maternal Mental Health After a Wildfire: Effects of Social Support in the Fort McMurray Wood Buffalo Study	Cross-sectional survey 200 English-speaking women who were evacuated from the Fort McMurray wildfire during or shortly before pregnancy	Online self-report questionnaire Timing of data collection not specified (recruitment ran from November 7, 2016 – October 29, 2018)	PTSD-like symptoms	The timing of exposure to the fire preconception (up to 6-months before) and during pregnancy was defined as the number of days between the fire ignition and the best estimated due date.	Impact of Events Scale-Revised (IES-R)	Multiple linear regression	Time between the fire and recruitment, timing of fire exposure before or during pregnancy, whether delivery had occurred before recruitment, and parity	Time since the fire: × PTSD-like symptoms	Not Assessed
Pazderka et al. [58] 2021 Collective Trauma and Mental Health in Adolescents: A Retrospective Cohort Study of the Effects of Retraumatization	Panel study 1035 Students enrolled in either junior or senior high schools in both Public and Catholic School Districts in Fort McMurray in 2017	An initial intake survey, with mental health surveys for 3 consecutive years were administered in-person, during class time. These data are from the third round of data collection (approximately 3.5 years post-fire)	Anxiety, collective trauma, depression, PTSD, suicidality	Students were categorized into a “wildfire group” if they indicated that the wildfire was the worst trauma they had experienced, or a “prior trauma group” if they indicated that they had experienced a worse trauma prior to the wildfire.	Child PTSD Symptom Scale (CPSS), Hospital Anxiety and Depression Scale (HADS), Patient Health Questionnaire for Adolescents (PHQ-A)	A two-tailed, independent-samples t-test was conducted to compare the mental health outcomes in the prior trauma group with the wildfire group.	Age, sex	Comparing the wildfire group to the prior trauma group: **-** depression, lifetime suicide risk, PTSD × anxiety	Trauma type
Belleville et al. [59] 2021 Psychological Symptoms Among Evacuees From the 2016 Fort McMurray Wildfires: A Population-Based Survey One Year Later	Cross-sectional survey 1510 Current or former Fort McMurray residents who were evacuated from their homes during the wildfire	Telephone survey 1 year; data collected over 80 days (May - July 2017)	Depression, GAD, insomnia, PTSD, substance use	Degree of exposure was assessed with six yes/no questions on whether participants smelled smoke or fire, saw buildings or surroundings on fire, feared for the safety of a loved one, saw explosions or buildings collapsing, feared for their own safety, or was separated from a loved one.	CAGE (Cut down, Annoyed, Guilty, and Eye-opener) Substance Abuse Screening Tool (CAGE), GAD-7, Insomnia Severity Index (ISI), PCL-5, PHQ-9	Generalized linear models	Not Assessed	Overall study population: 28.5% insomnia 15.4% PTSD 15.0% MDD 14.2% GAD 7.9% substance use disorder Subjective level of fear during the fire: **+** anxiety, PTSD × depression, insomnia, substance use Smelled smoke or fire: × anxiety, depression, insomnia, PTSD, substance use Saw buildings/surroundings on fire: × anxiety, depression, insomnia, PTSD, substance use Feared for the safety of a loved one: **+** PTSD × anxiety, depression, insomnia, substance use Saw explosions or buildings collapsing: **+** depression, insomnia, PTSD × anxiety, substance use Feared for their own safety: × anxiety, depression, insomnia, PTSD, substance use Separated from a loved one: + insomnia × anxiety, depression, PTSD, substance use	Not Assessed
Agyapong et al. [60] 2020 Long-Term Mental Health Effects of a Devastating Wildfire Are Amplified by Socio-Demographic and Clinical Antecedents in Elementary and High School Staff	Cross-sectional survey 197 Staff of the Fort McMurray School Districts who were employed 18 months after the wildfire	Online questionnaire survey 18 months	GAD, MDD, PTSD	Wildfire exposure-related variables were: Witnessed burning^e^ Fearful^f^ Watch frequency^g^ Read frequency^h^ Volume of damaged properties^i^ Home substantially destroyed^l^ Business substantially destroyed^m^	GAD-7, PCL-5, PHQ-9	Univariate analyses with Chi-square tests	Not Assessed	Overall study population: 18.3% MDD 15.7% GAD 10.2% PTSD Volume of damaged properties: × GAD, MDD, PTSD Witnessed burning: **+** PTSD × GAD (trending towards significance, *p* = 0.06), MDD Fearful: **+** GAD, MDD, PTSD Home substantially destroyed: × GAD, MDD, PTSD Business substantially destroyed: × GAD, MDD, PTSD Read frequency: × GAD, MDD, PTSD Watch frequency: × GAD, MDD, PTSD	Not Assessed
Moosavi et al. [61] 2019 Mental Health Effects in Primary Care Patients 18 Months After a Major Wildfire in Fort McMurray: Risk Increased by Social Demographic Issues, Clinical Antecedents, and Degree of Fire Exposure	Cross-sectional survey 290 Patients attending the only out-of-hours primary care clinic in Fort McMurray 18 months following the wildfire	Self-administered paper-based questionnaires 18 months	GAD, MDD, PTSD	Wildfire exposure-related variables were: Witnessed burning^e^ Fearful^f^ Watch frequency^g^ Read frequency^h^ Volume of damaged properties^i^ Home substantially destroyed^l^	GAD-7, PCL-5, PHQ-9	Univariate analyses with chi-square tests and logistic regression	Age, employment status, history of mental health conditions before the fire, received counseling/support after the fire	Overall study population: 24.8% MDD 18.0% GAD 13.6% PTSD Volume of damaged properties: × GAD, MDD, PTSD Witnessed burning: + GAD, MDD, PTSD (trending towards significance, *p* = 0.06) Fearful: + GAD, MDD × PTSD Home completely destroyed: × GAD, MDD, PTSD Watch frequency: × GAD, MDD, PTSD Read frequency: × GAD, MDD, PTSD	Not Assessed
Brown et al. [62] 2019 Significant PTSD and Other Mental Health Effects Present 18 Months After the Fort Mcmurray Wildfire: Findings From 3,070 Grades 7–12 Students	Cross-sectional survey 3,070 Students in grades 7–12 who were enrolled in the Fort McMurray Public and Catholic Schools eighteen months after the wildfire	School-administered in-person survey 18 months	Anxiety, depression, PTSD, quality of life, resilience, self-esteem, substance abuse, suicidal thinking	The Impact of Fire Questionnaire (IOF)^n^ was administered. Participants were then defined into subgroups: (1) no impact of fire versus any impact of fire; (2) present during the fire versus not present; (3) saw the fire in person versus did not see it; and (4) home destroyed by the fire versus home not destroyed.	CPSS, CRAFFT (Car, Relax, Alone, Forget, Friends, Trouble) Screening Tool for Adolescent Substance Abuse (CRAFFT), Child and Youth Resilience Measure (CYRM-12), HADS, Kidscreen Questionnaire, PHQ-A, Rosenberg Self-Esteem Scale	Permutation testing	Not Assessed	Overall study population: 37.0% PTSD 31.0% depression 27.0% anxiety 15.0% alcohol or substance use disorder No impact of fire versus any impact: × anxiety, depression, PTSD, quality of life, self-esteem, substance use, suicidal thinking - resilience Present during the fire versus were not: × anxiety, depression, PTSD, quality of life, self-esteem, substance use, suicidal thinking + resilience Personally witnessed the fire versus did not: + anxiety, depression, PTSD, substance use, suicidal thinking - quality of life × resilience, self-esteem Homes were destroyed by the fire versus were not: + anxiety, depression, PTSD, substance use, suicidal thinking - quality of life, resilience, self-esteem	Not Assessed
Agyapong et al. [63] 2019 Prevalence Rates and Correlates of Likely Post-Traumatic Stress Disorder in Residents of Fort McMurray 6 Months After a Wildfire	Cross-sectional survey 486 Residents of Fort McMurray who were recruited in-person at locations across the city	Survey forms were completed at designated data collection points or were completed at home and returned within a week to the collection points. 6 months	PTSD	Wildfire exposure-related variables were: Witnessed burning^e^ Fearful^f^ Watch frequency^g^ Read frequency^h^ Volume of damaged properties^i^ Home completely destroyed^k^ Smoke damage^o^ No loss^p^ Post-fire residence^q^	PCL-5	Univariate analysis with the chi-square tests and logistic regression	Employment status, gender, history of mental health conditions before the fire, history of psychotropic drug use before the fire, received counseling/support after the fire	Overall study population: 12.8% PTSD Post-fire residence: + PTSD Read frequency: × PTSD Smoke damage: × PTSD No loss: × PTSD	Not Assessed
Brown et al. [64] 2019 After the Fort McMurray Wildfire There are Significant Increases in Mental Health Symptoms in Grade 7–12 Students Compared to Controls	Cross-sectional study 3,070 students from Fort McMurray compared to 2,796 students in Red Deer Students in grades 7–12 who were enrolled in the Fort McMurray Public and Catholic Schools in 2017, and students in grades 7–12 who were enrolled in Red Deer public schools in 2014	Both school districts administered the in-person survey during school hours. Data from Fort McMurray students were collected 18 months post-fire. Data from Red Deer students were collected in 2014.	Anxiety, depression, quality of life, self-esteem, substance abuse, suicidal thinking	Fort McMurray students were considered exposed and the Red Deer students were considered unexposed	CPSS (Fort McMurray only), CRAFFT, HADS, Kidscreen Questionnaire, PHQ-A, Rosenberg Self-Esteem Scale	Permutation testing	Not Assessed	Comparing exposed (Fort McMurray) students to unexposed (Red Deer) students: 31.0% versus 17.0% depression 16.0% versus 4.0% suicidal thinking 15.0% versus 16.0% anxiety disorder 13.0% versus 10.0% tobacco use + anxiety, depression, suicidal thinking, tobacco use - quality of life, self-esteem × alcohol or drug use	Not Assessed
Agyapong et al. [65] 2018 Prevalence Rates and Predictors of Generalized Anxiety Disorder Symptoms in Residents of Fort McMurray Six Months After a Wildfire	Cross-sectional survey 486 Residents of Fort McMurray who were randomly recruited in-person at locations across the city	Survey forms were completed at designated data collection points or were completed at home and returned within a week to the collection points. 6 months	GAD	Wildfire exposure-related variables were: Witnessed burning^e^ Fearful^f^ Watch frequency^g^ Read frequency^h^ Volume of damaged properties^i^ Home completely destroyed^k^ Smoke damage^o^ No loss^p^ Post-fire residence^q^	GAD-7	Univariate analysis with the chi-square tests and logistic regression	Age, employment status, gender, history of mental health conditions before the fire, history of psychotropic drug use before the fire, received counseling/support after the fire	Overall study population: 19.8% GAD Witnessed burning: + GAD Post-fire residence: + GAD Volume of damaged properties: × GAD Fearful: × GAD Read frequency: × GAD	Not Assessed
Cherry and Haynes [66] 2017 Effects of the Fort McMurray Wildfires on the Health of Evacuated Workers: Follow-up of 2 Cohorts	Cohort study 129 Workers based in Fort McMurray who had been recruited before the fire for two occupational health cohort studies	Online/phone survey questionnaire 3–26 weeks post-evacuation	Anxiety, depression, substance use	Exposure was defined by the events at the time of the fire and events since the fire.^s^	HADS	Multivariable regression models	Sex, length of time to questionnaire completion, health problem after the fire	Overall study population: 16.7% moderate or severe anxiety or depression Evacuated versus were not: + anxiety, depression × substance use Neighborhood damage and financial loss from work versus without: × anxiety, depression	Not Assessed
**Royal Gorge and Black Forest Wildfires (2013)**									
Scales et al. [67] 2025 Violent Deaths Following Disasters: A Retrospective Analysis	Ecological Only the Colorado-specific analysis and results are presented here, as that was the only wildfire disaster considered. This study population includes Colorado counties that were and were not affected by the Royal Gorge and Black Forest wildfires. A total of 2,322 deaths were recorded in the study period for all states, but the Colorado-specific deaths are not specified.	All secondary data sources N/A	Suicide deaths	The pre-period was the three months directly preceding the recorded wildfire start date according to the federal disaster declaration. The wildfire period covered the three calendar months following the start date. Counties that were eligible for public assistance that the US Federal Emergency Management Agency were considered affected while counties in the same state that were ineligible for public assistance were considered unaffected.	Using ICD-10 codes for intentional self-harm or sequelae of intentional self-harm, suicide deaths were distinguished from all violent deaths recorded in the US National Violent Death Reporting System.	Poisson regression	Not Assessed	Comparing wildfire period with the pre-period: × suicide deaths in affected counties × suicide deaths in unaffected counties	Not Assessed
**Wallow Fire (2011)**									
Eisenman et al. [68] 2015 An Ecosystems and Vulnerable Populations Perspective on Solastalgia and Psychological Distress After a Wildfire	Cross-sectional survey 416 Households in the communities surrounding the fire	Mailed census survey 13–14 months	Psychological distress, solastalgia	While all participants resided in communities affected by the fire, measured fire exposure-related variables were actively defending homes during the fire and financial impact of the fire on households.	Kessler Psychological Distress Scale 10-item (K-10), Solastalgia scale (adapted from Higginbottham et al. [69])	Multivariate logistic regression	Demographic, resource, and relative risk (i.e., permanent residence versus seasonal residence) variables	Overall study population: 35.0% moderate to high risk for psychological distress Higher solastalgia score and financial impact from fire: + psychological distress	Not Assessed
**Black Saturday Bushfires (2009)**									
Pacella et al. [70] 2024 Trajectory of Adjustment Difficulties Following Disaster: 10-year Longitudinal Cohort Study	Cohort 802 participants at Wave 1, 596 at Wave 2, and 436 at Wave 3 Participants from areas with self-reported moderate and high levels of fire-affectedness	Online or telephone-based surveys Wave 1: 3–4 years Wave 2: 5 years Wave 3: 10 years	Adjustment disorder, depression, fire-related PTSD	Participants self-reported their property loss, fear for life in fire, and bereavement in the bushfires.	Kessler Psychological Distress Scale 6-item (K-6), Posttraumatic Stress Disorder Checklist 4-item (PCL-4), PHQ-9	Multinomial logistic regression	Highest level of education, gender and age at the time of the bushfires	Overall study population: Wave 1: 15.7% adjustment disorder 12.1% depression 13.7% fire-related PTSD Wave 2: 14.8% adjustment disorder 9.6% depression 9.7% fire-related PTSD Wave 3: 18.6% adjustment disorder 8.9% depression 6.4% fire-related PTSD Property loss, feared for life in fire, bereaved in fire: ✖ probable adjustment disorder at Wave 1	Not Assessed
Cowlishaw et al. [71] 2021 Anger Dimensions and Mental Health Following a Disaster: Distribution and Implications After a Major Bushfire	Cross-sectional survey delivered in two waves 736 Participants were residents of 25 rural and regional communities across 10 locations in Victoria, Australia who participated in the second wave of the Beyond Bushfires study.	Online/phone survey 5 years; data collected over 4 months (July - November 2014)	Anger	The levels of bushfire affectedness were defined by evidence of high impact (i.e., multiple fatalities and significant property loss), medium impact (i.e., significant property damage and up to two fatalities), and low impact (i.e., no evidence of burning).	Dimensions of Anger Reactions Scale-5 (DAR-5)	Logistic regression models	Not Assessed	High bushfire affectedness: 10.0% significant anger problems + anger	Not Assessed
Bryant et al. [72] 2021 The Dynamic Course of Psychological Outcomes Following the Victorian Black Saturday Bushfires	Cohort 1,017 (Wave 1), 735 (Wave 2), and 525 (Wave 3) Adults aged at least 18 years of age living in 25 communities in 10 locations in Victoria	Online/phone survey for Wave1; later retested for Waves 2 and 3 Wave 1: 3–4 years Wave 2: 5 years Wave 3: 10 years	Development of heavy alcohol use, depression, fire-related PTSD, severe distress, PTSD, resilience	Participants self-reported their property loss, fear for life in fire, and bereavement in the bushfires.	Alcohol Use Disorder Identification Test–Consumption (AUDIT-C), K-6, PTSD Checklist-Specific (PCL-S), PHQ-9	Logistic regressions	Age, sex, highest level of education, recent major life stressors	Comparing Wave 3 to Wave 1 prevalences: 8.3% versus 10.9% depression 6.2% versus 12.2% fire-related PTSD 14.9% versus 18.7% general PTSD 18.5% versus 21.8% problem alcohol use 82.4% versus 77.8% resilience 4.4% versus 7.5% severe distress Worsened Outcomes (present at Wave 3, but not at Wave 1): *Worse property loss*: + depression, fire-related PTSD *Greater fear for one’s life during the fires*: + general PTSD Improved Outcomes (present at Wave 1, but not at Wave 3): *Less property loss*: + depression, resilience, severe distress Maintained Outcomes (present or absent at both waves) *More property loss*: + depression, severe distress	Not Assessed
Molyneaux et al. [73] 2019 Interpersonal Violence and Mental Health Outcomes Following Disaster	Cross-sectional 1016 Adults aged at least 18 years of age living in 25 communities in 10 locations in Victoria	Online/phone survey 3–4 years	Alcohol use, depression, PTSD	At the community-level: affectedness was defined as high (numerous fatalities and extreme physical destruction) or medium (sporadic fatalities and extensive property damage), with comparison communities (those with bushfire risk but only sporadic property damage).	AUDIT-C, PCL, PHQ-9	Multiple regression	Gender, age at the time of the fires, highest level of education, and individual bushfire exposure	Comparing women in high bushfire-affected communities to those in low or medium bushfire-affected communities: + experience of assault or violence post-fire Experiences of violence among women: + depression, PTSD - alcohol use	Gender
Gallagher et al. [74] 2019 The Effect of Group Involvement on Post-disaster Mental Health: A Longitudinal Multilevel Analysis	Cohort 642 (individual-level analysis) 552 (community-level analysis) Subsamples of adults living in the same affected community in Victoria during the bushfire and at both data collection timepoints	Online/phone survey 3 and 5 years (two data collection timepoints)	Depression, PTSD	At the community-level: group involvement was defined as participants’ sum total of current group memberships (up to 10 per participant) At the individual level: participants reported property and interpersonal loss from the bushfires.	PCL-C, PHQ-9	Individual-level longitudinal regression and multilevel logistic regression	Community-level model: area-level socioeconomic status Individual-level model: age, sex, education, employment, household composition, major life events, group involvement variables	Comparing 5th year to 3rd year post-fire: - probable PTSD (14.2% versus 18.1%) - probable major depression (8.1% versus 10.7%) Community-level: *Moderate group involvement*: - PTSD × depression Individual-level: *Property loss*: + depression (3rd year) × depression (5th year) + PTSD (3rd and 5th year) *Interpersonal loss*: + depression, PTSD (3rd year) × depression, PTSD (5th year)	Not Assessed
Bryant et al. [75] 2018 Longitudinal Study of Changing Psychological Outcomes Following the Victorian Black Saturday Bushfires	Cohort study 1,017 (Wave 1) and 735 (Wave 2) Adults aged at least 18 years of age living in 25 communities in 10 locations in Victoria	Online/phone survey for wave 1; later retested for wave 2 Wave 1: 3–4 years Wave 2: 5 years	Development of heavy alcohol use, depression, fire-related PTSD, severe distress, PTSD, resilience	Participants self-reported their property loss, fear for life in fire, and bereavement in the bushfires.	AUDIT-C, K-6, PCL-S, PHQ-9	Logistic regressions	Age, sex, highest level of education, recent major life stressors	Comparing Wave 2 to Wave 1 prevalences: 8.7% versus 12.1% fire-related PTSD 14.7% versus 18.2% general PTSD 9.0% versus 10.9% major depressive episode 81.8% versus 77.1% resilience 21.4% versus 22.1% problem alcohol use LATE ONSET (present at Wave 2, but not at Wave 1) *Property loss*: + fire-related PTSD, PTSD × heavy drinking, depression, resilience, severe distress *Feared for life or anyone close had died in the fires*: × heavy drinking, depression, fire-related PTSD, PTSD, resilience	Not Assessed
Bryant et al. [76] 2017 Mental Health and Social Networks After Disaster	Cohort 558 Adults aged at least 18 years of age living in 25 communities in 10 locations in Victoria	Online/phone survey 2.75-5 years	Depression, PTSD	Participants self-reported their property loss, fear for life in fire, and bereavement of someone close in the bushfires.	PCL-C, PHQ-9	Logistic regression	Not Assessed	Overall study population: 38.5% depression 15.6% PTSD Fear for life in fire: + depression, PTSD Loss of someone close in fire: + depression, PTSD Property damage: + depression, PTSD	Not Assessed
Gallagher et al. [77] 2017 Dyadic Effects of Attachment on Mental Health: Couples in a Postdisaster Context	Cohort 127 heterosexual couples Subsample selected from a wider sample of residents from 25 communities in 10 locations in rural and regional Victoria	Online/phone survey 3–4 years	Depression, PTSD	Exposure to the bushfires was measured in terms of fear for life, interpersonal loss, and property loss.	PCL-C, Patient Health Questionnaire 8-item (PHQ-8)	Actor–partner interdependence model based on structural equation modeling	Age, tertiary education, children living in the household, and the experience of another traumatic event, attachment anxiety, attachment avoidance	Shared disaster exposure among couples: + depression, PTSD	Gender
Gallagher et al. [78] Mental Health Following Separation in a Disaster: The Role of Attachment 2016	Cohort 914 Adults aged at least 18 years of age living in 24 communities in 10 locations in Victoria	Online/phone survey 3–4 years	Depression, PTSD	Participants self-reported whether they were separated from close family members during the bushfire.	PCL, PHQ-9	Multigroup structural equation modeling	Attachment anxiety and avoidance	Comparing those who were separated from close family members during the bushfire to those who were not: × depression **+** PTSD	Not Assessed
Forbes et al. [79] 2015 The Role of Anger and Ongoing Stressors in Mental Health Following a Natural Disaster	Cohort 1,017 Adults aged at least 18 years of age living in 25 communities in 10 locations in Victoria	Survey (format not specified) 3–4 years	Depression, PTSD	A composite measure "exposure to bushfire"was assessed in terms of binary fear for life, binary death of loved ones, a continuous measure of property loss, and regional bushfire impact (high, medium, or low).	PCL (fire-related and general), PHQ-9 A composite "mental health outcomes" was calculated with the PCL and PHQ-9 scores.	Structural equation modeling	Not Assessed	Exposure to bushfire: + composite "mental health outcomes" Anger (measured with the Anger Attacks Questionnaire) significantly mediated this relationship.	Gender differences were not significant.
Bryant et al. [80] 2014 Psychological Outcomes Following the Victorian Black Saturday Bushfires	Cross-sectional survey (baseline assessment of a cohort) 1,017 Adults aged at least 18 years of age living in 25 communities in 10 locations in Victoria	Online/phone survey 3–4 years	Alcohol use, depression, psychological distress, PTSD, resilience	Community affectedness was defined as high (many houses lost plus fatalities), medium (small number of fatalities or no fatalities but significant amount of property damage), and low (no evidence of burning).	AUDIT-C, K-6, PCL-S, PHQ-9	Logistic regression	Age, highest level of education, major life stressors, sex	Comparing high to medium to low-fire affectedness: 15.6% versus 7.2% versus 1.0% PTSD 12.9% versus 8.8% versus 6.3% depression 9.8% versus 5.0% versus 4.9% severe psychological distress 4.7% versus 18.7% versus 19.6% heavy drinking High-fire affectedness: + alcohol use, depression, psychological distress, PTSD - resilience	Sex
**Gap, Tea and Jesusita Fires (2008–2009)**									
Felix et al. [81] 2015 Family Functioning and Posttraumatic Growth Among Parents and Youth Following Wildfire Disasters	Cross-sectional survey 100 (50 parent-youth dyads) Wildfire survivors who had been evacuated	In-home interview and survey questionnaire 1 year after the last fire (May 2009)	Post traumatic growth	Perceived fire-related stress was measured by a scale the research team developed consisting of 9 items rated on a 4-point Likert Scale.	Cognitive Emotion Regulation Questionnaire Short Form (CERQ-S), Mental Health Inventory (MHI-5), Post-Traumatic Growth Inventory-Short Form (PTGI-SF), Protective Factors Survey (PFS)	Hierarchical linear regressions	Demographic, recovery environment, current mental health, and coping variables	Greater fire stress: + post traumatic growth	Not Assessed
Afifi et al. [82] 2012 The Impact of Uncertainty and Communal Coping on Mental Health Following Natural Disasters	Cross-sectional study 337 Residents of Santa Barbara, California and surrounding communities	Telephone survey questionnaire 5 months after the last fire (May 2009)	Communal coping, psychological distress, stress, uncertainty	Respondents were classified by evacuation status (i.e., evacuated versus not evacuated).	Communal coping measure adapted from Afifi et al. (2006) [83], MHI-5, uncertainty measurement adapted from Afifi and Weiner [84]	Hierarchical regression analysis	Age, sex (in the regression of uncertainty and mental health)	Comparing those who were evacuated to those who were not: + stress, reliance on communal coping, uncertainty about the safety of their home - uncertainty about the safety of close others	Not Assessed
**Witch Creek and Guejito Fires (2007)**									
Tally et al. [85] 2013 The Impact of the San Diego Wildfires on a General Mental Health Population Residing in Evacuation Areas	Cross-sectional survey 754 San Diego County Mental Health system clients	Public mental health clinics’ satisfaction surveys with an appended supplemental section designed to assess the impact of the fire 0–2 weeks	Depression/sadness, stress/anxiety/fear, pre-existing conditions (i.e., bipolar disorder, MDD, schizophrenia/schizoaffective disorder)	Respondents were classified by evacuation status: (1) not in an evacuation area, (2) in a designated evacuation area but did not evacuate, and (3) in an evacuation area who evacuated their residence.	A questionnaire assessed the impact of the fires on mental health status (i.e., depression/sadness caused by fires, stress/anxiety/fear caused by fires).	Multivariate Analysis of Covariance (MANCOVA)	Not Assessed	Having to evacuate: + depression/sadness caused by the fire, stress/anxiety/fear	Not Assessed
**Spain Forest Fires (2006)**									
Caamano-Isorna et al. [86] 2011 Respiratory and Mental Health Effects of Wildfires: An Ecological Study in Galician Municipalities (North-west Spain)	Ecological study 4,212 (municipality-months) Galician provinces of Corunna and Pontevedra (north-west Spain)	Wildfires that occurred in August 2006 were assigned to the respective municipalities. Time of data collection was not specified, but the study was conducted at least one year after the wildfires, as the research team calculated the defined daily doses of anxiolytics-hypnotics or drugs for obstructive airway diseases (DOADs) per 1000 inhabitants per day (DDDs) for the 12-month periods pre- and post-August 2006.	Anxiety-related substance use (i.e., consumption of anxiolytics-hypnotics and DOADs)	Municipalities’ exposure to wildfires was classified into three categories: no exposure (0 to 3 wildfires); medium exposure (4 to 10 wildfires); and high exposure (more than 10 wildfires).	DDDs	Additive models for time series	Not Assessed	Medium and high exposure: + anxiolytics-hypnotics and DOADs consumption	Pension status, sex
**Greek Forest Fires (2007)**									
Papadatou et al. [87] 2012 Adolescents’ Reactions After a Wildfire Disaster in Greece	Cross-sectional 1,468 Adolescents in two municipalities whose secondary school principals indicated that at least some of their students had been affected by the fires	In-person survey administered during a school day 6 months	Depression, PTSD	Wildfire Experience Questionnaire used to identify proximity to wildfire, objective threat level, perceived life threat, and post-disaster losses due to the wildfire	Children’s Revised Impact of Event Scale (CRIES-13), Depression Self-Rating Scale (DSRS)	Multilevel Poisson regression	Age, gender, pre-disaster life events, perceived social support, and escape-oriented and control-oriented coping strategies	Overall study population: 29.4% PTSD 20.0% depression Objective and perceived threat to self and others during the fires: + PTSD × depression	Not Assessed
Papanikolaou et al. [88] 2011 Surveying the Ashes: Experience from the 2007 Peloponnese Wildfires Six Months after the Disaster	Cross-sectional 800 Adult residents from disaster affected villages and demographically similar residents from neighboring, unaffected villages	In-person survey 6 months	Anxiety, depression, paranoia, psychological distress	Participants were considered exposed if they were residents of villages declared affected by the fires via a Ministerial Decree.	Symptom Checklist-90-Revised (SCL-90-R)	ANOVA	Not Assessed	Comparing fire exposed to non-exposed: + anxiety, depression, paranoia, psychological distress (overall)	Not Assessed
Papanikolaou et al. [89] 2011 Psychological Distress Following Wildfires Disaster in a Rural part of Greece: A Case-control Population-based Study	Cross-sectional survey 615 (353 “cases” (i.e., exposed to the fire) and 262 controls) “Cases”: residents aged 18–65 years who lived in the five prefectures characterized as disaster areas by the Hellenic Republic Ministry of Interior Controls: residents from nearby, unaffected areas	Face-to-face interviews 6 months	Anxiety, depression, paranoia, psychological distress	Exposed individuals were identified by their location in or close proximity to the disaster areas. Non-exposed individuals were matched to those exposed on gender, age, education, marital status, and regional distribution. The number and types of losses from the fire were assessed.	SCL-90-R	Multivariate general linear model	Age, education, gender, marital status	Those exposed to the disaster: + anxiety, depression, paranoia, psychological distress Those who had lost a close relative: + paranoia Those with personal injury or the injury of a close relative: × psychological distress	Not Assessed
Mellon et al. [90] 2009 Locus of Control and Psychopathology in Relation to Levels of Trauma and Loss: Self-Reports of Peloponnesian Wildfire Survivors	Cross-sectional survey 800 Adult residents from disaster affected areas and directly adjoining areas with no fire damage, closely matched on gender, age, educational, marital and regional distributions	In-person survey 6 months	Distress/ psychological dysfunction, PTSD	Participants were considered exposed if they were residents of the prefectures designated by the Hellenic Republic Ministry of Interior to contain the fire disaster areas.	SCL-90-R, SCL-90-R PTSD subscale	ANOVA	Not Assessed	Comparing fire exposed to non-exposed: + distress/ psychological dysfunction, PTSD	Not Assessed
**Eyre Peninsula Bushfire (2005)**									
Yelland et al. [91] 2010 Bushfire Impact on Youth	Cross-sectional 155 Youth from two schools in the Lower Eyre Peninsula that were directly affected by the bushfire	Online survey 11–15 months	PTSD	Students self-reported their experience of actual life-threatening events, perceived life threat, loss and life disruption immediately post-fire, and ongoing loss/life disruption 13–15 months post-fire.	Posttraumatic Stress Disorder Reaction Index for Children-Revised (PTSD-RI-R)	Stepwise regression	Age, gender, school	Overall study population: 17.0% moderate PTSD 10.0% very severe to severe PTSD Perceived life threat, ongoing loss/disruption: + PTSD symptom severity Including actual life-threatening events and immediate loss/disruption did not significantly increase the model’s explained variance.	Not Assessed
**Southern California Wildfires (2003)**									
Scher and Ellwanger [92] 2009 Fire-related Cognitions Moderate the Impact of Risk Factors on Adjustment Following Wildfire Disaster	Cohort 200 Students attending California State University, San Bernardino, which was directly affected by the 2003 southern California wildfires	In-person and mailed surveys Acute period: 14–31 days (after fires that affected the university were contained) Follow-up: 7 months	Anxiety, depression	A Fire Impact Questionnaire was developed to assess direct and indirect impact of wildfires on the respondent, with higher scores indicating greater impact.	Beck Anxiety Inventory (BAI), Beck Depression Inventory-II (BDI-II)	Hierarchical regression	Gender, ethnic/racial minority status	High fire impact when negative fire-related cognitions were high: + anxiety × depression Among evacuators: similar but slightly stronger associations as in main analysis	Fire-related cognitions (measured with the Posttraumatic Cognitions Inventory [PTCI])
Marshall et al. [93] 2007 Psychiatric Disorders Among Adults Seeking Emergency Disaster Assistance After a Wildland-Urban Interface Fire	Cohort Baseline: 357 Follow-up: 234 Adult evacuees from the 2003 Southern California firestorms who sought emergency disaster services from American Red Cross and government relief centers	In-person survey Baseline: within days of evacuation Follow-up: 3 months	Major depression, PTSD	Participants self-reported household damage or destruction, seeing flames from home or neighborhood, difficulty breathing from smoke/ash, physical injury from the fire, fearing for life, fearing for a loved one’s life, fearing that home/property was in danger, feeling helpless, feeling terrified, and being separated from loved ones during the fire.	PCL, PHQ-8	Logistic and best-subset regressions	Age, gender, race or ethnicity, education, employment status, income	Overall study population at follow-up: 33% probable major depression 24% probable PTSD Property damage, physical injury from the fire: + psychopathology (either depression or PTSD) at follow-up No other exposure measures improved the model’s predictive performance.	Not Assessed
**British Columbia Forest Fires (2003)**									
Moore et al. [94] 2006 Population Health Effects of Air Quality Changes Due to Forest Fires in British Columbia in 2003	Ecological Not specified Physician billing visits for mental health conditions in the Kelowna and Kamloops regions of British Columbia	All secondary data sources N/A	Mental health conditions	Exposure was defined by wildfire ignition date with the unexposed period from 1993–2002.	International Classification of Diseases, Ninth Revision (ICD-9) codes	Z-test to compare differences during exposed and unexposed periods	Not Assessed	Comparing weekly physician rates during the fires to the historical weekly rates in the prior decade: × mental health conditions	Not Assessed
**Canberra Bushfires (2003)**									
Parslow and Jorm [95] 2006 Tobacco Use After Experiencing a Major Natural Disaster: Analysis of a Longitudinal Study of 2063 Young Adults	Cohort 2,063 Representative sample of adults aged 20–24 on 1 January 1999, randomly sampled from electoral rolls for the cities of Canberra and Queanbeyan	Survey (format not specified) Entire study spanned 4 years Baseline: approximately 3 years pre-fire Follow-up: 3–18 months post-fire	PTSD, tobacco use	The number of fire-related experiences (i.e., evacuation from home/work, injury, destroyed home/possessions) were quantified	Trauma Screening Questionnaire (TSQ), tobacco use questions (whether participant had smoked tobacco in last 12 months; if so, how often they smoked and if smoking daily, the average number of cigarettes)	Stepwise logistic regression	Education, gender, time between fire and follow-up interview, and neuroticism	Overall study population: 5.0% PTSD at follow-up More fire-related experiences: + increased tobacco use from baseline to follow-up PTSD symptoms did not significantly contribute to explaining increased tobacco use.	Not Assessed
Parslow et al. [96] 2006 Associations of Pre-trauma Attributes and Trauma Exposure with Screening Positive for PTSD: Analysis of a Community-based Study of 2085 Young Adults	Cohort 2,085 Representative sample of adults aged 20–24 on 1 January 1999, randomly sampled from electoral rolls for the cities of the Australian Capital Territory and Queanbeyan	Survey (format not specified) Entire study spanned 4 years Baseline: approximately 3 years pre-fire Follow-up: 3–18 months post-fire	PTSD	The number of fire-related experiences (i.e., evacuation from home/work, injury, destroyed home/possessions) were quantified	TSQ	Negative binomial regression	Sex, education, pre-fire mental health status, neuroticism, social support and childhood adversity, and pre-fire experience of trauma	Overall study population: 5.0% PTSD Evacuated from home/work during fires, having friend/relative die or get injured from the fires: + PTSD	Not Assessed
McDermott et al. [97] 2005 Posttraumatic Stress Disorder and General Psychopathology in Children and Adolescents Following a Wildfire Disaster	Cross-sectional survey 222 All children in grades 4–12 attending a nondenominational private school in the Canberra wildfire disaster area	Practitioners and teachers supervised students’ completion of a school-based questionnaire screen for wildfire-related events. 6 months	General psychopathology, PTSD	The school of study was itself considered “wildfire affected.” Perception of the wildfire was measured by questions measuring the factors of physical proximity to flames, seeing smoke, seeing flames, thinking themselves and/or family members may die, being home alone during the fire, and home damage from the fire.	PTSD-RI, Strengths and Difficulties Questionnaire (SDQ)	Chi-square and t-tests	Not Assessed	9.0% severe or very severe PTSD Thought self might die, thought family member might die, close to flames, home alone, saw flames: + PTSD Saw smoke, thought self might die, thought family might die, home damage, lived elsewhere: + general psychopathology	Not Assessed
**Sutherland Bushfires (1994)**									
McDermott and Palmer [98] 2002 Postdisaster Emotional Distress, Depression and Event-related Variables: Findings Across Child and Adolescent Developmental Stages	Cross-sectional survey 2,379 Children in grades 4–12 attending state school in the disaster affected area	In-person survey 6 months	Anxiety, depression, distress	Exposure-related variables were the child’s location during the week and day of the bushfire, home damage, evacuation experience, residential disruption experience, and their perception of threat to themselves and/or their parents.	Birleson Depression Inventory, IES, Revised Manifest Anxiety Scale (RMAS)	Generalized linear models	Age, anxiety, gender, grade at school	Evacuation on the day of fires: + depression Evacuation during the week of the fires: - depression Evacuation, threat perception to self/parent: + distress	Not Assessed
**Painted Cave Fire (1990)**									
Jones et al. [99] 2002 Psychological Impact of Fire Disaster on Children and Their Parents	Case study 22 Families and individuals whose homes had sustained significant loss from the wildfire	In-person interview and psychiatric assessment 6 weeks	PTSD, psychosocial adjustment	Participants were classified by self-report as high loss or relatively low loss resulting from the fire. The Fire Questionnaire-Child Form (FQ-C) and the Fire Questionnaire-Adult Form (FQ-A), developed by the authors (unpublished manuscript, 1990), was administered, as was the Impact of Events Scale (IES) [250].	Diagnostic Interview for Children and Adolescents–Revised (DICA-R), Diagnostic Interview Schedule (DIS), State-Trait Anxiety Inventory for Children (STAI-C)	ANOVA and t-tests	Not Assessed	High loss group: + PTSD	Not Assessed
**Ash Wednesday Fires (1983)**									
McFarlane and van Hooff [100] 2009 Impact of Childhood Exposure to a Natural Disaster on Adult Mental Health: 20-year Longitudinal Follow-up Study	Cohort study 1,011 Primary school children in an area affected by the fires (bushfire-exposed cohort) and from a nearby unaffected area (controls)	Telephone interview Initial recruitment: 2-years Follow-up: 20 years	Alcohol use, anxiety, depression, distress	Students were considered exposed if they attended school in the bushfire-affected area.	AUDIT, Composite International Diagnostic Interview (CIDI), IES-R	Generalized estimating equations	Age, gender	Bushfire-exposed: 36.7% any DSM–IV psychiatric disorder during lifetime Controls: 31.7% any DSM–IV psychiatric disorder during lifetime Comparing bushfire-exposed to controls at follow-up: + anxiety - distress × alcohol use, depression, PTSD	Not Assessed
**Multiple/Unspecified**									
Mosca et al. [101] 2025 Eco-Anxiety and Mental Health: Correlates of Climate Change Distress	Cross-sectional 1,051 Italian adults	Online survey N/A	Eco-anxiety	Participants indicated their lifetime exposure to wildfires.	13-item Hogg Eco-Anxiety Scale (HEAS-13)	Independent samples t-tests	Not Assessed	Comparing participants who experienced wildfires to those who did not: + eco-anxiety	Not Assessed
Zhu et al. [102] 2024 Wildfires are Associated with Increased Emergency Department Visits for Anxiety Disorders in the Western United States	Case-crossover 1,897,865 emergency department visits for anxiety disorders Zip code-level emergency department visit data were obtained from Arizona (2010–2018), California (2007–2018), Nevada (2009–2016), Oregon (2014–2018), and Utah (2007–2016).	All secondary data sources N/A	Anxiety	Daily wildfire and non-wildfire PM_2.5_ concentrations were estimated based on satellite imagery and air quality monitoring data from 2007–2018.	ICD-9/10 codes for anxiety disorders, including anxiety, dissociative, stress-related, somatoform, and other nonpsychotic mental disorders	Conditional logistic regression	Federal holiday indicators; natural splines for the day of year, mean temperature, and relative humidity	A 10 µg/m^3^ increase in 48-hour exposure to wildfire PM_2.5_: + risk of anxiety-related emergency department visits in the general population	Age, sex
Giles et al. [103] 2024 Running Through the Haze: How Wildfire Smoke Affects Physical Activity and Mental Well-Being	Cross-sectional survey administered at two time points 348 provided necessary demographic data for the first survey with 162 completing both surveys Adults who responded to a snowball sampling campaign. The majority of participants (98.4%) were residents of British Columbia, Canada	Survey (format not specified) The first survey was completed during a wildfire smoke event with the second survey completed a month later not during a wildfire smoke event.	Anxiety, depression, stress	The first survey’s administration aligned with a period of wildfire smoke along the West Coast of Canada and the US, while the second survey’s did not. Where possible, the presence of wildfire smoke was confirmed with the mean daily PM_2.5_ from participant locations in British Columbia.	DASS-21	Poisson mixed model	Fixed effects for the presence/absence of wildfire smoke, random effects for the presence of a cardiorespiratory condition, and gender	During the period of wildfire smoke (first survey) compared to the period without wildfire smoke (second survey): + anxiety, depression, stress	Not Assessed
Tao et al. [104] 2024 Understanding Climate Change Anxiety and Anticipatory Climate Disaster Stress: A Survey of Residents in a High-risk California County During Wildfire Season	Cross-sectional 813 Residents of Lake County, California	Online survey N/A – distinct wildfire events were not under investigation, but the survey was administered at the start of the 2023 wildfire season	Climate change anxiety, anticipatory climate stress	Participants self-reported the number of times they had been in a disaster evacuation zone due to wildfires, landslides and/or floods; prior loss/injury due to wildfires (i.e., property loss, home destruction, personal injury, pet loss, knowledge of other people’s injury or death); and duration spent engaged with wildfire-related media content from different sources in the prior month.	Climate Anxiety Scale (functional and cognitive-emotional impairment); Participants self-reported the extent to which they anticipated different climate issues would be a significant source of stress over the next year, and a mean score across all issues was calculated.	Logistic and linear hierarchical regression	Age, duration of residence, gender, marital status, race/ethnicity	Evacuation zone experience: × climate change anxiety (functional and cognitive-emotional impairment) + anticipatory climate stress Wildfire-induced loss/injury: + climate change anxiety (functional and cognitive-emotional impairment), anticipatory climate stress Wildfire-related media exposure: + climate change anxiety (functional and cognitive-emotional impairment), anticipatory climate stress	Not Assessed
Wettstein and Vaidyanathan [105] 2024 Psychotropic Medication Prescriptions and Large California Wildfires	Cohort 7,115,690 Individuals residing in California metropolitan statistical areas (MSAs) with prescriptions of psychotropic medications recorded in the Merative MarketScan Research Database from September 2011 – November 2018	All secondary data sources N/A	Prescriptions of psychotropic medications (antidepressants, antipsychotics, anxiolytics, hypnotics, and mood-stabilizers)	Residential proximity to large wildfires that burned more than 25,000 acres in a California county within a MSA from September 2011 – November 2018	Prescription rates of psychotropic medications immediately following the start of the fire	Interrupted time-series analysis to compare psychotropic medication prescriptions in the 6 weeks before and after each of 25 wildfires	Co-occurrence of any extreme weather alerts and disaster declarations for other hazards during the fire or pre-fire period	In the fire period compared with the pre-fire baseline: + prescriptions of antidepressants, anxiolytics, and mood-stabilizing medications × antipsychotics, hypnotics, and statins (negative control outcome)	Age, sex
Molitor et al. [106] 2023 Air Pollution and Suicide in Rural and Urban America: Evidence from Wildfire Smoke	Quasi-experimental 484,848 county-year-month observations 3,108 US counties	All secondary data sources N/A	Suicide	Smoke days per month in each county, defined by medium or thicker smoke plumes by NOAA’s Hazard Mapping System	Monthly deaths by suicide at the county-level	Panel fixed effects regression to estimate the effect of smoke day exposure on suicide rates	Fixed effects for county-by-month of year, county-by-year, and year-month	Rural counties: + suicides with additional smoke days Urban counties: × suicides with additional smoke days	Age, educational attainment, race, rurality, sex
Jones et al. [107] 2003 Psychosocial Correlates of Wildfire Disaster: Post Disaster Adult Reactions	Cohort First time point: 46 adults Follow-up: 9 adults Residents of a Southern California city that experienced an unspecified wildfire	First time point: in-person interview with self-report instruments Follow-up: telephone interview First time point: 6 weeks Follow-up: 2 years	Anxiety, depression, PTSD	Wildfire victims were defined by having homes that sustained significant damage or had total destruction. Non-victims experienced the wildfire, but their homes were not damaged nor destroyed. The Fire Questionnaire-Adult Form was also used for additional fire-related events (e.g., events, feelings, losses)	PTSD module of the DIS, IES, BDI, STAI	ANOVA, MANOVA	Gender	Comparing victims and non-victims 6-weeks post-fire: + anxiety, depression, PTSD symptoms (total HIES scale score), PTSD intrusion symptoms (HIES), PTSD avoidance symptoms (HIES), number of PTSD symptoms (DIS), PTSD intrusion symptoms (DIS), PTSD arousal symptoms (DIS) × PTSD avoidance symptoms (DIS) Comparing victims at 6-weeks and 2-years post-fire: - PTSD symptoms (total HIES scale score), PTSD intrusion symptoms (HIES) × PTSD avoidance symptoms (HIES), PTSD intrusion symptoms (DIS), PTSD arousal symptoms (DIS), PTSD avoidance symptoms (DIS)	Not Assessed

^a^High = experienced major injury, deaths of one or more loved ones, felt their life was in danger, lost their home, or had remained displaced since the fire; medium = experienced evacuation, lost personal property (e.g., vehicles, shed), lost pets or farm animals, were forced to relocate, lost income, or if a loved one experienced a major injury; and low = in an area with high fire alert levels, lost one or more community buildings (e.g., child’s school, friend’s home), or were involved in fighting fires or providing a service in response to the fires

^b^Any exposure - yes/no if any direct exposures to fire were indicated; scaled - three levels of exposure - none (none or indirect), mild (being in an area with fire nearby, evacuation due to bushfire, area of significance lost other than home, family member was affected, home was affected while away), and severe (loss of or damage to property or direct contact with fire e.g., firefighter or protecting property); and cumulative - the number of ways in which the participant had previously been exposed to fire were added

^c^Resided = whether respondents resided in Fort McMurray during the wildfire

^d^Property loss = whether respondents lost property or business from the wildfire

^e^Witnessed burning = whether respondents witnessed the burning of any homes or structures by the wildfire

^f^Fearful = whether respondents were fearful for their life or the lives of their friends or family on the day of evacuation

^g^Watch frequency = the frequency in which respondents watched TV news about the fire devastation

^h^Read frequency = the frequency in which respondents read newspaper and internet articles related to the fire devastation

^i^Volume of damaged properties = the area of respondents' residence relative to the volume of fire-damaged properties

^j^Living situation = whether respondents live in the same residence they lived in before the evacuation order

^k^Home completely destroyed = home was completely destroyed by the wildfire

^l^Home substantially destroyed = home was substantially destroyed by the wildfire

^m^Business substantially destroyed = business/place of employment was substantially destroyed by the wildfire

^n^IOF = a custom questionnaire to assess the impact of the 2016 wildfire, with exposure-related questions of whether the participant was present in Fort McMurray during the fire, whether they were evacuated, whether they personally saw the fire, and whether their home was destroyed

^o^Smoke damage = home suffered substantial smoke damage from the wildfire

^p^No loss = respondent suffered no loss of property or business from the wildfire

^q^Post-fire residence = where the respondent lived after the fire relative to where they lived before the fire

^r^Events at the time of the fire = respondents' presence in Fort McMurray, whether evacuated, direction of evacuation, and sleeping arrangements during the first couple of days after the fire

^s^Events since the fire = damage to respondents' own neighborhood, financial loss, resumption of paid employment, and residence in Fort McMurray at follow-up

**Table 2:** Included studies with exposed-only cohorts

Author Year Title	Study type Size Population	Data collection methods Time of collection post-fire	Mental health outcomes	Exposure measure	Outcome measure	Statistical methods	Confounding adjustment	Prevalence	Risk factors
**Canadian Wildfires (2023)**									
Barrera et al. [108] 2025 Perceptions of and Responses to Wildfire Smoke Among New York State Residents: A Cross-Sectional Study	Cross-sectional 609 New York state residents in the summer of 2023	Online survey 4–5 months after the peak smoke wave (June 2023) from the Canadian wildfires	Anxiety	Responding “yes” to the question “During the summer of 2023, did you experience one or more days that were smokey, or days where you felt the air quality was poor due to wildfire smoke?”	Indicating “anxiety” to the question “Did you have any of the following symptoms during or a few days after one of the smoke events in Summer 2023?”	Descriptive statistics	Not Assessed	19.5% anxiety (not compared across exposure groups)	Not Assessed
Obuobi-Donkor et al. [109] 2024 Evaluating the 3-month Post-intervention Impact of a Supportive Text Message Program on Mental Health Outcomes During the 2023 Wildfires in Alberta and Nova Scotia, Canada	Cohort 150 Residents of Alberta and Nova Scotia during the wildfires who had subscribed to Text4Hope mental health support services	Online survey During the wildfires; baseline data were collected 2–3 months after their ignition, with follow-up data after receiving the Text4Hope mental health support services collected 5–9 months after their ignition.	GAD, MDD, resilience, PTSD, and mental well-being	All participants were considered exposed given their residence in Alberta or Nova Scotia during the wildfires.	GAD-7, PHQ-9, BRS, PCL-C, World Health Organization-5 Well-being Index (WHO-5)	Chi-square and paired sample t-tests	Not Assessed	Baseline: 42.1% likely GAD 55.7% likely MDD 42.0% likely PTSD 55.1% low resilience 71.6% poor mental well-being Follow-up: 33.3% likely GAD 47.8% likely MDD 38.4% likely PTSD 53.4% low resilience 48.3% poor mental well-being	Not Assessed
**Black Summer Bushfires (2019–2020)**									
Halcomb et al. [110] 2023 Impacts of the 2019/20 Bushfires and COVID-19 Pandemic on the Physical and Mental Health of Older Australians: A Cross-sectional Survey	Cross-sectional survey 155 Community dwelling older people (aged 65 years and over) living in the South-eastern New South Wales, Australia during the disaster period	Online survey questionnaire (6 community groups received hard copies) 19–21 months	Self-report of mental health impacts	Median scores of bushfire impact were calculated based on responses to survey questions that assessed experiences with home evacuations, evacuation orders, home/property damage, personal safety threatened, and disruptions in everyday activities caused by the fire. However, in the assessment of mental health impact, participants were not aggregated by bushfire impact score, so all were considered exposed.	EuroQol-Visual Analogue Scale (EQ-VAS) for self-rated health, Connor-Davidson Resilience Scale (CD-RISC-2)	Mann–Whitney U test, Kruskal–Wallis H test, Wilcoxon signed-rank test and Spearman’s rank-order correlation coefficient	Not Assessed	86.2% anxious/worried Those who felt more impacted by the bushfires had lower resilience.Bushfires and bushfire smoke negatively impacted mental health more than the COVID-19 pandemic.	Not Assessed
Usher et al. [111] 2022 Coping Styles and Mental Health Outcomes of Community Members Affected by Black Summer 2019-20 Bushfires in Australia	Cross-sectional descriptive correlational design 405 Community members self-identified as being affected by the 2019–2020 Australian bushfires	Online survey questionnaire 0–12 months	Anxiety, coping styles, depression, stress	All participants were defined as exposed to the bushfires.	Coping Orientations to Problems Experienced (COPE) Inventory, DASS-21, IES-R	Bivariate correlations and independent samples t-tests	Not Assessed	Severe anxiety Moderate depression Moderate stress Approach and avoidance coping strategies Posttraumatic stress symptoms (i.e., intrusive thoughts and symptoms of avoidance and hyperarousal)	Not Assessed
**Goseong Fire (2019)**									
Hong et al. [112] 2022 Mental Health Effects of the Gangwon Wildfires	Panel study with 1, 3 and 6 month follow-ups 206 Adult fire survivors who completed an initial psychological support services assessment and agreed to be contacted for follow-up counseling	The outreach team conducted initial in-person baseline interviews and subsequent interviews were over the phone. 0–1 months (initial assessment)	Anxiety, depression, grief, insomnia	All survivors who received psychological support services from the “Integrated Mental Health Service Team for Wildfires” were considered exposed.	Post-disaster Psychological Responses-Checklist	Linear mixed models with repeated measures	Not Assessed	Baseline assessment: 50.0% anxiety 32.5% depression 33.0% grief 59.2% insomnia Mental health improved over time.	Experience of flashbacks
**Fort McMurray Wildfire (2016)**									
Belleville et al. [113] 2023 Efficacy of a Therapist-Assisted Self-Help Internet-Based Intervention Targeting PTSD, Depression, and Insomnia Symptoms After a Disaster: A Randomized Controlled Trial	Randomized Control Trial 136 Residents of Fort McMurray who reported either (a) moderate or mild symptoms of PTSD or (b) mild symptoms of PTSD with moderate symptoms of depression or subthreshold insomnia	Participants used a therapist-guided, online self-help treatment. Data on the outcomes of interest were collected via phone interview. 24–32 months	Anxiety, depression, disability, insomnia, PTSD	All participants were considered exposed given their Fort McMurray residence. Level of exposure to the fire was assessed by a previously developed survey [59].	GAD-7, ISI, PHQ-9, PCL-5, World Health Organization Disability Assessment Schedule 2.0 (WHODAS 2.0)	Intent-to-treat mixed models ANOVAs	Not Assessed	Pretreatment Mean Scores Across All Treatment Groups: PCL-5: 26.2 PHQ-9: 9.7 ISI: 16.1 GAD-7: 8.2 WHODAS: 68.2	Not Assessed
Binet et al. [114] 2021 A Portrait of Mental Health Services Utilization and Perceived Barriers to Care in Men and Women Evacuated During the 2016 Fort McMurray Wildfires	Cross-sectional survey 1,510 Evacuees from the Fort McMurray wildfire who were at least 18 years old and spoke sufficient English	Telephone survey 12–14 months	Depression, insomnia, PTSD	All participants were considered exposed; defined by having to evacuate from the fire. Participants were asked exposure-related questions, but these data were not used in the analyses to predict the mental health outcomes.	ISI, PCL-5, PHQ-9	Chi-square tests and multiple logistic regression	Not Assessed	PTSD 17.7% probable diagnosis 21.5% subclinical symptoms Depression 7.8% probable diagnosis 5.2% subclinical symptoms Insomnia 13.5% probable diagnosis 23.3% subclinical symptoms	Sex
Brown et al. [115] 2021 Mental Health Symptoms Unexpectedly Increased in Students Aged 11–19 Years During the 3.5 Years After the 2016 Fort McMurray Wildfire: Findings from 9,376 Survey Responses.	Panel study 9,376 (sample size, not unique number of participants) Grade 7–12 students enrolled in either junior or senior high schools in both Public and Catholic School Districts in Fort McMurray in 2017	An initial intake survey, with mental health surveys were administered in-person, during class time for 3 consecutive years. 18, 30, and 42 months	Anxiety, depression, PTSD, quality of life, resilience, self-esteem, substance use, suicidal thinking	The Impact of Fire Questionnaire (IOF)^a^ was administered. However, students were not classified by their level of impact; thus, all participants were considered exposed.	CYRM-12, CRAFFT Questionnaire, CPSS, HADS, Kidscreen-10, PHQ-A, Rosenberg Self-Esteem Scale	Permutation testing on the slope parameter from a fitted linear model	Not Assessed	2019 prevalence (direction of change relative to 2017 prevalence): 41.0% probable PTSD (*↑*) 35.0% probable depression (*↑*) 20.0% probable moderately severe depression (*↑*) 18.0% suicidal thinking (*↑*) 31.0% probable anxiety (*↑*) 16.0% probable alcohol/ substance use disorder (*↑*) 12.0% tobacco use (*↓*) Self-esteem and quality of life scores decreased. Resilience scores did not change significantly.	Age and gender identity
Belleville et al. [116] 2019 Post-Traumatic Stress Among Evacuees from the 2016 Fort McMurray Wildfires: Exploration of Psychological and Sleep Symptoms Three Months After the Evacuation	Cross-sectional survey 379 (of which 55 participated in a clinical interview) Evacuees from the Fort McMurray wildfire who were at least 18 years old and spoke sufficient English	Online questionnaire survey and optional follow-up clinical interview 2–4 months	Depression, insomnia, PTSD	Exposure was not explicitly measured - participants were evacuees from the wildfire.	Online Survey: ISI, PCL-5, PHQ, PHQ-9, Pittsburgh Sleep Quality Index and its Addendum for PTSD (PSQI; PSQI-A), the Post-Traumatic Cognitions Inventory (PTCI) Clinical Interview: Clinician- Administered PTSD Scale (CAPS), Mini International Neuropsychiatric Interview (MINI)	Pearson’s correlations, hierarchical multiple regression	Not Assessed	Prevalence from questionnaire: 62.5% PTSD Confirmed by interview: 29.1% PTSD, 25.5% depression, and 43.6% insomnia	Age
**Bastrop County Complex Fire (2011)**									
Kirsch et al. [117] 2016 Longitudinal Community Assessment for Public Health Emergency Response to Wildfire, Bastrop County, Texas	Cross-sectional household-level survey repeated at two time points First survey: 135 households Follow-up survey: 185 households 35 randomly selected census blocks within, overlapping, or touching the Bastrop County Complex Wildfire perimeter. Seven households within each census block were intended to be interviewed, for a total of 245 target surveys.	In-person survey at participants’ homes First survey: 20–21 days post-ignition Follow-up: 3.5 years	Depressed mood/hopelessness, sleeping problems, stress	Households were considered exposed if their residence fell within, overlapped, or touched the fire perimeter.	Mental health questions were derived from the Center for Disease Control and Prevention’s (CDC’s) Behavioral Risk Factor Surveillance System (BRFSS) (e.g., “Over the past 2 weeks, how often have you felt down, depressed, or hopeless?”).	Descriptive statistics, risk differences	Not Assessed	First survey: 54.8% of adults experienced a depressed mood/hopelessness in the previous 2 weeks 54.8% of adults experienced sleeping problems in the previous 2 weeks 30.8% of children experienced sleeping disturbances in the previous 2 weeks Follow up (2015): Comparing those that sustained damage to their homes to those who did not, 11.5% of households reported sleep disturbances as a long-term mental health impact of the wildfire on their children. Respondents exposed to the 2011 wildfire were at greater risk of depressive symptoms (risk difference = 5.6%). Those exposed to the 2011 wildfire reported significantly higher stress in 2015 than in 2011, compared to those unexposed to the wildfire (27.3% versus 8.1%).	Residing in homes that sustained wildfire damage
**Mount Carmel Forest Fire (2010)**									
Hashoul-Andary et al. [118] 2016 A Longitudinal Study of Emotional Distress Intolerance and Psychopathology Following Exposure to a Potentially Traumatic Event in a Community Sample	Cohort 151 Adults who had to evacuate their homes due to the fire and who met the DSM-IV criterion A of PTSD (i.e., felt intense fear, horror or helplessness in response to the fire)	Online or hard copy survey Time point 1: within 30 days Time point 2: 3 months Time point 3: 6 months	Anxiety, anxiety sensitivity, depression, distress, post-traumatic stress, suicidality	All participants were considered exposed given they evacuated from the fire. The Carmel Trauma Questionnaire was developed to measure participants’ proximity to the fire, injury to the participant or their relatives, and property damage.	Anxiety Sensitivity Index-3 (AS-I-3), Distress Tolerance Scale (DTS), Inventory of Depression and Anxiety Symptoms (IDAS), Posttraumatic Diagnostic Scale (PDS)	Structural equation modeling	Not Assessed	Mean scores on scales from time point 1 to time point 3: AS-I-3 decreased DTS decreased PDS decreased IDAS Depression decreased (but increased from time point 2 to time point 3) IDAS Suicidality increased	Not Assessed
Zeller et al. [119] 2015 Self-Compassion in Recovery Following Potentially Traumatic Stress: Longitudinal Study of At-Risk Youth	Cohort 64 High school students living in an educational residential youth village in northern Israel and who had proximal exposure to the fire in the 30 days prior to initial survey administration	Online survey Time point 1: within 4 weeks Time point 2: 3 months Time point 3: 6 months	Depression, panic, post-traumatic stress, suicidality, well-being	All participants were considered exposed given they evacuated from the fire. The Carmel Trauma Questionnaire was developed to measure participants’ proximity to the fire, injury to the participant or their relatives, and property damage. As the hypothesized mediator, self-compassion was measured with the Self Compassion Scale.	IDAS	Multilevel modelling of mediation	Not Assessed	Comparing symptoms at time point 1 to time point 3: Panic increased Post-traumatic stress increased Depression did not change Suicidality did not change Well-being did not change Self-compassion mediated symptoms of posttraumatic stress, panic, depression, and suicidality.	Not Assessed
**Black Saturday Bushfires (2009)**									
Wasiak et al. [120] 2013 12-month Generic Health Status and Psychological Distress Outcomes Following an Australian Natural Disaster Experience: 2009 Black Saturday Wildfires	Case study 15 Patients admitted to Victorian Adult Burns Unit with burn injuries as a result of the Black Saturday Wildfires	Baseline in-clinic interview within 21 days of admission with follow-up phone interviews at 3, 6, and 12 months	Alcohol use, psychological distress	All patients were considered exposed.	AUDIT, K-10, 36-item Short Form Health Survey (SF-36)	Generalized linear models	Not Assessed	At 3 and 6 months: 33.0% high to very high psychological distress At 12 months: 27.0% high psychological distress Across study period: No changes in AUDIT scores	Not Assessed
**Greek Forest Fires (2007)**									
Psarros et al. [121] 2017 Insomnia and PTSD One Month After Wildfires: Evidence for an Independent Role of the “Fear of Imminent Death”	Cross-sectional survey 92 Randomly selected victims of wildfires who were permanent residents in the Greek province of Ilia in 2007	In-person interviews and questionnaires 1 month	Insomnia, PTSD	All victims were considered exposed.	Athens Insomnia Scale (AIS), ICD-10 diagnostic criteria of PTSD	Univariate and multiple logistic regression	Not Assessed	63.0% insomnia 46.7% PTSD	Age, sex, fear of death, PTSD
**Canberra Bushfires (2003)**									
Camilleri et al. [122] 2010 Recovery from Bushfires: The Experience of the 2003 Canberra Bushfires Three Years After	Cross-sectional 500 Canberra households that had been registered with the Bushfire Recovery Centre	Paper survey 3.25 years	Psychological distress	Participants reported their experiences of threats and losses as a result of the bushfire, but these were not analyzed in association with psychological distress.	K-10	Descriptive statistics	Not Assessed	19.5% high to very high distress over the past four weeks 11.5% high levels of psychological distress 8.0% very high levels of psychological distress	Not Assessed
**Multiple/Unspecified**									
Isaac et al. [123] 2025 Digital Cognitive Behavioral Therapy–Based Treatment for Insomnia, Nightmares, and Posttraumatic Stress Disorder Symptoms in Survivors of Wildfires: Pilot Randomized Feasibility Trial	Randomized control trial 30 Wildfire survivors from Australia, Canada, and the US recruited from May - December 2023 who had met at least one of the criteria: a score of ≥ 8 on the ISI, a score of ≥ 3 on the Nightmare Disorder Index, or a score of ≥ 31 on the PCL-5	Online surveys and treatment modules Variable; mental health assessments performed at baseline, posttreatment, and at a 3 month follow-up	Anxiety, depression, insomnia, PTSD	All participants were considered exposed given their indication of being wildfire survivors.	GAD-7, ISI, PHQ-9, PCL-5	Intent-to-treat analysis (primary) and per-protocol analysis (secondary) with mixed-effects models	Not Assessed	Intent-to-Treat Pretreatment Mean Scores Across All Treatment Groups: GAD-7: 1.9 ISI: 3.4 PCL-5: 48.4 PHQ-9: 12.5	Not Assessed
Isaac et al. [124] 2023 Differences in Anxiety, Insomnia, and Trauma Symptoms in Wildfire Survivors from Australia, Canada, and the United States of America	Cross-sectional 126 Wildfire survivors from Australia, Canada, and the US	Online survey Unspecified	Anxiety, depression, insomnia, and PTSD symptoms	Participants indicated if they had experienced the wildfire event more or less than 12 months from when they completed the survey.	GAD-7, ISI, PHQ-9, PCL-5	ANCOVA	Not Assessed	**Australia**: 35.1% moderate to severe anxiety 44.2% moderate to severe depression 34.1% moderate to severe insomnia 48.6% above clinical PTSD threshold **Canada**: 37.0% moderate to severe anxiety 55.6% moderate to severe depression 59.2% moderate to severe insomnia 75% above clinical PTSD threshold **US**: 63.0% moderate to severe anxiety 57.4% moderate to severe depression 56.4% moderate to severe insomnia 88.9% above clinical PTSD threshold	Gender, education level, employment, income, and recency of fires
Hooper et al. [125] 2018 Life After Bushfire: Post-traumatic Stress, Coping and Post-traumatic Growth	Cross-sectional 65 Adults aged 19–65 who indicated they had experienced one or more Australian bushfire events	Online survey Variable – exact bushfire event not specified	Post-traumatic stress, post-traumatic growth	Survey questions included the number of bushfire events experienced, time since most recent bushfire experience, having lived in a house or property directly impacted by bushfire, and bushfire evacuation of self, family, or friends.	IES-R, Post-Traumatic Growth Inventory (PTGI)	Generalized linear regression	Age, time since fire	Prevalence not assessed. Longer time since the most recent bushfire experience was associated with decreased post-traumatic stress and growth.	Not Assessed

^a^IOF = a custom questionnaire to assess the impact of the 2016 wildfire, with exposure-related questions of whether the participant was present in Fort McMurray during the fire, whether they were evacuated, whether they personally saw the fire, and whether their home was destroyed

## Results

### Study Selection

**Fig. 1:**
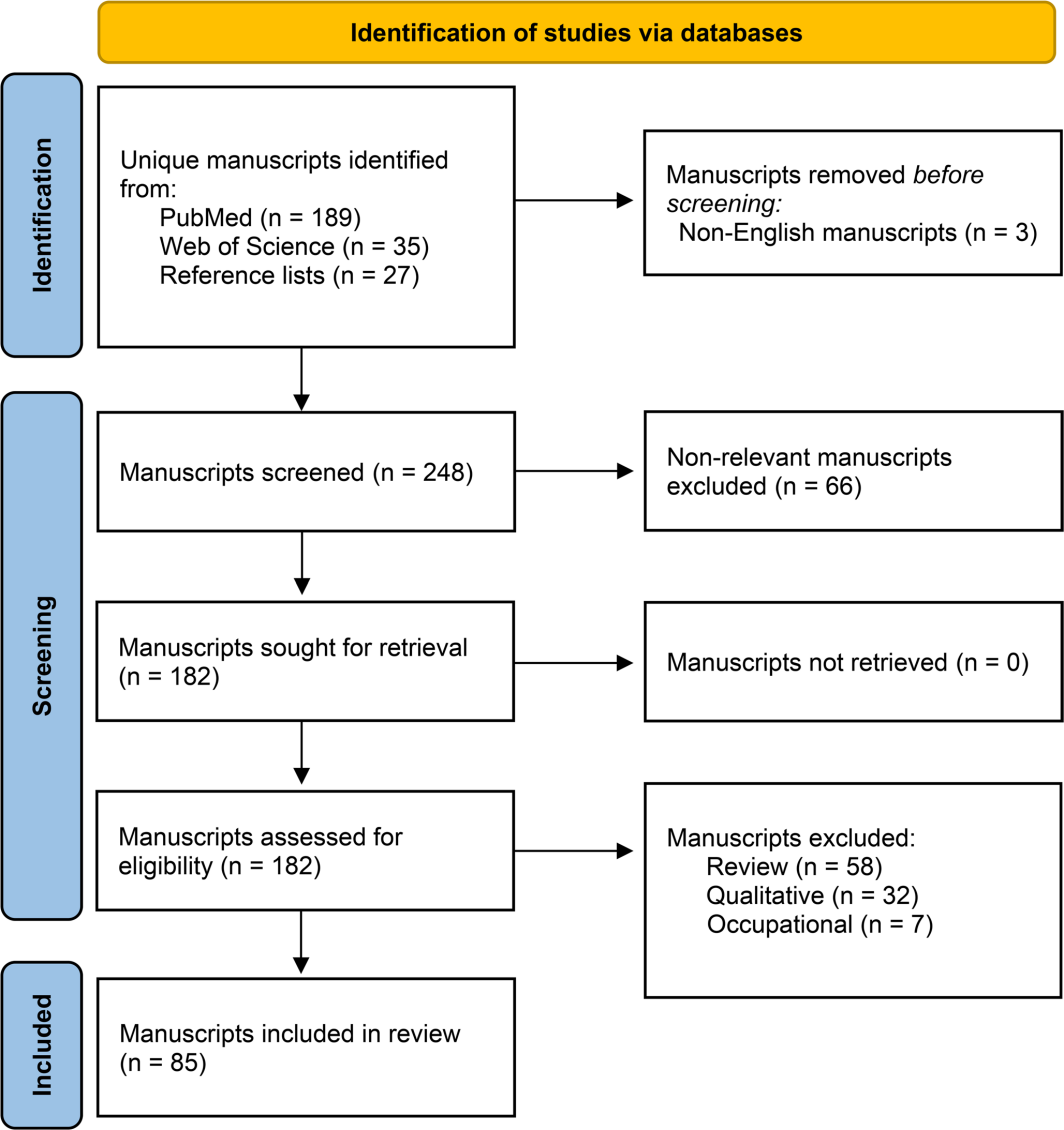
Flow chart of literature review

Our search criteria yielded 251 initial papers, with 85 included in the final literature sample that met our inclusion criteria (Fig. 1). We excluded the following manuscripts: those not relevant to our investigation topic (*n* = 66), reviews (*n* = 58), qualitative studies (*n* = 32), and non-English studies (*n* = 3). Given that the focus of this paper is on community exposure, we also excluded studies that assessed occupational exposure among firefighters (*n* = 7). 

### Study Distribution

**Fig. 2:**
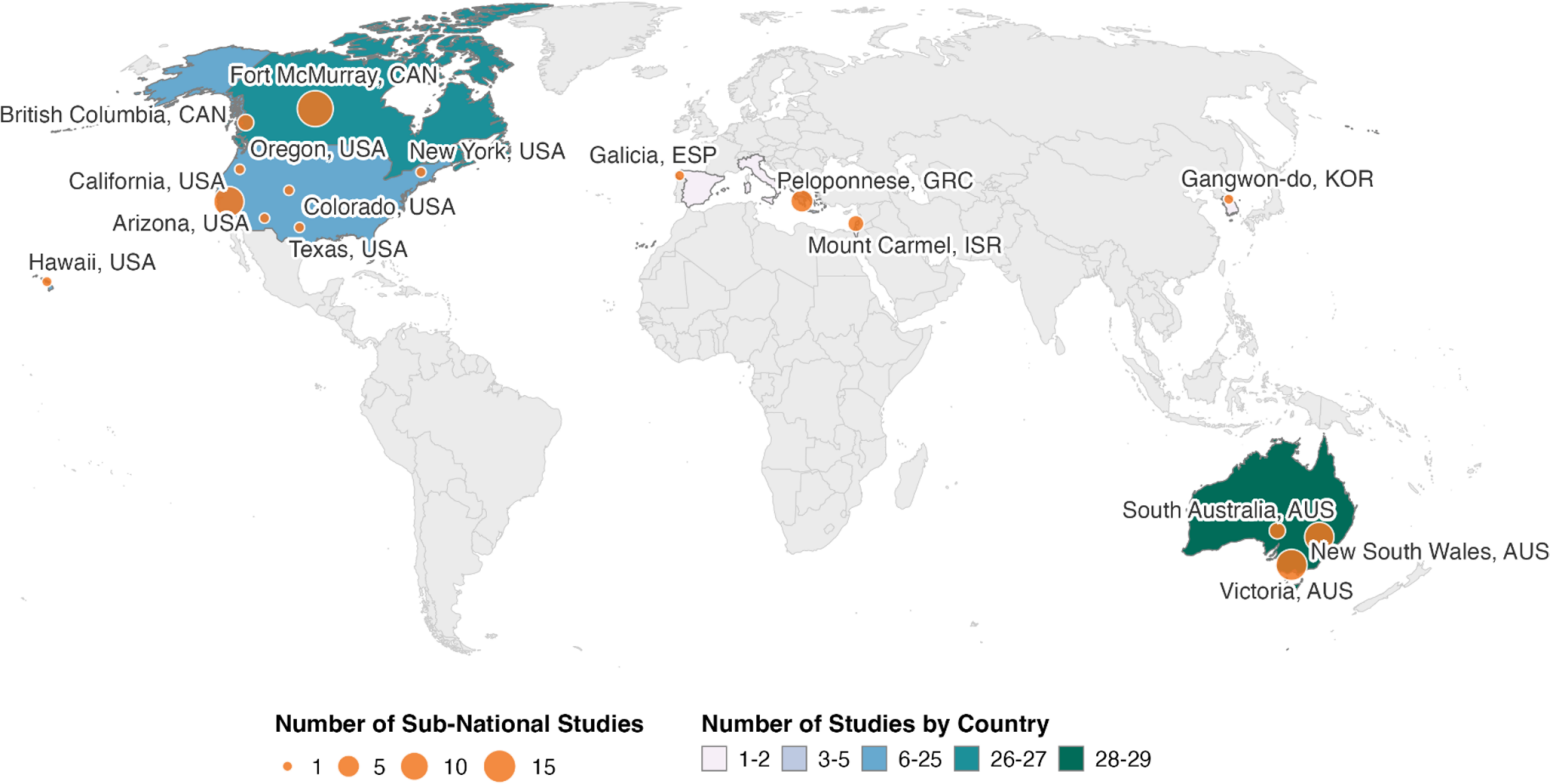
Geographic and proportional distribution of included studies. Circle size is proportional to the number of identified studies conducted at sub-national scales (i.e., city, province, region) that evaluated mental health outcomes. Categories of the number of studies by country were defined using Jenks natural breaks classification

There was a wide geographic scope in the identified articles (Fig. 2). A majority of studies (*n* = 19) assessed mental health outcomes in Alberta, Canada (Fort McMurray wildfire) [52–66, 113–116]. Other studies took place in California, US (*n* = 14) [37, 38, 43, 45, 51, 81, 82, 85, 92, 93, 99, 104, 105, 107], Victoria, Australia (*n* = 13) [70–80, 100, 120], New South Wales, Australia (*n* = 11) [46–49, 95–98, 110, 111, 122], Greece (*n* = 5) [87–90, 121], the US (regional/whole country) (*n* = 5) [102, 103, 106, 123, 124], Alberta and Nova Scotia, Canada (*n* = 4) [39–41, 109], Australia (whole country) (*n* = 3) [123–125], British Columbia, Canada (*n* = 2) [94, 103], Mount Carmel, Israel (*n* = 2) [118, 119], Canada (whole country) (*n* = 2) [123, 124], South Australia (*n* = 2) [91, 100], Arizona, US (*n* = 1) [68], Colorado, US (*n* = 1) [67], Hawaii, US (*n* = 1) [42], Italy (*n* = 1) [101], New York state, US (*n* = 1) [108], Oregon, US (*n* = 1) [50], South Korea (*n* = 1) [112], Spain (*n* = 1) [86], and Texas (*n* = 1) [117]. 

### Wildfire Events

Among articles yielded in our search, there were 25 wildfires or combined wildfire events investigated, spanning 30 years from 1983 to 2025 (Table 3). The majority of fires resulted in multiple investigations: the Fort McMurray wildfire in 2016 (*n* = 19 papers) [52–66, 113–116], the Black Summer bushfires in 2020 (*n* = 6) [46–49, 110, 111], the Black Saturday bushfires in 2009 (*n* = 12) [70–80, 120], Greek forest fires in 2007 (*n* = 5) [87–90, 121], the Canadian wildfires of 2023 (*n* = 5) [39–41, 108, 109], the Canberra bushfires of 2003 (*n* = 4) [95–97, 122], the 2025 Los Angeles wildfires (*n* = 2) [37, 38], the California wildfires of 2020 (*n* = 2) [43, 45], the Southern California wildfires of 2003 (*n* = 2) [92, 93], the Mount Carmel forest fire in 2010 (*n* = 2) [118, 119], and the Santa Barbara, California Gap, Tea, and Jesusita fires in 2008–2009 (*n* = 2) [81, 82]. A number of papers either did not investigate unique wildfire events (e.g., investigating days of wildfire smoke exposure over extended periods) or did not specify the wildfire event under study (*n* = 8) [101–107].

**Table 3:** Specific wildfire events studied in the identified literature

Date	Wildfire name	Location	Acres burned	Structures destroyed	Direct fatalities	Cause	Number of studies	Studied in
January 2025	Los Angeles Wildfires	Los Angeles, CA, USA [126]	57,529 [127]	18,000+	32–440	Various, fueled by drought and Santa Ana winds	2	[37, 38]
August 2023	Maui Wildfire	Maui, HI, USA [128]	6,693	2,200+ [129]	102	Downed power lines, fueled by hurricane winds	1	[42]
March – October 2023	Canadian Wildfires	All Canadian Provinces [130]	37,065,800+	200+ [131]	8 [132]	Extreme weather	5	[39–41, 108, 109]
July – October 2020	California Wildfires	California, USA [133]	4,304,379	11,116	33	Various, fueled by extreme weather	2	[43, 45]
June 2019 – March 2020	Black Summer Bushfires	Multiple locations, AUS [134]	46,000,000+ [48]	6,000+	33	Various, fueled by extreme drought	6	[46–49, 110, 111]
April 2019	Goseong Fire	Gangwon Province, KOR [135]	1,300+ [136]	2,000+	2	Transformer spark	1	[112]
January – December 2018	Oregon Wildfires	Oregon, USA [50]	900,000+	0	1 [137]	Various	1	[50]
November 2016	Camp Fire	Paradise, CA, USA [138]	150,000+	18,000+	85	Downed power lines	1	[51]
May 2016	Fort McMurray Wildfire	Fort McMurray, CAN [139]	1,432,600	2,400+	2	Suspected human causes and extreme weather	19	[53, 55–66, 113–116, 140, 141]
June 2013	Royal Gorge and Black Forest Wildfires	Colorado, USA [142, 143]	17,480	557	2	Human causes	1	[67]
September – October 2011	Bastrop County Complex Fire	Bastrop County, TX, USA [144]	32,000+	1,600+	2	Downed power lines, fueled by tropical storm winds [145]	1	[117]
May – June 2011	Wallow Fire	Alpine, AZ, USA [146]	538,000+	62	0 [147]	Campfire	1	[68]
December 2010	Mount Carmel Forest Fire	Mount Carmel, ISR [148]	8,000+	250 [149]	44	Human causes	2	[118, 119]
February 2009	Black Saturday Bushfires	Victoria, AUS [150]	1,100,000+	3,500+ [151]	173	Extreme weather and arson	12	[70–80, 120]
June 2008 – May 2009	Gap, Tea, and Jesusita Fires	Santa Barbara, CA, USA [152–154]	20,000+	200+	0	Human causes	2	[81, 82]
October 2007	Witch Creek and Guejito Fires	San Diego, CA, USA [155]	500,000+ [156]	1,738	10	Downed power lines	1	[85]
August 2007	Greek Forest Fires	Peloponnese, GRC [157]	1,655,000+	2,100 [158]	85 [159]	Extreme weather	5	[87–90, 121]
August 2006	Spain Forest Fires	Galicia, ESP [160]	200,000+	0	4 [161]	Drought and arson	1	[86]
January 2005	Eyre Peninsula Bushfire	Lower Eyre Peninsula, South Australia, AUS [162]	202,600+	400+	9	Extreme weather	1	[91]
October – November 2003	Southern California Wildfires	Southern California, CA, USA [163]	740,000+	3,600+	22	Human causes and extreme weather	2	[92, 93]
August – September 2003	British Columbia Forest Fires	British Columbia, CAN [164]	107,600+	343	0	Various, fueled by extreme weather [165]	1	[94]
January 2003	Canberra Bushfires	Canberra, AUS [166]	395,000+	510	4	Extreme weather	4	[95–97, 122]
January 1994	Sutherland Bushfires	Sutherland Shire, New South Wales, AUS [167]	1,482,630+	200	4	Extreme weather	1	[98]
June 1990	Painted Cave Fire	Santa Barbara, CA, USA [168]	5,000+ [169]	500	1	Arson	1	[99]
February 1983	Ash Wednesday Fires	Victoria and South Australia, AUS [170]	439,000+	2,080	75	Extreme weather	1	[100]

### Timing of Data Collection

For the majority of studies (*n* = 49), initial exposure and outcome data were collected within two years of the wildfire event. For the studies that did not collect data within two years (*n* = 24), data collection ranged from 24 months to 20 years after the wildfire event. Studies that used secondary data sources (i.e., medical or pharmaceutical records) did not specify the timing of data collection (*n* = 8) [38, 43, 46, 67, 94, 102, 105, 106]. Studies that evaluated the mental health impacts of multiple wildfire events also did not specify the timing of data collection relative to the wildfires (*n* = 4) [101, 123–125]. In an investigation of the maternal mental health impacts of the Fort McMurray wildfire, the timing of data collection was not specified, although the recruitment for the study ran from 6 to 18 months after the wildfire [57]. Another study assessed aggregate, past wildfire exposures in a highly-exposed Northern California county but specified that their survey was administered at the start of the 2023 wildfire season [104]. Three studies were unique in that baseline data were available pre-wildfire event [64, 95, 96]. In one, students in Red Deer, Canada received a battery of mental health questionnaires in 2014, which investigators used to compare with the mental health assessments of Fort McMurray students that were administered 18 months after the wildfire [64]. The other two studies compared survey data collected 3 years prior to the Canberra Bushfires with data collected 3–18 months after the bushfires among the same sample [95, 96].

### Study Design

**Fig. 3:**
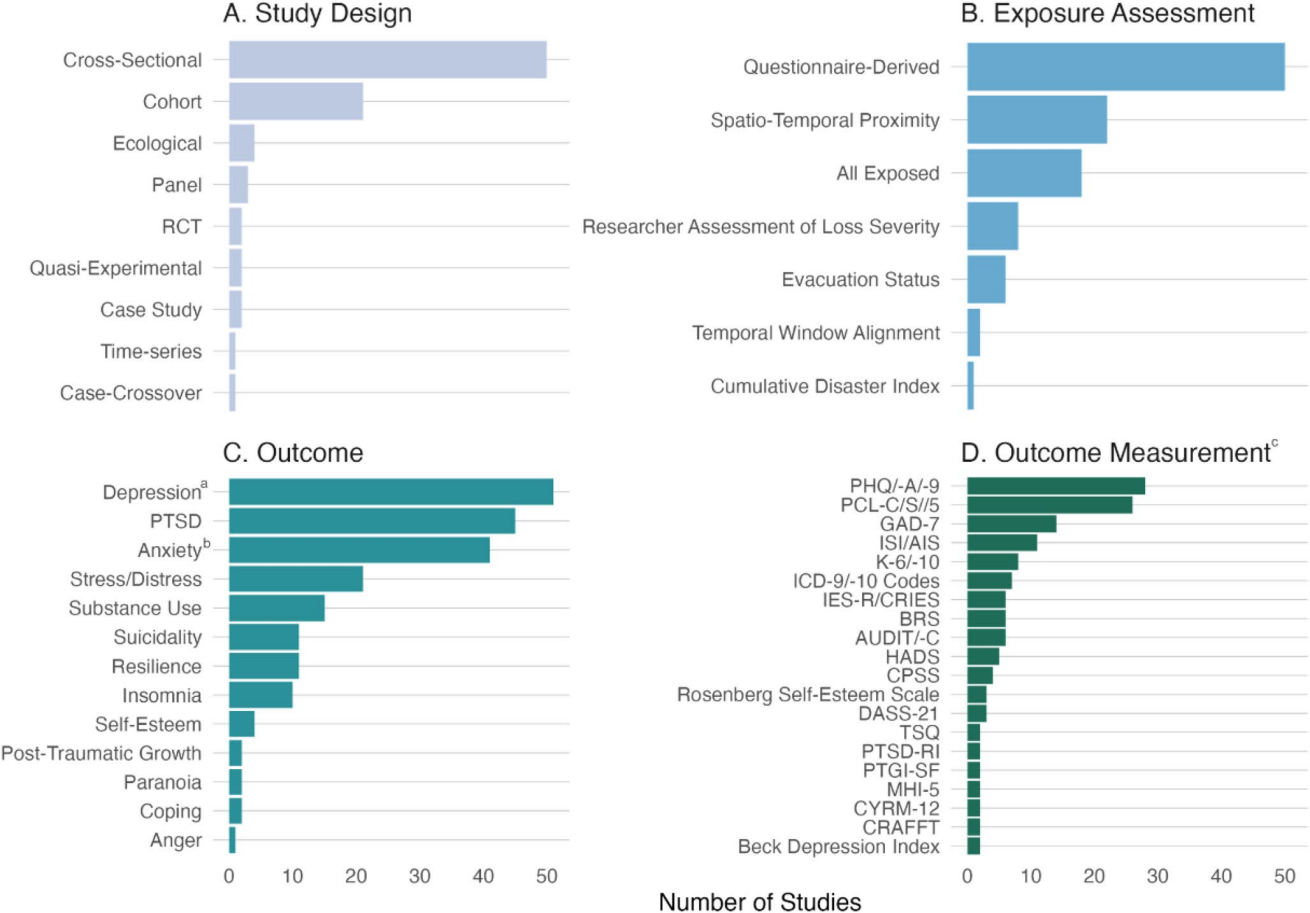
Characteristics of included studies by (**A**) study design; (**B**) exposure assessment; (**C**) outcome; and (**D**) outcome measurement. ^a^Includes Major Depressive Disorder and self-reported/not clinically diagnosed depression. ^b^Includes Generalized Anxiety Disorder and self-reported/not clinically diagnosed anxiety. ^c^Many studies used multiple outcome measurement tools, including those used to assess effect modifiers. Only outcome measurements that assessed the primary outcomes of interests and were used across multiple studies were included in this figure

The majority of studies in this review were cross-sectional and employed survey-based data collection methods (*n* = 50) [37, 39–41, 47–57, 59–65, 68, 71, 73, 80–82, 85, 87–91, 97, 98, 101, 103, 104, 108, 110, 111, 114, 116, 117, 121, 122, 124, 125] (Fig. 3a). The other studies were either cohort studies (*n* = 21) [38, 42, 66, 70, 72, 74–79, 92, 93, 95, 96, 100, 105, 107, 109, 118, 119], ecological studies (*n* = 4) [46, 67, 86, 94], panels (*n* = 3) [58, 112, 115], case studies (*n* = 2) [99, 120], quasi-experimental studies (*n* = 2) [45, 106], randomized control trials (RCT) (*n* = 2) [113, 123], a case-crossover study (*n* = 1) [102], or a time-series study (*n* = 1) [43]. The RCTs both tested cognitive behavioral therapy interventions among wildfire survivors. We do not leverage the RCT design and only include the pre-treatment scores on the measures of mental health, as they best approximate baseline mental health status following the wildfire exposures.

### Exposure Assessment

Assessment of the length or severity of exposure to the wildfire events varied widely and was largely non-systematic, with numerous studies using multiple methods (Fig. 3b). The most prominent method of exposure assessment was questionnaire-derived, in which participants self-reported their perceived level of exposure (e.g., “Have you been directly affected by any of the bushfires over the past year?” [48]) (*n* = 50) [37, 39–41, 43, 47–57, 59–65, 68, 71, 73, 80–82, 85, 87–91, 97, 98, 101, 103, 104, 108, 110, 111, 114, 116, 117, 121, 122, 124, 125]. The next most common method of exposure assessment was the use of study participants’ physical proximity to the wildfire event (e.g., “Wildfire exposure was defined as an area code location directly impacted by wildfire” [45]) (*n* = 22) [38, 42, 43, 45, 50, 57, 64, 67, 86–92, 95, 96, 100, 102, 105, 106]. Studies also defined exposure based on evacuation status (e.g., those who evacuated were considered exposed and those who did not evacuate were considered unexposed) (*n* = 6) [37, 82, 85, 93, 118, 119], and on estimated loss severity that was ascribed by the research team (e.g., “[Bushfire affectedness] was defined by evidence of high impact, operationalized as multiple fatalities and significant property loss; medium impact, defined by significant property damage and up to two fatalities; and low impact, where there was no evidence of burning” [71]) (*n* = 8) [70–75, 80, 107]. Two studies defined exposure as time periods that aligned with wildfire ignitions or smoke, respectively (*n* = 2) [94, 103]. One study developed a cumulative disaster exposure index to define the degree of regional exposure to disasters in 2020, using data on bushfire burns, droughts, floods or storms, COVID-19 infections, and pandemic-related job losses [46].

The remaining studies did not conduct a formal exposure assessment; rather, they considered all participants exposed (*n* = 18) [108–125]. For these studies, investigators either only included a sample of participants who indicated that they had been exposed to a wildfire (e.g., “Participants of interest were people who self-identified as affected by the recent bushfires” [111]) or exposure was assumed by the research team (e.g., only assessing mental health outcomes among a sample of evacuees from the Fort McMurray wildfire [116]). In the latter case, exposure to the wildfire was not explicitly measured. Among the exposed cohort studies, a few studies (*n* = 8) also assessed exposure with additional methods (i.e., questionnaire-derived, evacuation status, spatio-temporal proximity) [108, 110, 113, 114, 117–119, 125]. Nonetheless, these studies were ultimately included in the exposed cohort subset (Table 2), because they did not use the self-reported exposure metrics in conjunction with mental health outcomes.

### Mental Health Outcomes

Fourteen mental health outcomes were assessed in the studies identified by our literature search, and many studies investigated multiple outcomes (Fig. 3c). The most studied outcomes were depression, depressive symptoms, and major depressive disorder (MDD) (*n* = 51) [37, 41–43, 48–51, 53, 54, 56, 58–62, 64, 66, 70, 72–80, 85, 87–89, 92, 93, 98, 100, 103, 105, 107, 109, 111–119, 123, 124], post-traumatic stress, post-traumatic stress disorder (PTSD) and PTSD-like symptoms (*n* = 45) [37, 40, 47, 51, 53, 54, 56–64, 70, 72–80, 87, 90, 91, 93, 95–97, 99, 107, 109, 113–116, 118, 119, 121, 123–125], and anxiety, eco/climate-anxiety, and generalized anxiety disorder (GAD) (*n* = 41) [37, 39, 42, 43, 48–51, 54, 56, 58, 60–62, 64–66, 85, 86, 88, 89, 92, 98, 100–105, 107–115, 118, 123, 124]. Other investigated outcomes were stress/distress, anticipatory climate stress or adjustment disorder (*n* = 21) [48, 68, 70, 72, 75, 80, 82, 85, 88–90, 98, 100, 103–105, 111, 117, 118, 120, 122], substance use (*n* = 15) [43, 48, 52, 59, 62, 64, 66, 72, 73, 75, 80, 95, 100, 115, 120], resilience (*n* = 11) [40, 48, 54, 55, 62, 72, 75, 80, 109, 110, 115], suicide/suicidality (*n* = 11) [42, 45, 46, 58, 62, 64, 67, 106, 115, 118, 119], insomnia/sleeping problems (*n* = 10) [59, 105, 112–114, 116, 117, 121, 123, 124], self-esteem (*n* = 4) [42, 62, 64, 115], coping (*n* = 2) [82, 111], post-traumatic growth (*n* = 2) [81, 125], well-being (*n* = 2) [109, 119], paranoia (*n* = 2) [88, 89], and anger (*n* = 1) [71]. A few studies looked at overall mental health impacts, primarily via all-cause mental health-related healthcare utilization (*n* = 4) [38, 43, 46, 94]. In addition to these outcomes and their distributions across our literature sample, many studies listed additional mental health outcomes of interest. These, however, were not included in Fig. 3c if researchers did not directly assess their association with the wildfire exposure (e.g., severe mental illness in Cowlishaw et al. [71]). Some studies uniquely defined their outcome variables. In one, investigators used crisis texts that were coded by an algorithm with a crisis response tag (e.g., suicidal thoughts, self-harm, or depression) as proxies for mental health outcomes [45], and another defined perceived fire-related stress (measured by a scale developed by the research team) as the predictor variable in its association with the outcome of post traumatic growth [81].

### Outcome Measurement

Since we only included the mental health outcomes that were evaluated in association with wildfire exposure in Fig. 3c, similarly, we only included the outcome measurement tools employed for these specific outcomes Fig. 3d (see ‘Outcome Measurement’ in Table 1 and Table 2). All studies but seven used a standardized and validated scale to evaluate the mental health outcomes described above (Fig. 3d). For the other seven studies, one used REDCap survey data on self-rated health status, previous mental health diagnoses, and attribution of mental health symptoms to smoke [49]; one used a ‘fire-impact’ survey to assess self-reported mental health status and maintenance [85]; one used the previously described crisis text algorithm as a substitute for mental health outcome measurement [45]; one used prescription rates of psychotropic medications [105]; one calculated the defined daily doses of anxiolytics-hypnotics per 1,000 inhabitants per day [86]; one used county-level deaths by suicide [106]; and one used the response “anxiety” to the survey question “Did you have any of the following symptoms during or a few days after one of the smoke events in Summer 2023?” [108]. Notably, another study employed a battery of standardized scales such as those for depression, PTSD, and anxiety, but ultimately only used the self-reported question “Have you abused cannabis in the past year?” for their outcome measurement, as cannabis abuse was the only mental health outcome evaluated in association with wildfire exposure [52].

The most common tools employed to identify mental health outcomes were the Patient Health Questionnaire 9-item (PHQ-9) or PHQ-A (modified for adolescents) for depression (*n* = 28) [41, 51, 53, 54, 56, 58–62, 64, 70, 72, 74–80, 93, 109, 113–116, 123, 124], the PTSD Checklist (PCL-C [civilian], PCL-S [specific], and PCL-5 [Diagnostic and Statistical Manual of Mental Disorders, Fifth Edition]) for PTSD (*n* = 26) [40, 51, 53, 54, 56, 59–61, 63, 70, 72–80, 93, 109, 113, 114, 116, 123, 124], and the Generalized Anxiety Disorder 7-item (GAD-7) for anxiety (*n* = 14) [37, 39, 42, 51, 54, 56, 59–61, 65, 109, 113, 123, 124] (Fig. 3d). Other frequently used outcome measurement tools include the Kessler Psychological Distress Scale (K-6 [6-item] and K-10 [10-item]) (*n* = 8) [40, 68, 70, 72, 75, 80, 120, 122], the International Classification of Diseases, Ninth and Tenth Revisions (ICD-9/10) codes (*n* = 7) [38, 43, 46, 67, 94, 102, 121], the Insomnia Severity Index (ISI) (*n* = 6) [59, 113, 114, 116, 123, 124], the Brief Resilience Scale (BRS) (*n* = 6) [40, 48, 51, 54, 55, 109], the Alcohol Use Disorder Identification Test (AUDIT) (*n* = 6) [72, 73, 75, 80, 100, 120], the Impact of Events Scale-Revised (IES-R) and Children’s Revised IES (CRIES) (*n* = 6) [57, 87, 100, 107, 111, 125], the Athens Insomnia Scale (AIS) (*n* = 5) [59, 113, 114, 116, 121], the Hospital Anxiety and Depression Scale (HADS) (*n* = 5) [58, 62, 64, 66, 115], the Child PTSD Symptom Scale (CPSS) (*n* = 4) [58, 62, 64, 115], the Rosenberg Self-Esteem Scale (*n* = 4) [42, 62, 64, 115], the Depression Anxiety Stress Scale–21 items (DASS-21) (*n* = 3) [48, 103, 111], the CRAFFT Screening Tool for Adolescent Substance Abuse (CRAFFT) (*n* = 2) [62, 64], the Child and Youth Resilience Measure (CYRM-12) (*n* = 2) [62, 115], the State-Trait Anxiety Inventory (STAI) (*n* = 2) [99, 107], the Beck Depression Index (*n* = 2) [92, 107], the Mental Health Inventory-5 (MHI-5) (*n* = 2) [81, 82], the Post-Traumatic Growth Inventory (PTGI) and PTGI–Short Form (PTGI-SF) (*n* = 2) [81, 125], the Trauma Screening Questionnaire (TSQ) (*n* = 2) [95, 96], and the Post Traumatic Stress Disorder Reaction Index (PTSD-RI) (*n* = 2) [91, 97]. Measurement tools that were only used in single studies are not enumerated here but can be found in Table 2. 

### Statistical Methods

The most frequently employed statistical methods were univariate and multivariable regression (e.g., linear, logistic, mixed models) (*n* = 52) [37, 39, 42, 43, 46, 49–51, 53–57, 59, 61, 63, 65–68, 70–76, 80–82, 87, 89, 91–93, 95, 96, 98, 100, 102–104, 106, 112, 114, 116, 119–121, 123, 125], Chi-square tests (*n* = 16) [39–41, 49, 52–56, 60, 61, 63, 65, 97, 109, 114], ANOVA, ANCOVA and MANCOVA (*n* = 9) [48, 54, 85, 88, 90, 99, 107, 113, 124], t or z-tests (*n* = 7) [58, 94, 97, 99, 101, 109, 111], tests of correlation (*n* = 4) [48, 110, 111, 116], structural equation modeling (*n* = 4) [77–79, 118], permutation tests (*n* = 3) [62, 64, 115], and time series analyses (*n* = 4) [38, 45, 86, 105]. A portion of studies (24.0%) (*n* = 20) used multiple statistical methods to assess the association between wildfire exposure and adverse mental health outcomes. Among the studies with an explicitly defined population that was exposed to the wildfires (*n* = 67) (Table 1), 65.7% (*n* = 44) adjusted for confounding [37–39, 42, 43, 45, 46, 49–51, 53–55, 57, 58, 61, 63, 65, 66, 68, 70, 72–75, 77, 78, 80–82, 87, 89, 91–93, 95, 96, 98, 100, 102–107] and 19.4% (*n* = 13) formally evaluated effect modification [42, 43, 51, 58, 73, 77, 79, 80, 86, 92, 102, 105, 106]. In studies where everyone was considered exposed (*n* = 18) (Table 2), the investigators primarily assessed prevalence of the mental health outcomes after the event, and often assessed risk factors that might lead to a higher prevalence of adverse mental health outcomes following exposure. Among these studies, 44.4% (*n* = 8) evaluated risk factors [68, 112, 114–117, 121, 124].

### Mental Health Impacts

#### Depression

Depression (Table 4) was the most commonly assessed mental health outcome in our review. While most studies found that the prevalence of depression and depressive symptoms was higher than in the general population [171] after wildfire exposure, estimates varied widely, from 6.3% among survivors of low-affected areas 3–4 years after the Black Saturday bushfires [80] to 49.9% among Maui residents 5–13 months after the wildfire [42]. Only 41.2% of the studies that investigated depression evaluated it within a year of the associated wildfire event (*n* = 21) [37, 41, 42, 48–50, 66, 85, 87–89, 92, 93, 98, 103, 107, 111, 112, 116, 118, 119]. Specifically, direct fire exposure was linked to depression in Australian youth [48], Greek adults [88], Camp Fire survivors [51], Maui wildfire survivors [42], and Fort McMurray students, compared to those who did not have direct exposure to those fire events [62]. High wildfire exposure in Greek fire survivors [89] and high-fire affectedness during the Black Saturday bushfires [80] was also associated with depression, compared to low wildfire exposure and low-fire affectedness, respectively. Additionally, having to evacuate may be a relevant factor in mental health status after wildfire exposure. Among both Fort McMurray workers [66] and adults affected by the Witch Creek and Guejito fires [85], evacuating was significantly associated with depression, compared to those who did not evacuate. In another study among students who evacuated from the 1994 Sutherland bushfires, evacuation on the day of fires was associated with depression, while evacuating during the week of the fires was not [98]. Most studies (54.9%) employed a version of the PHQ to evaluate depression symptoms. Our review suggests that direct fire exposure, evacuating, and certain individual-level factors such as younger age, may be associated with higher risk of depression after wildfire exposure, with symptoms appearing to persist for many years after a wildfire event.

**Table 4:** Summary of mental health conditions assessed in association with wildfire exposure across reviewed literature

Mental health outcome(s)	Definition	Global prevalence
Depression, Depressive Symptoms, and MDD	Characterized by feelings of sadness, guilt, tiredness, hopelessness, low self-worth, loss of interest, and poor concentration [171]. MDD is defined by the lifetime absence of mania and the presence of a major depressive episode, which occurs when five depression symptoms are experienced during a 2-week period [172].	4.4% (depression) [171]
Anxiety and GAD	Characterized by feelings of worry, tension, and intrusive thoughts that are future-oriented, and may elicit physiological responses such as rapid heartbeat, sweating, and panic attacks [173]. GAD is defined by at least 6-months of these persistent symptoms, resulting in significant distress and/or impairment [174].	4.0% (anxiety) [175]
PTSD	Triggered by exposure to stressful events and is often characterized by intrusive memories of the trauma, distressing dreams, and flashbacks [176].	3.9% [177]
Psychological Stress or Distress	General terms that cover multiple conditions, including depression, anxiety, stress, or PTSD, and are linked to multiple negative health outcomes [178].	36% [179]
Substance Use	Characterized by the inability of a person to control their use of drugs, alcohol or medication despite negative effects [180].	2.2% [181]
Resilience	While there are many, evolving definitions of resilience [182], the American Psychological Association defines it as “the process and outcome of successfully adapting to difficult or challenging life experiences, especially through mental, emotional, and behavioral flexibility and adjustment to external and internal demands” [183]. In the context of wildfires, resilience can be thought of as the ability to psychologically respond to the disruptions and trauma caused by a wildfire, with relatively small sustained impacts to one’s mental health and well-being.	Highly variable depending on the study population [184, 185]
Insomnia	A sleep disorder characterized by having difficulty falling asleep, staying asleep, or getting good quality sleep [186].	10–30% [186]
Suicidality	Characterized by the risk of attempting suicide, which is indicated by suicidal thoughts, ideations, and/or plans [187].	9.9% (aggregate lifetime of suicidal plan) [188] 18% (aggregate lifetime of suicidal ideation) [188]
Self-Esteem	In simplistic terms, self-esteem encompasses how one views themself. However, it also serves as the foundation for other important mental constructs such as self-identity, self-image, and self-meaning [189].	Not applicable
Coping	In the context of wildfire exposure, coping can be defined as the way individuals manage their stress and distress levels following their experience of the adverse wildfire event [190].	Not applicable
Anger	While anger is a commonly felt emotion, “clinical anger” is defined by “heightened intensity, frequency, and duration of anger” which may lead to disruptions in work, relationships, and overall well-being [191].	Highly variable depending on the study population [192, 193]
Paranoia	“A condition characterized by delusions of persecution or grandiosity that are not as systematized and elaborate as in a delusional disorder or as bizarre as in paranoid schizophrenia” [194].	Highly variable depending on the study population and delusion type [195, 196]
Post-Traumatic Growth	“Positive psychological changes experienced as a result of the struggle with trauma or highly challenging situations” [197].	A meta-analysis showed that 52.3% of people who experienced trauma also experienced moderate-to-high post-traumatic growth [198].
Well-Being	“A positive state of emotional, psychological, and social health, characterized by a sense of contentment, resilience, and the ability to effectively cope with life’s challenges” [199].	Highly variable depending on the study population [200].

#### PTSD

Among studies included in this review, populations exposed to wildfires had substantially higher PTSD (Table 4) than the general population [177]. Research on the 2016 Fort McMurray wildfire found PTSD prevalence ranged from 10.2% [60] to 62.5% [116], varying by population and diagnostic tools. Interestingly, the prevalence of probable PTSD on the CPSS increased among Fort McMurray high school students from 37.0% in 2017 to 41.0% in 2019 [62, 115]. Another notable finding was that even five years after the fire, 39.6% of surveyed Fort McMurray residents met PTSD criteria on the PCL-C [53]. Studies of other wildfires, such as the 2019–2020 Black Summer bushfires, found PTSD prevalence as high as 58.7% on the eight-item PTSD Index (PTSD-8) [201] among highly exposed individuals [47], while the 2009 Black Saturday bushfires showed varying prevalences (15.6% for high-affected individuals and 1.0% for low on the PCL-S) [80]. Additionally, studies evaluating the relationship between wildfire exposure and PTSD revealed mixed associations between specific fire-related variables and PTSD risk. In Fort McMurray, while null associations were reported between most of the wildfire exposure predictors and PTSD [53, 60], proximity to destroyed properties [56], subjective fear and witnessing destruction [59], and witnessing fires and home destruction [62] were significantly associated with PTSD. In studies of other wildfires, direct exposure [47, 51, 75, 80], fear [72, 76, 97, 121], evacuation [37, 96], and loss [47, 51, 72, 74–76, 80, 91, 99] may be key factors of wildfire exposure that influence the development of PTSD, although the associations vary across contexts and populations. Interestingly, in a cross-sectional study of wildfire survivors from Australia, Canada, and the US, PTSD prevalence was consistently the most elevated of the mental health conditions evaluated (PTSD, anxiety, depression, and insomnia), with 48.6% of respondents in Australia, 75.0% in Canada, and 88.9% in the US having PTSD symptoms above the PCL-5 clinical threshold [124].

#### Anxiety

According to our review, anxiety (Table 4) prevalence following wildfire exposure showed substantial variation across events and populations, ranging from 14.2% (GAD-7) of Fort McMurray evacuees [59] to 86.2% (self-report) of older adults exposed to the Black Summer bushfires [110]. These older adults also rated the bushfires as a greater stressor than the COVID-19 pandemic [110]. In Fort McMurray, 31.0% of exposed high school students experienced probable anxiety in 2019 (HADS) [115], an increase from 27.0% in 2017 [62]. Fort McMurray adults 18 months post-fire reported a prevalence of 18.0% [61], while school district staff reported 15.7% [60], both on the GAD-7. Elsewhere, anxiety prevalence was 50.0% among Gangwon fire survivors [112], and medium-to-high wildfire exposure was associated with elevated anxiolytic drug consumption in Spain municipalities, compared to municipalities with no fire exposure [86]. Again, key wildfire exposure variables, such as direct exposure [48, 51, 62, 88, 89, 100, 107], fear [59, 61, 63, 65], and evacuation [66, 85] were associated with anxiety and anxiety-like symptoms, although one study of the 2025 Los Angeles wildfires did not find an association between evacuation status and anxiety [37]. Wildfire smoke was also uniquely associated with anxiety outcomes. Exposure to medium-to-heavy smoke from the 2018 Oregon wildfires for six or more weeks was associated with a 30.0% increase in difficulty controlling worrying, compared to those with four or less weeks of smoke exposure [50]. Another study identified an association between a 10 µg/m^3^ increase in 48-hour exposure to wildfire smoke PM_2.5_ and the risk of anxiety-related emergency department visits in the Western US [102]. Additionally, 45.3% of adults self-reported anxiety symptoms as a result of the smoke from the Black Summer bushfires [49]. Overall, the literature indicates heterogeneity in the anxiety prevalence following wildfire exposure, reflecting differences in the studied populations, leveraged measurement tools, and exposure contexts. 

#### Psychological Stress and Distress

Twenty-one studies evaluated psychological stress and distress (Table 4) in relation to wildfire exposure and all found some evidence of an association, although population prevalence was often not reported [48, 68, 70, 72, 75, 80, 82, 85, 88–90, 98, 100, 103–105, 111, 117, 118, 120, 122]. One year after the Wallow fire in Arizona, 35.0% of impacted respondents reported moderate or high levels of psychological distress, with higher distress among those with lower income and who experienced more negative financial effects from the fire [68]. Conversely, prevalence of severe psychological distress ranged from 4.4% to 9.8% 3–4 years after the Black Saturday bushfires, with the highest prevalence reported in highly affected communities [72, 80]. A longitudinal study of adults affected by the Black Saturday bushfires found that the prevalence of adjustment disorder increased at 10 years post-fire, compared to the prevalence identified 3–4 years post-fire [70]. In California, evacuees of the Gap/Tea/Jesusita fires and Witch Creek/Guejito fires consistently reported higher levels of distress compared to non-evacuees, including those with comorbid psychiatric conditions [82, 85]. Adults who experienced the 2007 Greek forest fires had significantly higher overall psychological distress scores six months after the fire compared to those living in nearby, unexposed areas [88, 89]. Interestingly, none of the Fort McMurray studies investigated stress or distress as a mental health endpoint. Together, these findings suggest that wildfire exposure is likely associated with stress or distress, but the magnitude of this impact likely depends on the wildfire context, as well as the timing of data collection post-fire.

#### Substance Use

Across 15 studies, substance use (Table 4) prevalence was generally elevated in populations exposed to wildfires, though heterogeneity in substances measured, populations studied, and assessment tools complicates direct comparisons [43, 48, 52, 59, 62, 64, 66, 72, 73, 75, 80, 95, 100, 115, 120]. Studies that incorporated a comparison (i.e., unexposed) population yielded particularly meaningful results. For instance, probable alcohol or substance use disorder was significantly more prevalent among adolescents who personally witnessed the Fort McMurray wildfire (16.0%) or whose home was destroyed (22.0%) compared to those who did not experience these events (9.0% and 14.0% respectively, CRAFFT) [62]. Another study of Fort McMurray adults found higher prevalence of substance use disorder among those who evacuated compared to the general Canadian population (7.9% to 4.4%, CAGE) [59]. There was no significant difference in heavy drinking among Australian adults highly impacted by the Black Saturday bushfires compared to those in low-impacted communities (AUDIT-C) [75], while Australian youth who reported being directly affected by the Black Summer bushfires had higher mean scores on a substance use screener compared to those not directly affected (1.85 to 1.40, UNCOPE) [48]. Among these adolescents, substance use scores were positively correlated with other mental health outcomes: depression, anxiety, stress, and adjustment disorder [48]. Uniquely, one study was able to compare tobacco use among adults before and after the Canberra Bushfires, finding that more fire-related experiences (e.g., evacuation, injury, property loss) and PTSD symptoms were associated with increased tobacco use from pre- to post-fire [95]. Collectively, these findings suggest that substance use-related associations with wildfire exposure may vary given the study context, the assessment tool, and individual-level and exposure-specific factors.

#### Resilience

Our search yielded eleven studies that explored resilience (Table 4) as a mental health outcome [40, 48, 54, 55, 62, 72, 75, 80, 109, 110, 115]. These studies generally suggest that individuals with greater wildfire impact tend to have lower resilience, but findings vary based on population and measurement tools. In a study of young adults affected by the Black Summer bushfires, low resilience scores were reported with the BRS [48]. Resilience was operationalized differently in studies of the Black Saturday bushfires, with resilience quantified as K-6 scores of 0–6 in the initial cohort study (77.1% prevalence) [80] and K-6 scores of 0–7 in the follow-up (81.8% prevalence) [75]. In Fort McMurray, resilience findings were mixed. The prevalence of low resilience five years after the fire was 37.4% (BRS), but wildfire exposure was not associated with resilience [55]. Relatedly, the prevalence of low resilience was 52.0% among residents of Alberta and Nova Scotia during the 2023 Canadian wildfires, but none of the evaluated wildfire exposure variables were associated with low resilience [40]. Among older adults living in communities affected by the 2020 Black Summer bushfires, those who reported being more impacted by the fires and the COVID-19 pandemic had lower rates of resilience [110]. Overall, resilience is not defined uniformly in assessments of mental health impacts following wildfire exposure and there is much variability in the prevalence of both low and high resilience across these communities.

#### Insomnia

The ten studies that assessed wildfire exposure in relation to insomnia (Table 4) reported relatively high post-fire prevalence of insomnia [59, 105, 112–114, 116, 117, 121, 123, 124], ranging from 13.5% among Fort McMurray evacuees [114] to 63.0% among victims of the Greek fires [121]. Another study on the same group of Fort McMurray evacuees found that insomnia was the most common mental health condition of those evaluated, with a prevalence of 28.5% [59]. This was also the only study that investigated wildfire exposures as risk factors for insomnia, ultimately finding varied results: seeing explosions or buildings collapsing, as well as getting separated from a loved one during the evacuation were positively associated with insomnia. All the other exposure-related predictors had null associations with insomnia [59]. While none of these studies had baseline, pre-fire estimates of insomnia, prevalence in the wildfire-exposed population was substantially higher than those among the general population [186].

#### Suicidality

Our review included eleven studies on suicidality (Table 4) and found limited evidence of association between wildfire exposure and suicidal thoughts [42, 45, 46, 58, 62, 64, 67, 106, 115, 118, 119]. Four of these studies examined past-month suicidal thoughts and lifetime suicide attempts among adolescents exposed to the Fort McMurray wildfire in 2016 [58, 62, 64, 115]. They found higher prevalence of suicidal thoughts among Fort McMurray students 18 months after the fire (16.0%) compared to non-Fort McMurray students (4.0%) [64], with prevalence increasing in the Fort McMurray students 3.5 years after the fire (18.0%) [115]. A quasi-experimental study using daily counts of texts to a crisis text line during three large California wildfires found no significant difference in the number of texts for suicidal thoughts during the wildfire period compared to non-wildfire periods [45]. While this study employed robust causal inference methods (i.e., interrupted time series and difference-in-differences analyses), they aggregated wildfire exposure at the telephone area code level, which may have resulted in exposure misclassification. Additionally, the concurrent COVID-19 pandemic likely impacted their estimates, as they could not distinguish between COVID-19 and wildfire impacts to the daily text counts. Another quasi-experimental study identified effect modification by rurality of the association between monthly smoke days and the number of suicides, with a positive association found only in rural US counties [106]. Given that a number of studies detected null associations [62, 67, 119], further research on wildfire exposure and suicidality and suicide is warranted.

#### Self-Esteem

We reviewed four studies that evaluated self-esteem (Table 4) as a mental health outcome. Three studies were conducted on students following the Fort McMurray wildfire and were performed by the same research group [62, 64, 115]. All studies used the Rosenberg Self-Esteem Scale. The first study found significantly lower self-esteem scores among Fort McMurray students–all considered exposed–18 months after the fire compared to non-Fort McMurray students [64]. Another study assessed the same Fort McMurray student population but classified students into exposure subgroups, finding that self-esteem scores were generally not different between exposure groups [62]. The final Fort McMurray study administered a survey once every three years and considered all students exposed [115]. Over time, self-esteem decreased and lower scores were observed in older students, female students, and students who were transgender or gender non-conforming [115]. Among Maui residents 5–13 months post-fire, the prevalence of low self-esteem was 26.1% [42]. These limited study populations and inconclusive findings on the association between wildfires and self-esteem highlight the need for further investigation.

#### Post-Traumatic Growth

Two studies explored post-traumatic growth (Table 4) in association with wildfire exposure [81, 125]. An online survey studying exposure to various Australian bushfires found that longer time since participants’ most recent bushfire experience was associated with decreased post-traumatic growth [125]. In contrast, a study determined that greater fire stress from the Gap, Tea and Jesusita fires among parent-youth dyads who had been evacuated was positively associated with post-traumatic growth [81]. These conflicting findings from two different wildfire contexts emphasize how post-traumatic growth has been understudied relative to wildfire exposure to date.

#### Coping

Our review identified two studies that investigated coping (Table 4) as an outcome. Afifi et al. evaluated “communal coping” among residents of the greater Santa Barbara, California area following the Gap, Tea, and Jesusita fires from 2008 to 2009 [82]. The authors defined communal coping as involving “groups of people confronting a shared stressor as a social unit,” and they found that those who were evacuated during the fires had a greater reliance on communal coping, as compared to those who were not evacuated [82]. Usher et al. studied community members affected by the 2019–2020 Black Summer bushfires, with all participants in this study considered exposed [111]. Participants reported use of both approach and avoidance coping strategies following their experience of the bushfires. Use of these coping strategies were positively correlated with symptoms of posttraumatic stress, depression, anxiety, and stress [111]. These findings highlight an important role of coping to address the mental health burdens experienced after a wildfire and support future investigations of coping as a relevant effect modifier.

#### Well-Being

Two studies investigated mental well-being (Table 4) as a mental health outcome relative to wildfire exposure. The first paper compared well-being among residents of Alberta and Nova Scotia during the 2023 Canadian wildfires before and after they enrolled in a supportive text message program [109]. Using the WHO-5, they found that the prevalence of poor mental well-being significantly decreased in the follow-up period compared to baseline, with none of the other evaluated mental health outcomes (i.e., anxiety, depression, PTSD, and resilience) significantly changing [109]. In contrast, a study of high school students affected by the 2010 Mount Carmel forest fire did not find a significant change in mental well-being at 6-months post-fire, compared to four weeks immediately following the fire, using the IDAS [119]. Despite the wildfire events occurring 13 years apart, these were the only two studies identified in our search that explored mental well-being in association with wildfire exposure, highlighting a need for further investigation.

#### Paranoia

Papanikolaou et al. investigated wildfire exposure and the experience of paranoia (Table 4) following the 2007 Greek forest fires [88, 89]. Those who were versus were not exposed had significantly higher paranoia scores on the SCL-90-R [88, 89]. Higher levels of paranoia were also observed among those who lost a close relative to the fires [89]. The authors described this study as a “cross sectional case control”, defining “cases” as those who were fire-exposed and “controls” as those who were unexposed.

#### Anger

One paper investigated anger (Table 4) as a mental health outcome of interest following the experience of a wildfire [71]. Among residents living in a high-level impact community following the Black Saturday bushfires, 10.0% met the DAR-5 criteria for significant anger problems [71]. The high bushfire impact group had 3.3 times the odds of having significant anger problems as compared to the low-to-moderate bushfire impact groups [71]. Additionally, anger symptoms tended to co-occur in individuals who were also experiencing other mental health conditions, such as PTSD and depression [71]. Anger remains understudied in association with wildfire exposure compared to other mental health endpoints identified in our review.

### Subgroup Findings

Of the 67 studies with an explicitly defined population that was exposed to the wildfires (Table 1), 19.4% formally evaluated effect modification through stratified analyses or the inclusion of interaction terms in regression models [42, 43, 51, 58, 73, 77, 79, 80, 86, 92, 102, 105, 106]. Given this small sub-sample, there was generally little agreement between studies on the influence of modifying factors. Age was explored as an effect modifier in four studies. While Jung et al. found that youth (ages 15-24) had increased emergency department visits for mood affective disorders during periods of elevated wildfire PM_2.5_ [43], Zhu et al. found more nuanced effects on anxiety-related emergency department visits for younger and older age groups, with both positive and negative associations identified for subgroups dependent on sex and how wildfire smoke exposure was modeled [102]. Six studies evaluated biological sex in subgroup analyses. Bryant et al. (2014) found outcome-specific modifying effects of sex: women were more likely to develop PTSD linked to the Black Saturday bushfires, while men were more likely to engage in heavy drinking, but there were comparable prevalences of resilience and depression among both sexes [80]. Additionally, a study of the 2006 Spain forest fires found increases in consumption of anxiolytics-hypnotics drugs only among men [86]. In contrast, a study of large wildfires in California from 2011 to 2018 identified that increases in psychotropic prescriptions in the postfire period were disproportionately higher among females compared to males [105]. Gender was also evaluated as an effect modifier in three other studies, with results showing gender playing both significant [73, 77] and non-significant [79] modifying roles. Two studies evaluated race and ethnicity as effect modifiers. Higher risks of emergency department visits for mood-affective disorders were identified in non-Hispanic Black individuals versus other racial and ethnic groups following short-term wildfire PM_2.5_ exposure in California [43], while suicide rates were higher among non-Hispanic white individuals versus other racial and ethnic groups in rural US counties in association with wildfire smoke days [106]. Previous trauma was another effect modifier investigated across studies. Pazderka et al. found that school-aged Fort McMurray adolescents with significant pre-existing trauma had higher levels of depression and PTSD, but not anxiety [58]. However, in their assessment of the Camp Fire, Silveira et al. did not identify a significant interaction between fire exposure and childhood trauma in exacerbating PTSD, depression, or anxiety symptoms [51]. While resilience was a protective factor in some studies, such that individuals with higher levels of resilience had less adverse mental health after the wildfire event [ 62], it was not formally evaluated as an effect modifier. Other effect modifiers that were explored were educational attainment [106], social support [42], rurality [106], and fire-related cognitions [92], often significantly modifying the wildfire exposure and mental health relationship. Overall, the small proportion of studies formally evaluating effect modification in this sample highlights the need for more robust subgroup analyses of the wildfire–mental health association, which can aid in the identification of relevant susceptibility and vulnerability factors.

Of the 18 studies in which everyone was considered exposed (Table 2), 44.4% evaluated risk factors for higher prevalence of adverse mental health outcomes after the wildfire events [68, 112, 114–117, 121, 124]. Age was evaluated as a risk factor, with studies finding disparate results. In some studies, older adults [121] or older students (as compared to younger students) [115] had worse mental health status post-fire exposure, while another study found that younger adults had more severe symptomology [116]. An analysis of both sex and gender identity found that female and transgender and gender non-conforming students exhibited higher mental health distress 3.5 years after the Fort McMurray wildfire [115]. Generally, studies found females had greater risk of poor mental health after wildfire exposure, as compared to males [114, 121, 124]. In a unique longitudinal study that used a household-level rapid needs assessment to evaluate disaster recovery from the 2011 Bastrop County Complex Wildfire, residing in homes that sustained damage in 2011 was a risk factor for elevated stress levels, feelings of depression/hopelessness, and sleeping problems in 2015 [117]. Collectively, these results highlight that many risk factors may influence mental health prevalence after wildfire exposure, but more studies are needed to confirm the findings from this limited sample.

## Discussion

A growing body of literature finds a relationship between exposure to a wildfire event and increased risk of adverse mental health outcomes. Our review spanned 85 studies published from 2002 to 2025 that evaluated 25 unique wildfires or combined wildfire events. Most of these investigations found elevated prevalences of mental health conditions, as compared to global estimates, following wildfire exposure. While many different mental health outcomes were evaluated, depression, PTSD, and anxiety were the most commonly investigated. Among the studies with exposed-only populations (*n* = 18), poor mental health was highly prevalent, with over half of the study population reporting severe outcomes like insomnia and PTSD in some investigations. These exposed-only studies also identified factors that increased the risk of poor mental health following wildfire exposure, such as older age, non-male sex, and income. Studies in which exposure was explicitly measured (*n* = 67), the prevalence of poor mental health after wildfire exposure was again elevated as compared to global estimates, but these findings were more variable than in the exposed-only assessments. A number of wildfire-specific exposures were significantly associated with mental health outcomes, including directly experiencing the fire, witnessing burning of homes or structures, and having to evacuate. We identified a few themes as major limitations in this area of research, including a limited geographic scope as evidenced by studies evaluating only 25 total wildfire events, the shortage of longitudinal data with pre-wildfire health status data, and the lack of appropriate comparison populations. We provide a critical evaluation of the current literature and the remaining limitations, with suggestions for improved future research efforts.

While we identified studies conducted on wildfires across the globe, their geographic distribution is not representative of the patterns observed in global wildfire events. From 1979 to 2019, the Mediterranean, southern Amazonia, east Siberia, southeast Australia, Pacific US, Pacific Canada, and Alaska have experienced the greatest increases in the length of fire season [202]. Since lengthened fire seasons strongly correlate with area burned, these areas have thus experienced more frequent and intense wildfires [202]. We identified a substantial number of studies from areas with heightened fire activity (i.e., 30.6% of studies from southeast Australia, 16.5% from the Pacific US, 5.9% from the Greek Mediterranean, and 2.4% from Pacific Canada), but other regions were not represented, such as Amazonia or Siberia. The majority of Canadian wildfire studies were conducted on the Fort McMurray wildfire in Alberta, Canada (70.4%). While Canada has experienced significant wildfire events in the 21st century, with over 6,000 fires and 15 million hectares burned in 2023 [130], the Fort McMurray wildfire generated a disproportionate number of studies. Researchers should conduct studies across broader geographies, especially locations with increased wildfires but no investigations, and consider evaluating multiple wildfire events simultaneously; for example, all wildfires in California, such as done by Jung et al. who evaluated associations between wildfire PM_2.5_ and mental health emergency department visits from July-December 2020 [43].

Only 25 wildfires or combined wildfire events were evaluated across this study sample. We did not identify consistent characteristics of wildfires, such as acreage burned or number of fatalities, that produced more investigation of their mental health impacts. Rather, we found that a regionally proximate research team engaged in this work was the strongest factor in predicting study generation. This raises the concern that the mental health impacts of wildfires occurring in areas without a nearby research team to evaluate them are likely underestimated or not estimated at all. For example, the Camp Fire, which destroyed 95% of the structures in the towns of Paradise and Concow and claimed the lives of 85 people [203], is the deadliest wildfire in California history to date, and yet, only one study [51] investigated its impact on mental health, finding a significantly increased risk for PTSD and depression among those who had direct exposure to the fire. A more systematic approach and local, regional, and international collaborations are needed to thoroughly investigate wildfires that may produce the most adverse mental health impacts among affected populations, identify suitable comparison groups, and ensure that actionable research is being conducted.

Most studies (57.6%) identified in our review were cross-sectional. These studies are useful in providing a snapshot of the prevalence of mental health conditions present within a fire-affected community at the time of evaluation, but they cannot provide insight into temporality of the association or trends over time, limiting their ability to yield causal evidence. Another limitation is that the timing of data collection after the fire event was inconsistent. Whereas some data collections began within a month of the wildfire [66, 112], others did not occur until five years later [53, 55]. A limited number of investigations in our sample were truly longitudinal in study design, with studies conducting, at most, two follow-ups of data collection. While it is important to understand potential long-term impacts, we cannot robustly evaluate how these trends change over time without longitudinal studies on the same population. The lack of pre-wildfire health data in most places substantially limits our ability to fully characterize the association between wildfire exposure and mental health. Collecting time series and longitudinal data is necessary to evaluate mental health trends and the role of mediating and modifying factors, such as resilience, after wildfire events. These findings highlight a need for further longitudinal investigations into the mental health impacts of wildfires, as well as for studies that assess the acute mental health effects shortly after the wildfires.

### Exposure Assessment

Assessment of wildfire exposure was largely non-uniform and non-standardized across all the reviewed studies. The methods employed to quantify and assign exposure included the use of questionnaires, evacuation status, spatio-temporal proximity, and estimated severity of loss from the fire. Exposure was not explicitly defined in 21.2% of our studies, with no exposure assessment taking place and all participants being considered exposed. While there is currently no gold standard for exposure assessment of wildfires, there are both benefits and limitations to each method identified in our study sample. Quantitative-based methods, such as chemical transport models and satellite-based predictions of wildfire-related air pollution, have been well-established in investigations of wildfire exposure and physical health outcomes. These wildfire smoke models estimate exposure at the area-level (e.g., Childs et al. predicting wildfire PM_2.5_ concentrations at a 10km^2^ grid [44]), making them susceptible to exposure misclassification, especially when individual-level behaviors or locations are not tracked. Wildfire smoke models also only capture a single facet of wildfire exposure [204–206]. Given that most people spend the majority of their time indoors (e.g., Americans are indoors nearly 90% of the time [207]), a comprehensive wildfire exposure assessment should include indoor air quality data; however, no studies in our review used indoor air measurements or data on where their study populations spent most of their time. Furthermore, because wildfires produce multiple potentially hazardous exposures, researchers may wish to consider factors like wildfire PM_2.5_ alongside stressors like evacuation to more holistically capture the wildfire experience. No studies considered pathways via other environmental pollutants, including ozone, volatile organic chemicals, metals, or water pollution, which could plausibly influence acute and chronic mental health outcomes. This is a major limitation of the current literature, as the biological mechanisms underpinning the wildfire–mental health relationship remain largely under-characterized, although neuroinflammatory and oxidative changes from PM_2.5_ have been proposed [208, 209]. Hence, there is a pressing need for future toxicologic, physiologic, and exposure assessment investigations to elucidate how, on a biological level, aspects of wildfire exposure manifest in adverse mental health outcomes.

Questionnaires can aid in providing exposure estimates when monitoring data are lacking or unavailable. Quantifying wildfire exposure with a self-questionnaire may be limited by selection or recall bias, but it can provide valuable insight into individual-level factors that may strongly influence mental health, such as loss of residence or displacement after the fire. Some studies on the Fort McMurray wildfire distributed the “Impact of Fire Questionnaire (IOF)” [62], which was developed by the research team and used to measure exposure-relevant variables, such as participants’ location during the fire and whether they personally saw the fire. Despite the IOF being developed internally by the Fort McMurray research team, it was not used uniformly in all of their investigations, limiting cross-study comparisons. Given the unique experience of mental health conditions across individuals and wildfire contexts, detailed exposure assessments that include questionnaire data should be employed when inference is being made at the individual-level.

### Outcomes and Measurement Tools

The ongoing global mental health crisis is a major public health challenge, with the United Nations expressing concern over how it was exacerbated by the COVID-19 pandemic [210]. Climate change is also expected to worsen the mental health crisis, with conditions like climate anxiety [211] arising and acute events such as wildfires directly impacting mental health [212]. Our review of the existing literature supports that a community’s mental health is adversely impacted by exposure to a wildfire event, but the evidence to date is not robust with limited ability to draw causal conclusions. While an array of mental health outcomes was evaluated, such as insomnia, stress/distress, and substance use disorder, the conditions of depression, PTSD, and anxiety were the most studied.

#### Depression

The majority of studies (58.5%) in our sample assessed depression and depressive symptoms among wildfire-affected communities, reporting a wide range of depression prevalence (6.3% [80] – 45.0% [53]). Wildfire exposure may exacerbate pre-existing depression, or it could trigger the onset of new depressive symptoms, and this mechanism may be driven by the type and severity of exposure. For instance, qualitative data from a rural Washington community indicate that prolonged wildfire smoke events exacerbate existing depression and that elevated depression is experienced around and during wildfire smoke exposure [213]. Experiencing depression after a wildfire may be particularly tied to the loss of one’s home, belongings, livelihood, or sense of security after a disaster. Depression may also be fueled by solastalgia, a feeling of distress caused by environmental change, particularly driven by climate change [214]. This type of distress can lead to feelings of hopelessness and despair, further exacerbating depressive tendencies. Evidence for an association between depression and climate-driven extreme events is supported by research on other disasters. For instance, one study revealed that patients at a psychiatric outpatient clinic in Jackson, Mississippi had significantly higher depressive symptom scores one month after Hurricane Katrina than they had prior to the hurricane [215]. This was predicted by time spent without electricity and watching footage of looting in New Orleans on television [215]. While some of the studies in our sample evaluated similar predictors (e.g., the frequency of watching television about the wildfire devastation), they did not find significant associations with depression and related symptoms [53, 60, 61]. Further investigation is needed to comprehensively determine which dimensions of wildfire exposure are the most influential in the development or exacerbation of depression.

Regarding outcome measurement, the studies in our sample used multiple methods, including the PHQ-9, HADS, and DASS-21. While these screening tools are well validated in multiple contexts, especially in clinical settings, studies of acute disaster impacts may wish to consider using shorter, easier to administer scales. For example, the PHQ-2 can be completed in less than a minute and has somewhat higher sensitivity (albeit slightly lower specificity) than the PHQ-9 [216]. Since very few studies in our sample evaluated depression and other outcomes in the immediate aftermath of wildfires, the PHQ-2 was understandably not employed, but concurrently, our understanding of these acute impacts is very limited. To improve efficiency and reduce the burden on sampled communities, future studies of wildfire exposure and acute depression impacts may consider using the PHQ-2 and other shorter scales.

#### PTSD

PTSD and PTSD-like symptoms were frequently studied in the current literature, with 52.9% of studies evaluating these outcomes, finding a prevalence ranging from 1.0% [80] to 88.9% [124] in post-wildfire exposure. PTSD develops following exposure to a stressful event and manifests through disruptions to the body’s biochemical and hormonal pathways, often triggering the fight or flight response at inappropriate times [217]. PTSD has clear diagnostic criteria and validated screening tools, making it one of the most commonly studied health effects in the aftermath of disasters [218]. For instance, a meta-analysis on the PTSD prevalence among survivors after a typhoon or hurricane revealed a combined prevalence of 17.8%, with increasing prevalence related to the storm intensity [219, 220]. Unlike some other mental health conditions, the DSM-5 criteria of PTSD is directly linked to a specific traumatic event (i.e., threatened death, serious injury, or sexual violence) [176]. While this allows us to identify an explicit causal pathway with wildfire exposures, the narrow definition of traumatic events may fail to capture the totality of stressors that can nonetheless lead to PTSD. Rather, a more inclusive definition of trauma, centered on experiences (e.g., financial distress, loss, climate-related hazards) instead of events, has been proposed [221]. Such a definition may have greater utility in identifying cases of PTSD that result from less extreme, but still trauma-inducing, experiences, such as prolonged wildfire smoke or media about wildfire evacuations in nearby areas.

Furthermore, PTSD symptoms and risk factors are similar to those of other mental health conditions, such as anxiety and depression [222]. This can result in studies evaluating PTSD-like symptoms, rather than attempting to fully diagnose PTSD among wildfire victims. In the absence of an experiences-centered definition of trauma for PTSD, prioritizing the identification of PTSD-like symptoms in wildfire-exposed communities, rather than formally diagnosing PTSD, may be more beneficial for swift treatment and continued care. This implicates which screening or diagnostic tools are employed after the wildfire. In our review, PTSD and related symptoms were most often evaluated through iterations of the PTSD Checklist (PCL) (e.g., the PCL-C [civilian] or PCL-S [specific]), although other measures such as the Child PTSD Symptom Scale (CPSS) were also leveraged. The PCL is a screening tool that assesses symptom severity but does not provide a PTSD diagnosis [223]. Thus, researchers may want to administer the PCL to evaluate acute PTSD-like symptoms soon after a wildfire when clinical resources are limited, but should consider following up with a formal diagnostic assessment, as PTSD prevalence from the PCL tools may be under-reported.

#### Anxiety

Generalized anxiety disorder and anxiety-like symptoms were investigated by 48.2% of reviewed studies, with a prevalence ranging from 14.2% [59] to 86.2% [110]. Anxiety disorders are the most common mental health condition, affecting up to 30% of adults at some point in their lifetimes [224]. Since anxiety may not necessarily be triggered by a singular event, it is more challenging to ascribe anxiety to a specific wildfire event than, for example, PTSD. This may complicate research efforts attempting to draw a causal link between wildfire exposure and anxiety, as there are many potential risk factors for anxiety-related outcomes. Furthermore, climate anxiety is a growing phenomenon in which people experience anxiety over climate change and, resultantly, their psychological well-being is negatively impacted [101, 104, 211]. Thus, as climate change increases the frequency and intensity of wildfires, individuals experiencing anxiety may have compounded risks. Children, adolescents, and younger adults disproportionately experience anxiety [225] and climate anxiety [211, 226, 227]. While our review of the literature suggests they may be particularly susceptible to poor mental health following wildfire exposure [55, 97, 116], only four studies of post-fire anxiety evaluated age as an effect modifier or risk factor [43, 102, 105, 115]. Brown et al. found that older students had worse anxiety 3.5 years after the Fort McMurray wildfire [115], while Wettstein et al. found significant increases in prescriptions of anxiolytics following the start of large California wildfires among adult groups [105]. Studies of wildfire smoke PM_2.5_ found conflicting modifying effects of age: older versus younger adults exhibited a stronger association between exposure in the western US from 2007 to 2018 and anxiety-related emergency department visits [102], while exposure in California in 2020 and anxiety-related emergency department visits was not modified by age [43]. These limited findings highlight a major gap in our understanding of how wildfire exposure may exacerbate an already elevated anxiety prevalence among younger generations. Future studies should aim to address this gap, while considering methods to account for how the experience of a wildfire may exacerbate climate anxiety in addition to general anxiety.

The collective finding in the majority of reviewed studies of elevated anxiety and associated symptoms post-wildfire is supported in other disaster contexts, though anxiety has not been as often studied in these contexts as depression and PTSD. While many studies of disasters tend to evaluate anxiety along with other conditions [228–230], anxiety is less frequently investigated as the only outcome of interest [141, 231]. This could be due to the relatively high prevalence of anxiety in the general population, which may complicate the establishment of appropriate control groups and confound associations between anxiety and disaster-specific exposures. Additionally, there is substantial overlap in the symptomology of anxiety and the other mental health outcomes identified through this search. Thus, while it is important to characterize anxiety-specific impacts of wildfires with tools such as the GAD-7 and HADS, which were most employed in our search, it may be most useful from a clinical perspective to evaluate anxiety alongside comorbid mental health conditions. Future research activities may consider initially leveraging more comprehensive mental health screening tools, such as the Mental Health Inventory [232], which identifies the risk of negative symptoms such as anxiety and depression while also evaluating positive emotional states such as life satisfaction.

#### Resilience

Resilience at both the individual and community levels is a critical indicator of good mental health, especially in the context of climate change-driven events [233]. Resiliency can mediate the intensity and duration of adverse mental health impacts. Many of the studies in our sample attempted to characterize resilience in individuals following their exposure to a wildfire event but lacked consistent definitions and measures of resiliency. Studies also reported disparate results [40, 62, 80], suggesting that the ability of individuals and communities to exhibit mental health resilience is likely contextually dependent on the wildfire events and support services that are available. Additionally, the limited longitudinal analyses highlight a need for future studies that follow affected individuals over time to better ascertain how their mental health needs and adaptive capacity may change. This is especially important to fully characterize resilience, which may continue to evolve for years after disasters such as wildfires. Ultimately, the current literature’s inconsistency in the definition, measurement, and findings of resilience following wildfire exposure limits broad conclusions and emphasizes a need for further research. Future efforts should be directed towards developing a standardized definition of resilience and determining which measurement tools are most appropriate for evaluation in wildfire-affected communities.

#### Other Mental Health Outcomes

We identified several other mental health outcomes that were evaluated in the literature following wildfire exposure. These include–in order of most frequently studied– psychological stress and distress, substance use, suicidality, insomnia, self-esteem, post-traumatic growth, coping, well-being, paranoia, and anger. Less than a quarter of studies explored these individual outcomes, with 17.9% of studies evaluating substance use and only one study evaluating anger. Thus, these outcomes appear to be substantially understudied in association with wildfire exposure, as compared to depression, PTSD, and anxiety. Our understanding of the wildfire–mental health relationship would greatly benefit from targeted investigation of these understudied outcomes. Fortunately, all of these outcomes have well-validated screening tools–such as the Kessler Psychological Distress Scale [234], Insomnia Severity Index [235], and the Coping Orientation to Problems Experienced Inventory [236]–which greatly aids in measuring these mental health impacts and facilitating cross-study comparisons. Furthermore, as shown by Lykins et al., these outcomes are highly correlated [48]. Thus, measuring these less studied outcomes alongside depression, PTSD, and anxiety will likely yield more comprehensive mental health assessments.

### The Wildfire Context

Comprehensive screening of all mental health outcomes should be conducted in wildfire affected communities to ultimately inform care and treatment. In fact, the US Substance Abuse and Mental Health Services Administration (SAMHSA) lists wildfires as a disaster that can cause particular emotional distress and it provides a unique Disaster Distress Helpline for those who are struggling after their wildfire exposure [237]. While the screening tools for mental health outcomes are well-validated in clinical settings, they may need further validation in disaster contexts, specifically for wildfires. For instance, the Post-Hurricane Distress Scale was developed to evaluate the risk of adversely impacted mental health following the experience of a hurricane [238]. A similar tool could be deployed in the aftermath of wildfires to assess acute mental health impacts and identify at-risk individuals in need of further outcome-specific evaluation. Relatedly, Liu et al. developed a library of questionnaires that can be readily deployed during and after wildfire events to evaluate health impacts [239]. From their systematic review of the literature, the greatest number of questionnaires were identified for mental health purposes [239]. This work both highlights the relevance of mental health impacts in wildfire contexts and provides an excellent consolidation of the assessment tools used for such purposes. Nonetheless, the growing evidence supporting these adverse mental health outcomes emphasizes the need for a coordinated, global response to reduce the barriers to mental health service utilization and to better support both individual and community coping strategies following traumatic wildfire events [240, 241].

### Epidemiologic Limitations

#### Confounding

One challenge in measuring associations between wildfire exposure and mental health outcomes is finding an appropriate comparison population who are similar to those affected by the wildfire, but who are less or not exposed. However, communities differ in many aspects, leading to potential confounding, or biased effect estimates due to other factors (not related to wildfires) that differ between the exposed and unexposed groups and that cause adverse mental health outcomes. Even when comparing outcomes associated with specific wildfire events within a single community, such as losing a home, there may be economic and social factors, such as poverty, that are associated with both the event of interest and mental health outcomes. If these factors are not considered there may be unmeasured confounding leading to biased results. Many of the populations included in this review were exposed to non-wildfire factors that may have impacted mental health outcomes. For example, Fort McMurray was experiencing economic decline before, during, and after the wildfire in 2016 which likely had a large impact on the mental health of its residents [242]. Comparing mental health outcomes of residents of Fort McMurray with residents of another city without accounting for this economic decline may exaggerate the association of wildfire exposure and mental health outcomes [58, 62, 64, 115]. Similarly, studies which compare high-affected communities and low-affected communities exposed to the same wildfire (i.e., the Black Saturday bushfires) without considering that high-affected communities may be more affected due to structural differences in income and poverty levels, which also contribute to mental health status, may also find biased associations [75, 80].

Overall, confounding is difficult to account for in estimating the association between wildfire exposure and mental health outcomes because unexposed comparison populations likely differ in ways not related to the exposure. Most reviewed studies account for this by limiting the causal statements they make; however, it is important to consider the impact of potential unmeasured confounding when interpreting associations present in these studies as well as to consider alternative explanations for the outcomes we observe. Additionally, future research may benefit from including rigorous observational designs such as case-crossover studies that inherently control for time-invariant confounders [102], or other longitudinal study designs.

#### Potential for Bias

Selection bias, or when the population of people included in studies is not representative of the target population, presents another challenge that may lead to biased estimates of association. Those most affected by wildfires may be unavailable to participate in studies or potentially more likely to agree to participate, with the latter scenario biasing effect estimates away from the null. Some studies included in our review, particularly those among adolescents in Fort McMurray, avoid this problem by sampling the entire population of interest, yielding a relatively high response rate (73.8% [62, 64, 115] – 96.5% [58]). Several other studies used random samples with lower response rates (16%–40% [59, 75]), which can increase the chance of selective response rates compared to full enumeration. Other studies, however, used convenience samples drawn from online recruiting [48], which comes with a higher risk of selection bias compared to random sampling. Finally, longitudinal studies that begin with limited sampling bias may accrue bias over time if those most exposed tend to drop out sooner. Overall, researchers should consider the potential for selection bias when studying wildfires, especially when direct exposure, property loss, or evacuation occurred, and take steps to minimize bias during data collection, analysis, and reporting of results.

#### Generalizability

Overall, the reviewed literature on wildfire exposure and adverse mental health outcomes has limited generalizability. Our search yielded 85 quantitative studies, but not every study evaluated a range of mental health outcomes and study locations were geographically confined, spanning just eight countries and 25 unique wildfire events. For comparison, the National Interagency Coordination Center identified 891 large wildland fires in the US in 2023 alone [243]. Hence, the current literature does not estimate the collective burden of wildfires on the mental health of affected communities. Among affected populations, children and younger adult populations were the most frequently evaluated in the literature, with only one study focused on older adults’ post-fire experiences [110]. Thus, we have a better understanding of some mental health impacts (i.e., depression, PTSD, and anxiety) over others (e.g., psychological distress, insomnia, and substance use disorder) among specific communities in a few fire-affected areas. Future investigations should refrain from studying wildfire events and mental health outcomes in isolation; rather, they should holistically characterize mental health status across multiple wildfire events to ascertain their population-attributable burden on mental health.

While previous large-scale studies on multiple wildfires have conducted analyses on mortality [244] or anemia [245], very few investigations have evaluated the population risk of mental health. Using secondary data sources, like claims or electronic health records, could improve study generalizability by including more people who do not have to directly opt in to research [246]. Indeed, several studies identified in this review analyzed healthcare utilization data to investigate the relationship between wildfire PM_2.5_ and mental health, finding significant associations for anxiety-related emergency department visits [102], all-cause mental health-related emergency department visits [43], and outpatient neuropsychiatric visits [38]. Such datasets do, however, require people to seek care for mental health concerns, which likely make them unrepresentative of the full exposed population. To address this limitation, researchers may leverage electronic health records from specific populations that have frequent clinical check-ups, such as pregnant people. This may allow for the comparison of mental health status before and after wildfire events. Other data options include Google searches, which may be relevant to diverse wildfire contexts and do not require people to seek care for researchers to track mental health trends [247].

### Response

Our search yielded only three quantitative studies that focused on interventions to prevent adverse mental health outcomes following wildfire exposure [109, 113, 123]. Thus, the majority of quantitative work on wildfires and mental health to date is descriptive. Future research activities are needed on solutions-oriented work to investigate how to better respond to the mental health concerns that have already been identified. For instance, a study that conducted key informant interviews among a community affected by wildfires in Alaska empowered individuals to identify the most actionable and impactful interventions to support wildfire-related mental health [248]. These included having equitable access to information, campaigns/programs connecting citizens to local mental healthcare systems, and mental health debriefing at evacuation sites [248]. Such findings highlight the importance of qualitative work on the association between wildfire exposure and mental health outcomes. Our initial search identified 32 qualitative studies, but these were beyond the scope of this review. This is a limitation of our work, as we likely did not consider much of the recovery and response-focused literature on the wildfire–mental health relationship.

Mental health care response is further complicated for individuals residing in rural areas, where there is a lack of mental health services and higher reluctance to seek care, especially in older, veteran populations [249]. Researchers working in such contexts may need to spend additional time and effort to build trust with these communities. Relatedly, all research activities on wildfires must take additional care to exercise ethical practices and engagements with affected communities that may be working through post-disaster recovery. In addition to possibly struggling with poor mental health after wildfire exposure, individuals may also be dealing with household smoke damage, resettling after having to evacuate, or relocating entirely after home and property loss. Therefore, studies conducted among wildfire-affected communities must be driven by ethical research practices and should, when applicable, prioritize actionable findings that align with the communities’ mental health and recovery needs.

### Limitations of This Work

This review has notable limitations. First, PubMed, Web of Science, and Google Scholar were selected as the literature search databases given their accessibility and breadth of content focus. Including more databases (e.g., Embase, Scopus) in our search would likely have expanded the number of identified studies and potentially led us to different conclusions. Another major limitation is that a single author performed the primary charting (i.e., data extraction) process. While all authors reviewed the extracted data and a second reviewer adjudicated many of the studies, having a secondary reviewer perform a complimentary charting process may have enhanced the consistency of results. Additionally, as previously discussed, our focus on quantitative studies precluded the review of qualitative and grey literature, which may have encompassed most of the community-driven and post-wildfire recovery work.

## Conclusion

As wildfires continue to increase in frequency and intensity with climate change, researchers must expand their activities to better understand which wildfire attributes, health conditions, individual and community-level characteristics, and community resources may ultimately buffer against adverse mental health outcomes following wildfire exposure. With improved uniformity in how wildfire exposure is assessed and mental health outcomes are measured, public and mental health professionals may better identify at-risk communities and develop targeted interventions for support, coping, and resiliency building. Specifically, we recommend the following priority areas for future research on the association between wildfire exposure and mental health outcomes to address:

1. Investigations of the wildfire–mental health association in areas previously understudied but at high wildfire risk under future climate change scenarios, such as regions in South America or British Columbia, Canada.
2. Clear definitions of pathways meant to be captured by wildfire exposure assessment and standardized exposure assessment across geographic and wildfire contexts to improve comparability between studies.
3. Steps taken to minimize confounding bias (including relevant comparison populations) and selection bias (both in sample selection and statistical methodology).
4. Where ethical, evaluation of acute mental health impacts in the time immediately after a wildfire, as well as the utilization of longitudinal studies to assess changes to mental health status over time to establish the duration of impact following wildfire exposure.
5. Exploration of innovative uses of pre-existing datasets to alleviate survey burdens among highly exposed communities.
6. The use of robust observational study designs that may more adequately adjust for confounding, such as longitudinal cohorts or case-crossover studies.
7. Further evaluation of mediating (e.g., property loss) and modifying (e.g., resilience and the level of support at the community, state/municipality, and federal levels) factors.
8. When possible, the pursuit of actionable research informed by the communities’ needs.

## Data Availability

No datasets were generated or analysed during the current study.

## References

[1] CoreLogic. Corelogic estimates the eaton and palisades fires are causing devastating initial property losses estimated to be between 35 billion to 45 billion. Cotality 2025. (accessed April 1, 2025). (accessed April 1, 2025). https://www.cotality.com/press-releases/corelogic-estimates-the-eaton-and-palisades-fires-are-causing-devastating-initial-property-losses-estimated-to-be-between-35-billion-to-45-billion

[2] McConnell K, Whitaker S, Fussell E, DeWaard J, Price K, Curtis K. Effects of wildfire destruction on migration, consumer credit, and financial distress 2021. 10.2139/ssrn.3995455

[3] Smith AB. U.S. billion-dollar weather and climate disasters, 1980 - present 2020. 10.25921/STKW-7W73

[4] Radeloff VC, Hammer RB, Stewart SI, Fried JS, Holcomb SS, McKeefry JF. The wildland–urban interface in the United States. Ecol Appl. 2005;15:799–805.

[5] Roos D. Native Americans used fire to protect and cultivate land. HISTORY. 2020. (accessed July 20, 2022). (accessed July 20, 2022). https://www.history.com/news/native-american-wildfires

[6] Reardon-Smith M. accessed December 1,. Aboriginal burning practices meet colonial legacies in Australia n.d. (2023). https://edgeeffects.net/aboriginal-burning-australia/

[7] Wagtendonk V, Jan W. The history and evolution of wildland fire use. Fire Ecol. 2007;3:3–17.

[8] Dodd B. Civil liability: prescribed burning operations: gross negligence. 2021.

[9] Xu R, Yu P, Abramson MJ, Johnston FH, Samet JM, Bell ML, et al. Wildfires, global climate change, and human health. N Engl J Med. 2020;383:2173–81. 33034960 10.1056/NEJMsr2028985

[10] Ellis TM, Bowman DMJS, Jain P, Flannigan MD, Williamson GJ. Global increase in wildfire risk due to climate-driven declines in fuel moisture. Glob Chang Biol. 2022;28:1544–59. 34800319 10.1111/gcb.16006

[11] Canadell JG, Meyer CPM, Cook GD, Dowdy A, Briggs PR, Knauer J, et al. Multi-decadal increase of forest burned area in Australia is linked to climate change. Nat Commun. 2021;12:6921. 34836974 10.1038/s41467-021-27225-4 PMC8626427

[12] Fire Statistics. Canadian interagency forest fire centre Inc 2023. (accessed August 16, 2023). https://ciffc.net/statistics

[13] Congressional research service. Wildfire statistics 2022.

[14] Marris E. Hawaii wildfires: did scientists expect Maui to burn? Nat Publishing Group UK. 2023. 10.1038/d41586-023-02571-z. 10.1038/d41586-023-02571-z 37580550

[15] Benmarhnia T, Errett NA, Casey JA. Beneath the smoke: Understanding the public health impacts of the Los Angeles urban wildfires. Environ Epidemiol. 2025;9:e388. 40304010 10.1097/EE9.0000000000000388 PMC12040033

[16] Barnes C, … Arrighi J. Climate change increased the likelihood of wildfire disaster in highly exposed Los Angeles area – World Weather Attribution. World Weather Attribution 2025. (accessed February 20, 2025). https://www.worldweatherattribution.org/climate-change-increased-the-likelihood-of-wildfire-disaster-in-highly-exposed-los-angeles-area/

[17] Burke M, Driscoll A, Heft-Neal S, Xue J, Burney J, Wara M. The changing risk and burden of wildfire in the United States. Proc Natl Acad Sci U S A. 2021;118. 10.1073/pnas.2011048118. 10.1073/pnas.2011048118 PMC7812759 33431571

[18] Radeloff VC, Helmers DP, Kramer HA, Mockrin MH, Alexandre PM, Bar-Massada A, et al. Rapid growth of the US wildland-urban interface raises wildfire risk. Proc Natl Acad Sci U S A. 2018;115:3314–9. 29531054 10.1073/pnas.1718850115 PMC5879688

[19] Thomas AS, Escobedo FJ, Sloggy MR, Sánchez JJ. A burning issue: Reviewing the socio-demographic and environmental justice aspects of the wildfire literature. PLoS ONE. 2022;17:e0271019. 35900980 10.1371/journal.pone.0271019 PMC9333234

[20] Auer MR, Hexamer BE. Income and insurability as factors in wildfire risk. Forests. 2022;13:1130.

[21] Adachi JK, Li L. The impact of wildfire on property prices: An analysis of the 2015 Sampson Flat Bushfire in South Australia. Cities. 2023;136:104255.

[22] Dong H. Climate change and real estate markets: An empirical study of the impacts of wildfires on home values in California. Landsc Urban Plan. 2024;247:105062.

[23] Thompson JJ, Wilby RL, Hillier JK, Connell R, Saville GR. Climate gentrification: Valuing perceived climate risks in property prices. Ann Am Assoc Geogr. 2023;113:1092–111.

[24] Lambrou N, Kolden C, Loukaitou-Sideris A. Disaster recovery gentrification in post-wildfire landscapes: The case of Paradise, CA. Int J Disaster Risk Reduct. 2025;118:105235.

[25] Absher JD, Vaske JJ. Modelling public support for wildland fire policy. Sustainable forestry: from monitoring and modelling to knowledge management and policy science. UK: CABI; 2007. pp. 159–70.

[26] Davies IP, Haugo RD, Robertson JC, Levin PS. The unequal vulnerability of communities of color to wildfire. PLoS ONE. 2018;13:e0205825. 30388129 10.1371/journal.pone.0205825 PMC6214520

[27] Conlisk E, Butsic V, Syphard AD, Evans S, Jennings M. Evidence of increasing wildfire damage with decreasing property price in Southern California fires. PLoS ONE. 2024;19:e0300346. 38656930 10.1371/journal.pone.0300346 PMC11042721

[28] Reid CE, Brauer M, Johnston FH, Jerrett M, Balmes JR, Elliott CT. Critical review of health impacts of wildfire smoke exposure. Environ Health Perspect. 2016;124:1334–43. 27082891 10.1289/ehp.1409277 PMC5010409

[29] Mental Health. APA dictionary of psychology 2018. (accessed February 7, 2026). https://dictionary.apa.org/

[30] Arias D, Saxena S, Verguet S. Quantifying the global burden of mental disorders and their economic value. EClinicalMedicine. 2022;54:101675. 36193171 10.1016/j.eclinm.2022.101675 PMC9526145

[31] Scieszka D, Hunter R, Begay J, Bitsui M, Lin Y, Galewsky J, et al. Neuroinflammatory and neurometabolomic consequences from inhaled wildfire smoke-derived particulate matter in the western United States. Toxicol Sci. 2022;186:149–62. 34865172 10.1093/toxsci/kfab147 PMC8883349

[32] Chu B, Marwaha K, Sanvictores T, Awosika AO, Ayers D, Physiology. stress reaction. StatPearls, Treasure Island (FL): StatPearls Publishing; 2025. 31082164

[33] Isaac F, Toukhsati SR, Di Benedetto M, Kennedy GA. A systematic review of the impact of wildfires on sleep disturbances. Int J Environ Res Public Health. 2021;18:10152. 34639453 10.3390/ijerph181910152 PMC8508521

[34] Berry HL, Bowen K, Kjellstrom T. Climate change and mental health: a causal pathways framework. Int J Public Health. 2010;55:123–32. 20033251 10.1007/s00038-009-0112-0

[35] Eisenman DP, Galway LP. The mental health and well-being effects of wildfire smoke: a scoping review. BMC Public Health. 2022;22:2274. 36471306 10.1186/s12889-022-14662-z PMC9724257

[36] To P, Eboreime E, Agyapong VIO. The impact of wildfires on mental health: A scoping review. Behav Sci. 2021;11. 10.3390/bs11090126. 10.3390/bs11090126 PMC8466569 34562964

[37] Lee R, Unger JB, Soto DW, Kawaguchi E, Cockburn M, Paul S, et al. Depression, anxiety, and PTSD among Southern California residents after the January 2025 Los Angeles wildfires. Disaster Med Public Health Prep. 2025;19:e327. 41292207 10.1017/dmp.2025.10257 PMC13520775

[38] Casey JA, Gu YM, Schwarz L, Frankland TB, Wilner LB, McBrien H, et al. The 2025 Los Angeles wildfires and outpatient acute health care utilization. JAMA Health Forum. 2025;6:e254632. 41296376 10.1001/jamahealthforum.2025.4632 PMC12658665

[39] Obuobi-Donkor G, Shalaby R, Agyapong B, da Luz Dias R, Agyapong VIO. 2023 wildfires in Canada: Living in wildfire regions in Alberta and Nova Scotia doubled the odds for residents to experience likely Generalized Anxiety Disorder symptoms. J Clin Med. 2024;13:3234. 38892945 10.3390/jcm13113234 PMC11172488

[40] Adu MK, Shalaby R, Agyapong B, Dias Rda, Agyapong L. Exploring the prevalence and predictors of low resilience and likely PTSD in residents of two provinces in Canada during the 2023 wildfires. Front Public Health. 2024;12:1343399. 38590805 10.3389/fpubh.2024.1343399 PMC10999595

[41] Mao W, Shalaby R, Agyapong B, Obuobi-Donkor G, Da Luz Dias R, Agyapong VIO. Devastating wildfires and mental health: Major depressive disorder prevalence and associated factors among residents in Alberta and Nova Scotia, Canada. Behav Sci (Basel). 2024;14:209. 38540512 10.3390/bs14030209 PMC10967904

[42] Juarez R, Phankitnirundorn K, Ozorio Dutra SV, Bond-Smith D, Lee AG, Maunakea AK. Health and social support in the aftermath of the Maui wildfires. JAMA Netw Open. 2025;8:e2525430. 40768146 10.1001/jamanetworkopen.2025.25430 PMC12329611

[43] Jung YS, Johnson MM, Burke M, Heft-Neal S, Bondy ML, Chinthrajah RS, et al. Fine particulate matter from 2020 California wildfires and mental health-related emergency department visits. JAMA Netw Open. 2025;8:e253326. 40184065 10.1001/jamanetworkopen.2025.3326 PMC11971671

[44] Childs ML, Li J, Wen J, Heft-Neal S, Driscoll A, Wang S, et al. Daily local-level estimates of ambient wildfire smoke PM2.5 for the contiguous US. Environ Sci Technol. 2022;56:13607–21. 36134580 10.1021/acs.est.2c02934

[45] Sugg MM, Runkle JD, Hajnos SN, Green S, Michael KD. Understanding the concurrent risk of mental health and dangerous wildfire events in the COVID-19 pandemic. Sci Total Environ. 2022;806:150391. 34844328 10.1016/j.scitotenv.2021.150391 PMC8455091

[46] Sanatkar S, Harvey SB, Mackinnon A, Bryant R, Sara G. Emergency department mental health presentations in bushfire-, flood-, storm-, drought-, and COVID-19-affected areas: Analysis of growth models between 2017 and 2021. Aust N Z J Public Health. 2025;49:100251. 40499338 10.1016/j.anzjph.2025.100251

[47] Cruwys T, Macleod E, Heffernan T, Walker I, Stanley SK, Kurz T, et al. Social group connections support mental health following wildfire. Soc Psychiatry Psychiatr Epidemiol. 2023. 10.1007/s00127-023-02519-8. 37428193 10.1007/s00127-023-02519-8 PMC11116249

[48] Lykins AD, Parsons M, Craig BM, Cosh SM, Hine DW, Murray C. Australian youth mental health and climate change concern after the black summer bushfires. EcoHealth. 2023;20:3–8. 37115466 10.1007/s10393-023-01630-1 PMC10141828

[49] Rodney RM, Swaminathan A, Calear AL, Christensen BK, Lal A, Lane J, et al. Physical and mental health effects of bushfire and smoke in the Australian capital territory 2019-20. Front Public Health. 2021;9:682402. 34722432 10.3389/fpubh.2021.682402 PMC8551801

[50] Mirabelli MC, Vaidyanathan A, Pennington AF, Ye D, Trenga CA. Wildfire smoke and symptoms affecting mental health among adults in the U.S. state of Oregon. Prev Med. 2022;164:107333. 36336164 10.1016/j.ypmed.2022.107333 PMC9691586

[51] Silveira S, Kornbluh M, Withers MC, Grennan G, Ramanathan V, Mishra J. Chronic mental health sequelae of climate change extremes: A case study of the deadliest Californian wildfire. Int J Environ Res Public Health 2021;18(4):1487. 10.3390/ijerph18041487 PMC7915298 33557397

[52] Obuobi-Donkor G, Eboreime E, Shalaby R, Agyapong B, Agyapong VIO. Prevalence and correlates of cannabis abuse among residents in the community of Fort McMurray, a city in Northern Alberta which had endured multiple natural disasters. Front Psychiatry. 2022;13:962169. 36213902 10.3389/fpsyt.2022.962169 PMC9533067

[53] Mao W, Adu M, Eboreime E, Shalaby R, Nkire N, Agyapong B et al. Post-traumatic stress disorder, major depressive disorder, and wildfires: A fifth-year postdisaster evaluation among residents of fort McMurray. Int J Environ Res Public Health 2022;19(15):9759. 10.3390/ijerph19159759 PMC9368448 35955114

[54] Agyapong B, Shalaby R, Eboreime E, Obuobi-Donkor G, Owusu E, Adu MK, et al. Cumulative trauma from multiple natural disasters increases mental health burden on residents of Fort McMurray. Eur J Psychotraumatol. 2022;13:2059999. 35599978 10.1080/20008198.2022.2059999 PMC9116266

[55] Adu MK, Eboreime E, Shalaby R, Sapara A, Agyapong B, Obuobi-Donkor G, et al. Five years after the fort McMurray wildfire: Prevalence and correlates of low resilience. Behav Sci. 2022;12. 10.3390/bs12040096. 10.3390/bs12040096 PMC9024963 35447668

[56] Ritchie A, Sautner B, Omege J, Denga E, Nwaka B, Akinjise I, et al. Long-term mental health effects of a devastating wildfire are amplified by sociodemographic and clinical antecedents in college students. Disaster Med Public Health Prep. 2021;15:707–17. 32536354 10.1017/dmp.2020.87

[57] Verstraeten BSE, Elgbeili G, Hyde A, King S, Olson DM. Maternal mental health after a wildfire: Effects of social support in the fort McMurray wood buffalo study. Can J Psychiatry. 2021;66:710–8. 33172310 10.1177/0706743720970859 PMC8320544

[58] Pazderka H, Brown MRG, Agyapong VIO, Greenshaw AJ, McDonald-Harker CB, Noble S, et al. Collective trauma and mental health in adolescents: A retrospective cohort study of the effects of retraumatization. Front Psychiatry. 2021;12:682041. 34248717 10.3389/fpsyt.2021.682041 PMC8267583

[59] Belleville G, Ouellet M-C, Lebel J, Ghosh S, Morin CM, Bouchard S, et al. Psychological symptoms among evacuees from the 2016 fort McMurray wildfires: A population-based survey one year later. Front Public Health. 2021;9:655357. 34017813 10.3389/fpubh.2021.655357 PMC8130827

[60] Agyapong VIO, Ritchie A, Brown MRG, Noble S, Mankowsi M, Denga E, et al. Long-term mental health effects of a devastating wildfire are amplified by socio-demographic and clinical antecedents in elementary and high school staff. Front Psychiatry. 2020;11:448. 32528323 10.3389/fpsyt.2020.00448 PMC7265240

[61] Moosavi S, Nwaka B, Akinjise I, Corbett SE, Chue P, Greenshaw AJ, et al. Mental health effects in primary care patients 18 months after a major wildfire in fort McMurray: Risk increased by social demographic issues, clinical antecedents, and degree of fire exposure. Front Psychiatry. 2019;10:683. 31620033 10.3389/fpsyt.2019.00683 PMC6760025

[62] Brown MRG, Agyapong V, Greenshaw AJ, Cribben I, Brett-MacLean P, Drolet J, et al. Significant PTSD and other mental health effects present 18 months after the fort Mcmurray wildfire: Findings from 3,070 grades 7–12 students. Front Psychiatry. 2019;10:623. 31543839 10.3389/fpsyt.2019.00623 PMC6728415

[63] Agyapong VIO, Juhas M, Omege J, Denga E, Nwaka B, Akinjise I, et al. Prevalence rates and correlates of likely post-traumatic stress disorder in residents of fort McMurray 6 months after a wildfire. Int J Ment Health Addict. 2019;19:632–50.

[64] Brown MRG, Agyapong V, Greenshaw AJ, Cribben I, Brett-MacLean P, Drolet J, et al. After the Fort McMurray wildfire there are significant increases in mental health symptoms in grade 7–12 students compared to controls. BMC Psychiatry. 2019;19:18. 30630501 10.1186/s12888-018-2007-1 PMC6329184

[65] Agyapong VIO, Hrabok M, Juhas M, Omeje J, Denga E, Nwaka B, et al. Prevalence rates and predictors of generalized anxiety disorder symptoms in residents of Fort McMurray six months after a wildfire. Front Psychiatry. 2018;9:345. 30108527 10.3389/fpsyt.2018.00345 PMC6079280

[66] Cherry N, Haynes W. Effects of the Fort McMurray wildfires on the health of evacuated workers: follow-up of 2 cohorts. CMAJ Open. 2017;5:E638–45. 28819065 10.9778/cmajo.20170047 PMC5621945

[67] Scales SE, Camphausen LC, Horney JA, Kintziger KW. Violent deaths following disasters: A retrospective analysis. PLoS ONE. 2025;20:e0337968. 41343446 10.1371/journal.pone.0337968 PMC12677568

[68] Eisenman D, McCaffrey S, Donatello I, Marshal G. An Ecosystems and vulnerable populations perspective on solastalgia and psychological distress after a wildfire. EcoHealth. 2015;12:602–10. 26302957 10.1007/s10393-015-1052-1

[69] Higginbotham N, Connor L, Albrecht G, Freeman S, Agho K. Validation of an environmental distress scale. EcoHealth. 2007;3:245–54.

[70] Pacella BJ, Cowlishaw S, Gibbs L, Bryant RA, Brady K, Gallagher C, et al. Trajectory of adjustment difficulties following disaster: 10-year longitudinal cohort study. BJPsych Open. 2024;10:e57. 38433588 10.1192/bjo.2024.3 PMC10951843

[71] Cowlishaw S, Metcalf O, Varker T, Stone C, Molyneaux R, Gibbs L, et al. Anger dimensions and mental health following a disaster: Distribution and implications after a major bushfire. J Trauma Stress. 2021;34:46–55. 33136348 10.1002/jts.22616

[72] Bryant RA, Gibbs L, Colin Gallagher H, Pattison P, Lusher D, MacDougall C, et al. The dynamic course of psychological outcomes following the Victorian Black Saturday bushfires. Aust N Z J Psychiatry. 2021;55:666–77. 33176436 10.1177/0004867420969815

[73] Molyneaux R, Gibbs L, Bryant RA, Humphreys C, Hegarty K, Kellett C, et al. Interpersonal violence and mental health outcomes following disaster. BJPsych Open. 2019;6:e1. 31796146 10.1192/bjo.2019.82 PMC7001465

[74] Gallagher HC, Block K, Gibbs L, Forbes D, Lusher D, Molyneaux R, et al. The effect of group involvement on post-disaster mental health: A longitudinal multilevel analysis. Soc Sci Med. 2019;220:167–75. 30447481 10.1016/j.socscimed.2018.11.006

[75] Bryant RA, Gibbs L, Gallagher HC, Pattison P, Lusher D, MacDougall C, et al. Longitudinal study of changing psychological outcomes following the Victorian Black Saturday bushfires. Aust N Z J Psychiatry. 2018;52:542–51. 28605987 10.1177/0004867417714337

[76] Bryant RA, Gallagher HC, Gibbs L, Pattison P, MacDougall C, Harms L, et al. Mental health and social networks after disaster. Am J Psychiatry. 2017;174:277–85. 27838935 10.1176/appi.ajp.2016.15111403

[77] Gallagher HC, Lusher D, Gibbs L, Pattison P, Forbes D, Block K, et al. Dyadic effects of attachment on mental health: Couples in a postdisaster context. J Fam Psychol. 2017;31:192–202. 27869452 10.1037/fam0000256

[78] Gallagher HC, Richardson J, Forbes D, Harms L, Gibbs L, Alkemade N, et al. Mental health following separation in a disaster: The role of attachment: Disaster-related separation and attachment. J Trauma Stress. 2016;29:56–64. 26749321 10.1002/jts.22071

[79] Forbes D, Alkemade N, Waters E, Gibbs L, Gallagher C, Pattison P, et al. The role of anger and ongoing stressors in mental health following a natural disaster. Aust N Z J Psychiatry. 2015;49:706–13. 25586750 10.1177/0004867414565478

[80] Bryant RA, Waters E, Gibbs L, Gallagher HC, Pattison P, Lusher D, et al. Psychological outcomes following the Victorian Black Saturday bushfires. Aust N Z J Psychiatry. 2014;48:634–43. 24852323 10.1177/0004867414534476

[81] Felix E, Afifi T, Kia-Keating M, Brown L, Afifi W, Reyes G. Family functioning and posttraumatic growth among parents and youth following wildfire disasters. Am J Orthopsychiatry. 2015;85:191–200. 25822609 10.1037/ort0000054

[82] Afifi WA, Felix ED, Afifi TD. The impact of uncertainty and communal coping on mental health following natural disasters. Anxiety Stress Coping. 2012;25:329–47. 21801075 10.1080/10615806.2011.603048

[83] Afifi TD, Hutchinson S, Krouse S. Toward a theoretical model of communal coping in postdivorce families and other naturally occurring groups. Commun Theory. 2006;16:378–409.

[84] Afifi WA, Weiner JL. Seeking information about sexual health: Applying the theory of motivated information management. Hum Commun Res. 2006;32:35–57.

[85] Tally S, Levack A, Sarkin AJ, Gilmer T, Groessl EJ. The impact of the San Diego wildfires on a general mental health population residing in evacuation areas. Adm Policy Ment Health. 2013;40:348–54. 22665076 10.1007/s10488-012-0425-9

[86] Caamano-Isorna F, Figueiras A, Sastre I, Montes-Martínez A, Taracido M, Piñeiro-Lamas M. Respiratory and mental health effects of wildfires: an ecological study in Galician municipalities (north-west Spain). Environ Health. 2011;10:48. 21600035 10.1186/1476-069X-10-48 PMC3224540

[87] Papadatou D, Giannopoulou I, Bitsakou P, Bellali T, Talias MA, Tselepi K. Adolescents’ reactions after a wildfire disaster in Greece. J Trauma Stress. 2012;25:57–63. 22298431 10.1002/jts.21656

[88] Papanikolaou V, Leon GR, Kyriopoulos J, Levett J, Pallis E. Surveying the ashes: experience from the 2007 Peloponnese wildfires six months after the disaster. Prehosp Disaster Med. 2011;26:79–89. 21888727 10.1017/S1049023X11000094

[89] Papanikolaou V, Adamis D, Mellon RC, Prodromitis G. Psychological distress following wildfires disaster in a rural part of Greece: a case-control population-based study. Int J Emerg Ment Health. 2011;13:11–26. 21957753

[90] Mellon RC, Papanikolau V, Prodromitis G. Locus of control and psychopathology in relation to levels of trauma and loss: self-reports of Peloponnesian wildfire survivors: Locus of Control and Psychopathology in Relation to Levels of Trauma and Loss. J Trauma Stress. 2009;22:189–96. 19452533 10.1002/jts.20411

[91] Yelland C, Robinson P, Lock C, La Greca AM, Kokegei B, Ridgway V, et al. Bushfire impact on youth. J Trauma Stress. 2010;23:274–7. 20419736 10.1002/jts.20521

[92] Scher CD, Ellwanger J. Fire-related cognitions moderate the impact of risk factors on adjustment following wildfire disaster. J Anxiety Disord. 2009;23:891–6. 19574024 10.1016/j.janxdis.2009.05.007

[93] Marshall GN, Schell TL, Elliott MN, Rayburn NR, Jaycox LH. Psychiatric disorders among adults seeking emergency disaster assistance after a wildland-urban interface fire. Psychiatr Serv. 2007;58:509–14. 17412853 10.1176/ps.2007.58.4.509

[94] Moore D, Copes R, Fisk R, Joy R, Chan K, Brauer M. Population health effects of air quality changes due to forest fires in British Columbia in 2003: Estimates from physician-visit billing data. Can J Public Health. 2006;97:105–8. 16619995 10.1007/BF03405325 PMC6975989

[95] Parslow RA, Jorm AF. Tobacco use after experiencing a major natural disaster: analysis of a longitudinal study of 2063 young adults. Addiction. 2006;101:1044–50. 16771896 10.1111/j.1360-0443.2006.01481.x

[96] Parslow RA, Jorm AF, Christensen H. Associations of pre-trauma attributes and trauma exposure with screening positive for PTSD: analysis of a community-based study of 2,085 young adults. Psychol Med. 2006;36:387–95. 16255836 10.1017/S0033291705006306

[97] McDermott BM, Lee EM, Judd M, Gibbon P. Posttraumatic stress disorder and general psychopathology in children and adolescents following a wildfire disaster. Can J Psychiatry. 2005;50:137–43. 15830823 10.1177/070674370505000302

[98] McDermott BM, Palmer LJ. Postdisaster emotional distress, depression and event-related variables: findings across child and adolescent developmental stages. Aust N Z J Psychiatry. 2002;36:754–61. 12406117 10.1046/j.1440-1614.2002.01090.x

[99] Jones RT, Ribbe DP, Cunningham PB, Weddle JD, Langley AK. Psychological impact of fire disaster on children and their parents. Behav Modif. 2002;26:163–86. 11961911 10.1177/0145445502026002003

[100] McFarlane AC, Van Hooff M. Impact of childhood exposure to a natural disaster on adult mental health: 20-year longitudinal follow-up study. Br J Psychiatry. 2009;195:142–8. 19648546 10.1192/bjp.bp.108.054270

[101] Mosca A, Luciani D, Chiappini S, Miuli A, PsyClimate R, Group, Cianconi P, et al. Eco-anxiety and mental health: Correlates of climate change distress. Int J Environ Res Public Health. 2025;22:1768. 41464402 10.3390/ijerph22121768 PMC12732531

[102] Zhu Q, Zhang D, Wang W, D’Souza RR, Zhang H, Yang B, et al. Wildfires are associated with increased emergency department visits for anxiety disorders in the western United States. Nat Ment Health. 2024;2:379–87. 39568497 10.1038/s44220-024-00210-8 PMC11575985

[103] Giles LV, Thomson CJ, Lesser I, Brandenburg JP. Running through the haze: How wildfire smoke affects physical activity and mental well-being. J Phys Act Health. 2024;21:1435–45. 39504954 10.1123/jpah.2024-0305

[104] Tao TJ, Estes KD, Holman EA, Vahedifard F, Silver RC. Understanding climate change anxiety and anticipatory climate disaster stress: A survey of residents in a high-risk California county during wildfire season. BMJ Ment Health. 2025;28:e301331. 40389304 10.1136/bmjment-2024-301331 PMC12104908

[105] Wettstein ZS, Vaidyanathan A. Psychotropic medication prescriptions and large California wildfires. JAMA Netw Open. 2024;7:e2356466. 38407907 10.1001/jamanetworkopen.2023.56466 PMC10897744

[106] Molitor D, Mullins JT, White C. Air pollution and suicide in rural and urban America: Evidence from wildfire smoke. Proc Natl Acad Sci U S A. 2023;120:e2221621120. 37695917 10.1073/pnas.2221621120 PMC10515164

[107] Jones RT, Ribbe DP, Cunningham P, Weddle JD. Psychosocial correlates of wildfire disaster: Post disaster adult reactions. Fire Technol. 2003;39:103–17.

[108] Barrera EI, Hayden A, Meredith G, Noel CA. Perceptions of and responses to wildfire smoke among New York State residents: A cross-sectional study. Int J Environ Res Public Health. 2025;22:277. 40003502 10.3390/ijerph22020277 PMC11855130

[109] Obuobi-Donkor G, Shalaby R, Agyapong B, Dias Rda, Eboreime L, Wozney E. Evaluating the 3-month post-intervention impact of a supportive text message program on mental health outcomes during the 2023 wildfires in Alberta and Nova Scotia, Canada. Front Public Health. 2024;12:1452872. 39744361 10.3389/fpubh.2024.1452872 PMC11688365

[110] Halcomb E, Thompson C, Morris D, James S, Dilworth T, Haynes K, et al. Impacts of the 2019/20 bushfires and COVID-19 pandemic on the physical and mental health of older Australians: a cross-sectional survey. Fam Pract. 2023;40:449–57. 36462177 10.1093/fampra/cmac138 PMC10231380

[111] Usher K, Durkin J, Douglas L, Coffey Y, Bhullar N. Coping styles and mental health outcomes of community members affected by black summer 2019-20 bushfires in Australia. Int J Ment Health Nurs. 2022;31:1176–85. 35731685 10.1111/inm.13035

[112] Hong JS, Hyun SY, Lee JH, Sim M. Mental health effects of the Gangwon wildfires. BMC Public Health. 2022;22:1183. 35701801 10.1186/s12889-022-13560-8 PMC9195206

[113] Belleville G, Ouellet M-C, Békés V, Lebel J, Morin CM, Bouchard S, et al. Efficacy of a Therapist-Assisted Self-Help Internet-Based Intervention Targeting PTSD, Depression, and Insomnia Symptoms After a Disaster: A Randomized Controlled Trial. Behav Ther. 2023;54:230–46. 36858756 10.1016/j.beth.2022.08.004

[114] Binet É, Ouellet M-C, Lebel J, Békés V, Morin CM, Bergeron N, et al. A portrait of mental health services utilization and perceived barriers to care in men and women evacuated during the 2016 Fort McMurray wildfires. Adm Policy Ment Health. 2021;48:1006–18. 33641027 10.1007/s10488-021-01114-w PMC7914389

[115] Brown MRG, Pazderka H, Agyapong VIO, Greenshaw AJ, Cribben I, Brett-MacLean P, et al. Mental health symptoms unexpectedly increased in students aged 11–19 years during the 3.5 years after the 2016 Fort McMurray wildfire: Findings from 9,376 survey responses. Front Psychiatry. 2021;12:676256. 34093284 10.3389/fpsyt.2021.676256 PMC8172807

[116] Belleville G, Ouellet M-C, Morin CM. Post-traumatic stress among evacuees from the 2016 Fort McMurray wildfires: Exploration of psychological and sleep symptoms three months after the evacuation. Int J Environ Res Public Health. 2019;16. 10.3390/ijerph16091604. 10.3390/ijerph16091604 PMC6540600 31071909

[117] Kirsch KR, Feldt BA, Zane DF, Haywood T, Jones RW, Horney JA. Longitudinal community assessment for public health emergency response to wildfire, Bastrop County, Texas. Health Secur. 2016;14:93–104. 27081889 10.1089/hs.2015.0060

[118] Hashoul-Andary R, Assayag-Nitzan Y, Yuval K, Aderka IM, Litz B, Bernstein A. A longitudinal study of emotional distress intolerance and psychopathology following exposure to a potentially traumatic event in a community sample. Cognit Ther Res. 2016;40:1–13.

[119] Zeller M, Yuval K, Nitzan-Assayag Y, Bernstein A. Self-compassion in recovery following potentially traumatic stress: longitudinal study of at-risk youth. J Abnorm Child Psychol. 2015;43:645–53. 25234347 10.1007/s10802-014-9937-y

[120] Wasiak J, Mahar P, Lee S, Paul E, Spinks A, Pfitzer B, et al. 12-month generic health status and psychological distress outcomes following an Australian natural disaster experience: 2009 Black Saturday Wildfires. Injury. 2013;44:1443–7. 23021367 10.1016/j.injury.2012.08.060

[121] Psarros C, Theleritis C, Economou M, Tzavara C, Kioulos KT, Mantonakis L, et al. Insomnia and PTSD one month after wildfires: evidence for an independent role of the fear of imminent death. Int J Psychiatry Clin Pract. 2017;21:137–41. 28084115 10.1080/13651501.2016.1276192

[122] Camilleri P, Healy C, Macdonald E, Nicholls S, Sykes J, Winkworth G, et al. Recovery from bushfires: The experience of the 2003 Canberra bushfires three years after. Australas J Paramed. 2010;8:1–15.

[123] Isaac F, Klein B, Nguyen H, Watson S, Kennedy GA. Digital cognitive behavioral therapy-based treatment for insomnia, nightmares, and posttraumatic stress disorder symptoms in survivors of wildfires: Pilot randomized feasibility trial. JMIR Hum Factors. 2025;12:e65228. 40085843 10.2196/65228 PMC11953604

[124] Isaac F, Toukhsati SR, Klein B, Di Benedetto M, Kennedy GA. Differences in anxiety, insomnia, and trauma symptoms in wildfire survivors from Australia, Canada, and the United States of America. Int J Environ Res Public Health. 2023;21:38. 38248503 10.3390/ijerph21010038 PMC10815777

[125] Hooper J, Magor-Blatch L, Bhullar N. Life after bushfire: Post-traumatic stress, coping, post-traumatic growth. Australas J Paramed. 2018;15:1–11.

[126] Saleh F. accessed March 31,. One year after the 2025 Los Angeles fires: What has changed, what have we learned, and what actions must still happen? Prevention Web 2026. https://www.preventionweb.net/news/one-year-after-2025-los-angeles-fires-what-has-changed-what-have-we-learned-and-what-actions

[127] 2025 Fire Season Incident Archive. CAL FIRE n.d. (accessed March 15, 2026). https://www.fire.ca.gov/incidents/2025

[128] Leonard M. The Maui Wildfires Were Massive. These numbers help us comprehend them. Honolulu civil beat 2024. (accessed March 31, 2026) https://www.civilbeat.org/2024/08/the-maui-wildfires-were-massive-these-numbers-help-us-comprehend-them/

[129] Preliminary After-Action Report. 2023 Maui Wildfire. US fire administration n.d. (accessed March 31, 2026). https://www.usfa.fema.gov/blog/preliminary-after-action-report-2023-maui-wildfire/

[130] Natural Resources Canada. Canada’s record-breaking wildfires in 2023: A fiery wake-up call 2023. (accessed January 10, 2025). https://natural-resources.canada.ca/stories/simply-science/canada-s-record-breaking-wildfires-2023-fiery-wake-call

[131] Cecco L. Canada’s fires are getting fiercer – and rebuilding is becoming a challenge. The Guardian 2023.

[132] Canada wildfires, 2023 - Forensic analysis 2024. (accessed March 31, 2026) https://www.undrr.org/resource/canada-wildfires-2023-forensic-analysis

[133] 2020 Fire Season Incident Archive. CAL FIRE n.d. / (accessed March 31, 2026). https://www.fire.ca.gov/incidents/2020

[134] Recovery Collection. Australia: Black summer bushfires 2019–2020 2022. (accessed September 28, 2024). https://recovery.preventionweb.net/collections/recovery-collection-australia-black-summer-bushfires-2019-2020

[135] About. accessed September 28, : Goseong Fire of 2019. DBpedia n.d. (2024). http://dbpedia.org/resource/Goseong_Fire_of_2019

[136] Bahk E-J. Massive wildfire wreaks havoc on Gangwon towns. The Korea Times. 2019. (accessed September 28, 2024). https://www.koreatimes.co.kr/www/nation/2024/09/281_266701.html

[137] Gabbert B. One dead, 50,000 acres burn in Substation Fire. Wildfire Today. 2018. (accessed September 28, 2024). https://wildfiretoday.com/2018/07/19/one-dead-50000-acres-burn-in-substation-fire/

[138] California Governor's Office of Emergency Services. Wildfire: The Camp Fire in Paradise. Preparedness Ambassadors Case Studies. 2020. (accessed March 31, 2026). https://www.caloes.ca.gov/wp-content/uploads/Preparedness/Documents/PA_Case_Study_1_Wildfire.pdf

[139] Canadian Disaster. Database 2018. (accessed September 28, 2024). https://cdd.publicsafety.gc.ca/dtprnt-eng.aspx?cultureCode=en-Ca&eventTypes=%27WF%27&normalizedCostYear=1&dynamic=false&eventId=1135&prnt=bot

[140] Obuobi-Donkor G, Eboreime E, Shalaby R, Agyapong B, Oluwasina F, Adu M, et al. Evaluating the prevalence and predictors of moderate to severe depression in Fort McMurray, Canada during the COVID-19 Pandemic. Int J Environ Res Public Health. 2022;19. 10.3390/ijerph19127090. 10.3390/ijerph19127090 PMC9222250 35742346

[141] Owusu E, Shalaby R, Eboreime E, Nkire N, Lawal MA, Agyapong B, et al. Prevalence and determinants of generalized anxiety disorder symptoms in residents of Fort McMurray 12 months following the 2020 flooding. Front Psychiatry. 2022;13:844907. 35815045 10.3389/fpsyt.2022.844907 PMC9263447

[142] Black Forest Fire 100%. Contained n.d. (accessed March 31, 2026). https://web.archive.org/web/20130627104508/http://www.kktv.com/home/headlines/Black-Forest-Fire-100-Contained-212417151.html

[143] Carly B. Looking Back: 10 years since royal gorge fire in Colorado. Royal Gorge & Bridge Park. 2023. (accessed March 31, 2026). https://royalgorgebridge.com/10-years-since-royal-gorge-fire-in-colorado/

[144] TPWD. Sept. 4, 2011 Bastrop wildfire n.d. (accessed March 31, 2026). https://tpwd.texas.gov/spdest/findadest/parks/bastrop/fire/

[145] George P. Bastrop fire’s apparent cause: trees hitting power lines. Austin American Statesman; 2011.

[146] Jeong Y. Five years after Wallow Fire, rebirth and regrowth. The Arizona Republic; 2016.

[147] Vaughn K. accessed September 28,. After the firestorm. Arizona highways n.d. (2024). https://www.arizonahighways.com/article/after-firestorm

[148] Deadly carmel forest fire begins. CIE 2025. (accessed March 31, 2026). https://israeled.org/carmel-forest-fire/

[149] Migrate N. Deadly forest fire in Northern Israel. NASA Science. 2010. (accessed March 31, 2026). https://science.nasa.gov/earth/earth-observatory/deadly-forest-fire-in-northern-israel-47678/

[150] Black saturday bushfires in Australia. National Geographic n.d. (accessed March 7, 2024). https://education.nationalgeographic.org/resource/black-saturday-bushfires-australia/

[151] Black Saturday bushfires. National museum of Australia n.d. (accessed March 7, 2024). https://www.nma.gov.au/defining-moments/resources/black-saturday-bushfires

[152] Jesusita Fire. California department of forestry and fire protection 2009. (accessed March 7, 2024) https://web.archive.org/web/20160304022718/http://cdfdata.fire.ca.gov/incidents/incidents_details_info?incident_id=310

[153] Stewart E. The Great Gap Fire of 2008. 2008. (accessed March 7, 2024). https://www.independent.com/2008/07/10/great-gap-fire-2008/

[154] Tea Fire. California department of forestry and fire protection n.d. (accessed March 7, 2024). https://www.fire.ca.gov/incidents/2008/11/13/tea-fire/

[155] Mitchell W. California Fire Siege 2007. California Department of Forestry and Fire Protection; 2007.

[156] Maranghides A, Mell WE. A case study of a community affected by the witch and guejito fires. Gaithersburg, MD: National Institute of Standards and Technology; 2009. 10.6028/nist.tn.1635.

[157] Greek forests severely damaged by summer fires. World Wildlife Fund 2007. (accessed March 7, 2024). https://web.archive.org/web/20071017045458/http://panda.org/news_facts/newsroom/index.cfm?uNewsID=114400

[158] Polyzoidis P. Greek lives scarred by inferno. BBC News; 2007.

[159] Rosenfeld E. Top 10 Devastating Wildfires. Time; 2011.

[160] McLean R. Wildfires in Spain blamed on arson - Europe - International Herald Tribune. The New York Times. 2006.

[161] Sokolovsky J. Authorities seek cause of Spanish wildfires. NPR; 2006.

[162] Bushfire - Eyre Peninsula, South, Australia. accessed March 31, n.d. (2026). https://knowledge.aidr.org.au/resources/bushfire-eyre-peninsula-south-australia/

[163] Southern California wildfires October 20 to November 3. 2003 2004. (accessed March 31, 2026). http://repository.library.noaa.gov/view/noaa/6470

[164] Forest PP. 2003 Firestorm provincial review. Forest protection program; 2004.

[165] British Columbia 2003 Forest Fires. VALOUR CANADA 2020. (accessed March 31, 2026). https://valourcanada.ca/military-history-library/british-columbia-2003-forest-fires/

[166] Canberra bushfires. National Museum of Australia n.d. (accessed March 7, 2024). https://www.nma.gov.au/defining-moments/resources/canberra-bushfires

[167] Thirty Years Since the 1994 bushfires. Museum of Fire 2024. (accessed March 31, 2026). https://www.museumoffire.net/single-post/thirty-years-since-the-1994-bushfires

[168] Powell A. 1990 Paint Fire. Santa Barbara Bucket Brigade 2019. (accessed March 7, 2024). https://sbbucketbrigade.org/timeline/1990-paint-fire/

[169] Bushfire - Canberra. 2003. Australian institute for disaster resilience n.d. (accessed March 7, 2024). https://knowledge.aidr.org.au/resources/bushfire-canberra-2003/

[170] Forest Fire Management Victoria. Ash Wednesday. 1983. Forest fire management victoria 2023. (accessed March 31, 2026). https://www.ffm.vic.gov.au/history-and-incidents/ash-wednesday-1983

[171] Depression and other common mental disorders. Global health estimates. Geneva: World health organization; 2017.

[172] Uher R, Payne JL, Pavlova B, Perlis RH. Major depressive disorder in DSM-5: implications for clinical practice and research of changes from DSM-IV: Review: Major depressive disorder in DSM-5. Depress Anxiety. 2014;31:459–71. 24272961 10.1002/da.22217

[173] Craske MG, Rauch SL, Ursano R, Prenoveau J, Pine DS, Zinbarg RE. What is an anxiety disorder? Focus (Am Psychiatr Publ). 2011;9:369–88.

[174] DeMartini J, Patel G, Fancher TL. Generalized anxiety disorder. Ann Intern Med. 2019;170:ITC49–64. 30934083 10.7326/AITC201904020

[175] Javaid SF, Hashim IJ, Hashim MJ, Stip E, Samad MA, Ahbabi AA. Epidemiology of anxiety disorders: global burden and sociodemographic associations. Middle East Curr Psychiatr. 2023;30:1–11.

[176] Center for Substance Abuse Treatment (US). Exhibit 1.3-4, DSM-5 Diagnostic Criteria for PTSD 2014.

[177] Koenen KC, Ratanatharathorn A, Ng L, McLaughlin KA, Bromet EJ, Stein DJ, et al. Posttraumatic stress disorder in the world mental health surveys. Psychol Med. 2017;47:2260–74. 28385165 10.1017/S0033291717000708 PMC6034513

[178] Zhu Y, Jha SC, Shutta KH, Huang T, Balasubramanian R, Clish CB, et al. Psychological distress and metabolomic markers: A systematic review of posttraumatic stress disorder, anxiety, and subclinical distress. Neurosci Biobehav Rev. 2022;143:104954. 36368524 10.1016/j.neubiorev.2022.104954 PMC9729460

[179] Piao X, Xie J, Managi S. Continuous worsening of population emotional stress globally: universality and variations. BMC Public Health. 2024;24:3576. 39716139 10.1186/s12889-024-20961-4 PMC11668040

[180] Substance use and co-occurring mental disorders. National institute of mental health (NIMH). 2024. (accessed February 4, 2025). (accessed February 4, 2025). https://www.nimh.nih.gov/health/topics/substance-use-and-mental-health

[181] Castaldelli-Maia JM, Bhugra D. Analysis of global prevalence of mental and substance use disorders within countries: focus on sociodemographic characteristics and income levels. Int Rev Psychiatry. 2022;34:6–15. 35584016 10.1080/09540261.2022.2040450

[182] Southwick SM, Bonanno GA, Masten AS, Panter-Brick C, Yehuda R. Resilience definitions, theory, and challenges: interdisciplinary perspectives. Eur J Psychotraumatol. 2014;5:25338. 10.3402/ejpt.v5.25338 PMC4185134 25317257

[183] Resilience. Https://www.apa.org2022. https://www.apa.org/topics/resilience (accessed January 29, 2025).

[184] Chua JH, Cheng CKT, Cheng LJ, Ang WHD, Lau Y. Global prevalence of resilience in higher education students: A systematic review, meta-analysis and meta-regression. Curr Psychol. 2023;42:22645–63.

[185] Cheng CKT, Chua JH, Cheng LJ, Ang WHD, Lau Y. Global prevalence of resilience in health care professionals: A systematic review, meta-analysis and meta-regression. J Nurs Manag. 2022;30:795–816. 35130583 10.1111/jonm.13558

[186] Bhaskar S, Hemavathy D, Prasad S. Prevalence of chronic insomnia in adult patients and its correlation with medical comorbidities. J Family Med Prim Care. 2016;5:780–4. 28348990 10.4103/2249-4863.201153 PMC5353813

[187] Suicidality APA, Dictionary of. Psychology 2018. https://dictionary.apa.org/ (accessed April 7, 2025).

[188] Lim K-S, Wong CH, McIntyre RS, Wang J, Zhang Z, Tran BX, et al. Global lifetime and 12-month prevalence of suicidal behavior, deliberate self-harm and non-suicidal self-injury in children and adolescents between 1989 and 2018: A meta-analysis. Int J Environ Res Public Health. 2019;16:4581. 31752375 10.3390/ijerph16224581 PMC6888476

[189] Bailey JA. The foundation of self-esteem. J Natl Med Assoc. 2003;95:388–93. 12793795 PMC2594522

[190] Tedeschi RG, Calhoun LG. The Posttraumatic Growth Inventory: measuring the positive legacy of trauma. J Trauma Stress. 1996;9:455–71. 8827649 10.1007/BF02103658

[191] Gardner FL, Moore ZE. Understanding clinical anger and violence: the anger avoidance model: The Anger Avoidance Model. Behav Modif. 2008;32:897–912. 18614696 10.1177/0145445508319282

[192] Okuda M, Picazo J, Olfson M, Hasin DS, Liu S-M, Bernardi S, et al. Prevalence and correlates of anger in the community: results from a national survey. CNS Spectr. 2015;20:130–9. 25831968 10.1017/S1092852914000182 PMC4384185

[193] Painuly NP, Grover S, Gupta N, Mattoo SK. Prevalence of anger attacks in depressive and anxiety disorders: implications for their construct? Anger attacks. Psychiatry Clin Neurosci. 2011;65:165–74. 21232077 10.1111/j.1440-1819.2010.02177.x

[194] Paranoid State. American psychological association. 2018. / (accessed April 14, 2025). https://dictionary.apa.org

[195] Collin S, Rowse G, Martinez AP, Bentall RP. Delusions and the dilemmas of life: A systematic review and meta-analyses of the global literature on the prevalence of delusional themes in clinical groups. Clin Psychol Rev. 2023;104:102303. 37390804 10.1016/j.cpr.2023.102303

[196] Winstock AR, Barratt MJ. The 12-month prevalence and nature of adverse experiences resulting in emergency medical presentations associated with the use of synthetic cannabinoid products: Adverse experiences with synthetic cannabinoids. Hum Psychopharmacol. 2013;28:390–3. 23881887 10.1002/hup.2292

[197] Tedeschi RG, Shakespeare-Finch J, Taku K, Calhoun LG. Posttraumatic growth: Theory, research, and applications. 1st Edition. New York, NY: Routledge, 2018.: Routledge; 2018.

[198] Wu X, Kaminga AC, Dai W, Deng J, Wang Z, Pan X, et al. The prevalence of moderate-to-high posttraumatic growth: A systematic review and meta-analysis. J Affect Disord. 2019;243:408–15. 30268956 10.1016/j.jad.2018.09.023

[199] Gautam S, Jain A, Chaudhary J, Gautam M, Gaur M, Grover S. Concept of mental health and mental well-being, it’s determinants and coping strategies. Indian J Psychiatry. 2024;66:S231–44. 38445271 10.4103/indianjpsychiatry.indianjpsychiatry_707_23 PMC10911315

[200] Fan Y, Fan A, Yang Z, Fan D. Global burden of mental disorders in 204 countries and territories, 1990–2021: results from the global burden of disease study 2021. BMC Psychiatry. 2025;25:486. 40375174 10.1186/s12888-025-06932-y PMC12080068

[201] Hansen M, Andersen TE, Armour C, Elklit A, Palic S, Mackrill T. PTSD-8: A short PTSD inventory. Clin Pract Epidemiol Ment Health. 2010;6:101–8. 21253461 10.2174/1745017901006010101 PMC3023946

[202] Jones MW, Abatzoglou JT, Veraverbeke S, Andela N, Lasslop G, Forkel M, et al. Global and regional trends and drivers of fire under climate change. Rev Geophys. 2022;60:e2020RG000726.

[203] Johnson J. accessed February 2,. This is what Paradise looks like five years after devastating Camp Fire 2023. (2024). https://www.sfchronicle.com/california-wildfires/article/camp-fire-anniversary-18442613.php

[204] Magzamen S, Gan RW, Liu J, O’Dell K, Ford B, Berg K et al. Differential cardiopulmonary health impacts of local and long-range transport of wildfire smoke. GeoHealth. 2021;5(3):e2020GH000330. 10.1029/2020GH000330 PMC8900982 35281479

[205] Ballesteros-González K, Sullivan AP, Morales-Betancourt R. Estimating the air quality and health impacts of biomass burning in northern South America using a chemical transport model. Sci Total Environ. 2020;739:139755. 32758934 10.1016/j.scitotenv.2020.139755

[206] Mirzaei M, Bertazzon S, Couloigner I. Modeling wildfire smoke pollution by integrating land use regression and remote sensing data: Regional multi-temporal estimates for public health and exposure models. Atmos (Basel). 2018;9:335.

[207] US EPA. The Inside Story: A guide to indoor air quality 2014. (accessed June 21, 2024). https://www.epa.gov/indoor-air-quality-iaq/inside-story-guide-indoor-air-quality

[208] Liu X-Q, Huang J, Song C, Zhang T-L, Liu Y-P, Yu L. Neurodevelopmental toxicity induced by PM2.5 Exposure and its possible role in Neurodegenerative and mental disorders. Hum Exp Toxicol. 2023;42:9603271231191436. 37537902 10.1177/09603271231191436

[209] White AR. The firestorm within: A narrative review of extreme heat and wildfire smoke effects on brain health. Sci Total Environ. 2024;922:171239. 38417511 10.1016/j.scitotenv.2024.171239

[210] U.N. warns of global mental health crisis due to COVID-19 pandemic. American Psychological Association; 2020.

[211] Clayton S. Climate anxiety: Psychological responses to climate change. J Anxiety Disord. 2020;74:102263. 32623280 10.1016/j.janxdis.2020.102263

[212] Palinkas LA, Wong M. Global climate change and mental health. Curr Opin Psychol. 2020;32:12–6. 31349129 10.1016/j.copsyc.2019.06.023

[213] Humphreys A, Walker EG, Bratman GN, Errett NA. What can we do when the smoke rolls in? An exploratory qualitative analysis of the impacts of rural wildfire smoke on mental health and wellbeing, and opportunities for adaptation. BMC Public Health. 2022;22:41. 34991532 10.1186/s12889-021-12411-2 PMC8740038

[214] Ferrarello S. Solastalgia: Climatic Anxiety-An Emotional Geography to Find Our Way Out. J Med Philos. 2023;48:151–60. 37078732 10.1093/jmp/jhad006

[215] McLeish AC, Del Ben KS. Symptoms of depression and posttraumatic stress disorder in an outpatient population before and after Hurricane Katrina. Depress Anxiety. 2008;25:416–21. 17969132 10.1002/da.20426

[216] Dominguez-Rafer C, Lin S. What are the sensitivity and specificity of the PHQ-2 and the PHQ-9 in screening for depression? Evidence-Based Pract. 2011;14:8.

[217] Wimalawansa SJ. Mechanisms of developing post-traumatic stress disorder: new targets for drug development and other potential interventions. CNS Neurol Disord Drug Targets. 2014;13:807–16. 25012621 10.2174/1871527313666140711091026

[218] Neria Y, Nandi A, Galea S. Post-traumatic stress disorder following disasters: a systematic review. Psychol Med. 2008;38:467–80. 17803838 10.1017/S0033291707001353 PMC4877688

[219] Wang Z, Wu X, Dai W, Kaminga AC, Wu X, Pan X, et al. The prevalence of posttraumatic stress disorder among survivors after a typhoon or hurricane: A systematic review and meta-analysis. Disaster Med Public Health Prep. 2019;13:1065–73. 31204633 10.1017/dmp.2019.26

[220] Galea S, Brewin CR, Gruber M, Jones RT, King DW, King LA, et al. Exposure to hurricane-related stressors and mental illness after Hurricane Katrina. Arch Gen Psychiatry. 2007;64:1427–34. 18056551 10.1001/archpsyc.64.12.1427 PMC2174368

[221] Gradus JL, Galea S. Moving from traumatic events to traumatic experiences in the study of traumatic psychopathology. Am J Epidemiol. 2023;192:1609–12. 37218615 10.1093/aje/kwad126 PMC10558183

[222] Spinhoven P, Penninx BW, van Hemert AM, de Rooij M, Elzinga BM. Comorbidity of PTSD in anxiety and depressive disorders: prevalence and shared risk factors. Child Abuse Negl. 2014;38:1320–30. 24629482 10.1016/j.chiabu.2014.01.017

[223] McDonald SD, Calhoun PS. The diagnostic accuracy of the PTSD checklist: a critical review. Clin Psychol Rev. 2010;30:976–87. 20705376 10.1016/j.cpr.2010.06.012

[224] What are anxiety disorders? American psychiatric association. n.d. (accessed January 11, 2025). https://www.psychiatry.org:443/patients-families/anxiety-disorders/what-are-anxiety-disorders

[225] Anderson TL, Valiauga R, Tallo C, Hong CB, Manoranjithan S, Domingo C, et al. Contributing factors to the rise in adolescent anxiety and associated mental health disorders: A narrative review of current literature. J Child Adolesc Psychiatr Nurs. 2025;38:e70009. 39739929 10.1111/jcap.70009 PMC11683866

[226] Hickman C, Marks E, Pihkala P, Clayton S, Lewandowski RE, Mayall EE, et al. Climate anxiety in children and young people and their beliefs about government responses to climate change: a global survey. Lancet Planet Health. 2021;5:e863–73. 34895496 10.1016/S2542-5196(21)00278-3

[227] Whitmarsh L, Player L, Jiongco A, James M, Williams M, Marks E, et al. Climate anxiety: What predicts it and how is it related to climate action? J Environ Psychol. 2022;83:101866.

[228] Kar N, Bastia BK. Post-traumatic stress disorder, depression and generalised anxiety disorder in adolescents after a natural disaster: a study of comorbidity. Clin Pract Epidemiol Ment Health. 2006;2:17. 16869979 10.1186/1745-0179-2-17 PMC1563462

[229] Ruggiero KJ, Amstadter AB, Acierno R, Kilpatrick DG, Resnick HS, Tracy M, et al. Social and psychological resources associated with health status in a representative sample of adults affected by the 2004 Florida hurricanes. Psychiatry. 2009;72:195–210. 19614556 10.1521/psyc.2009.72.2.195 PMC2792984

[230] Schwartz RM, Gillezeau CN, Liu B, Lieberman-Cribbin W, Taioli E. Longitudinal impact of Hurricane Sandy exposure on mental health symptoms. Int J Environ Res Public Health. 2017;14:957. 28837111 10.3390/ijerph14090957 PMC5615494

[231] Amstadter AB, Koenen KC, Ruggiero KJ, Acierno R, Galea S, Kilpatrick DG, et al. NPY moderates the relation between hurricane exposure and generalized anxiety disorder in an epidemiologic sample of hurricane-exposed adults. Depress Anxiety. 2010;27:270–5. 20037921 10.1002/da.20648 PMC2879267

[232] Ware JE, Sherbourne CD. The MOS 36-ltem short-form health survey (SF-36): I. conceptual framework and item selection. Med Care. 1992;30:473–83. 1593914

[233] Hrabok M, Delorme A, Agyapong VIO. Threats to mental health and well-being associated with climate change. J Anxiety Disord. 2020;76:102295. 32896782 10.1016/j.janxdis.2020.102295

[234] Kessler RC, Andrews G, Colpe LJ, Hiripi E, Mroczek DK, Normand SLT, et al. Short screening scales to monitor population prevalences and trends in non-specific psychological distress. Psychol Med. 2002;32:959–76. 12214795 10.1017/s0033291702006074

[235] Morin CM, Belleville G, Bélanger L, Ivers H. The Insomnia Severity Index: psychometric indicators to detect insomnia cases and evaluate treatment response. Sleep. 2011;34:601–8. 21532953 10.1093/sleep/34.5.601 PMC3079939

[236] Carver CS, Scheier MF, Weintraub JK. Assessing coping strategies: A theoretically based approach. J Pers Soc Psychol. 1989;56:267–83. 2926629 10.1037//0022-3514.56.2.267

[237] Wildfires, substance abuse and mental health services administration. 2023. (accessed November 4, 2023). https://www.samhsa.gov/find-help/disaster-distress-helpline/disaster-types/wildfires

[238] Carl Y, Vega A, Cardona-Acevedo G, Stukova M, Matos-Rivera M, Torres-Sanchez A, et al. Post-hurricane distress scale (PHDS): Determination of general and disorder-specific cutoff scores. Int J Environ Res Public Health. 2022;19:5204. 35564598 10.3390/ijerph19095204 PMC9100105

[239] Liu L, Loftus C, Rohlman D, Seto E, Austin E. Development of a questionnaire library for rapid health data acquisition during wildfire events. MethodsX. 2025;14:103396. 40529519 10.1016/j.mex.2025.103396 PMC12171533

[240] Montesanti S, Fitzpatrick K, Azimi T, McGee T, Fayant B, Albert L. Exploring indigenous ways of coping after a wildfire disaster in Northern Alberta, Canada. Qual Health Res. 2021;31:1472–85. 33971774 10.1177/10497323211009194 PMC8278559

[241] Coping with wildfires and climate change crises. UCSF Department of psychiatry and behavioral sciences. 2020. (accessed November 6, 2023). https://psychiatry.ucsf.edu/copingresources/wildfires

[242] Markusoff J. Why Fort McMurray will never be the same. Maclean’s Inc. 2017. (accessed September 14, 2025). https://macleans.ca/news/canada/why-fort-mcmurray-will-never-be-the-same/

[243] Wildland fire summary and statistics annual report. National interagency coordination center; 2023.

[244] Chen G, Guo Y, Yue X, Tong S, Gasparrini A, Bell ML, et al. Mortality risk attributable to wildfire-related PM2·5 pollution: a global time series study in 749 locations. Lancet Planet Health. 2021;5:e579–87. 34508679 10.1016/S2542-5196(21)00200-X

[245] An X, Kang N, Li P, Tong M, Li F, Tong J, et al. Estimating burden of anemia in women of reproductive age attributable to wildfire-sourced fine particulate matters: A multi-center epidemiological study in low-and-middle income countries. Social Sci Res Netw. 2024. 10.2139/ssrn.4996817.

[246] Casey JA, Schwartz BS, Stewart WF, Adler NE. Using electronic health records for population health research: A review of methods and applications. Annu Rev Public Health. 2016;37:61–81. 26667605 10.1146/annurev-publhealth-032315-021353 PMC6724703

[247] Alibudbud R. Google Trends for health research: Its advantages, application, methodological considerations, and limitations in psychiatric and mental health infodemiology. Front Big Data. 2023;6:1132764. 37050919 10.3389/fdata.2023.1132764 PMC10083382

[248] Hahn MB, Michlig GJ, Hansen A, Manning L, Augustinavicius JL. Mental health during wildfires in Southcentral Alaska: An assessment of community-derived mental health categories, interventions, and implementation considerations. PLOS Clim. 2023;2:e0000300.

[249] Morales DA, Barksdale CL, Beckel-Mitchener AC. A call to action to address rural mental health disparities. J Clin Transl Sci. 2020;4:463–7. 33244437 10.1017/cts.2020.42 PMC7681156

[250] Horowitz M, Wilner N, Alvarez W. Impact of Event Scale: a measure of subjective stress. Psychosom Med. 1979;41(3):209-218. 10.1097/00006842-197905000-00004 472086

